# Safety and Efficacy of Riluzole in Acute Spinal Cord Injury Study (RISCIS): A Multi-Center, Randomized, Placebo-Controlled, Double-Blinded Trial

**DOI:** 10.1089/neu.2023.0163

**Published:** 2023-08-23

**Authors:** Michael G. Fehlings, Ali Moghaddamjou, James S. Harrop, Ralph Stanford, Jonathon Ball, Bizhan Aarabi, Brian J. C. Freeman, Paul M. Arnold, James D. Guest, Shekar N. Kurpad, James M. Schuster, Ahmad Nassr, Karl M. Schmitt, Jefferson R. Wilson, Darrel S. Brodke, Faiz U. Ahmad, Albert Yee, Wilson Z. Ray, Nathaniel P. Brooks, Jason Wilson, Diana S-L Chow, Elizabeth G. Toups, Branko Kopjar

**Affiliations:** ^1^ Division of Neurosurgery, Department of Surgery, University of Toronto, Toronto, Ontario, Canada.; ^2^ Division of Neurosurgery, Krembil Neuroscience Center, Toronto Western Hospital, University Health Network, Toronto, Ontario, Canada.; ^3^ Department of Neurological Surgery, Thomas Jefferson University, Philadelphia, Pennsylvania, USA.; ^4^ Neuroscience Research Australia and Prince of Wales Hospital, Sydney, New South Wales, Australia.; ^5^ Royal North Shore Hospital, St. Leonards, New South Wales, Australia.; ^6^ Department of Neurosurgery, University of Maryland School of Medicine, Baltimore, Maryland, USA.; ^7^ Center for Orthopedic and Trauma Research, Adelaide Medical School, the University of Adelaide, Adelaide, South Australia, Australia.; ^8^ Carle Illinois College of Medicine, University of Illinois Urbana-Champaign, Champaign, Illinois, USA.; ^9^ Department of Neurosurgery and the Miami Project to Cure Paralysis, the Miller School of Medicine, University of Miami, Miami, Florida, USA.; ^10^ Department of Neurosurgery, Medical College of Wisconsin, Wauwatosa, Wisconsin, USA.; ^11^ Department of Neurosurgery, Pennsylvania Hospital, University of Pennsylvania Health System, Philadelphia, Pennsylvania, USA.; ^12^ Department of Orthopedic Surgery, Mayo Clinic, Rochester, Minnesota, USA.; ^13^ Department of Neurosurgery, Health Science Center, University of Texas, Houston, Texas, USA.; ^14^ Department of Orthopedics, University of Utah, Salt Lake City, Utah, USA.; ^15^ Department of Neurological Surgery, Emory University School of Medicine, Atlanta, Georgia, USA.; ^16^ Department of Neurosurgery, Washington University, St. Louis, Missouri, USA.; ^17^ Department of Neurological Surgery, University of Wisconsin School of Medicine and Public Health, Madison, Wisconsin, USA.; ^18^ Department of Neurosurgery and School of Medicine, Louisiana State University Health Sciences Center, New Orleans, Louisiana, USA.; ^19^ Department of Pharmacological and Pharmaceutical Sciences, College of Pharmacy, University of Houston, Houston, Texas, USA.; ^20^ Department of Neurosurgery, Houston Methodist Hospital, Houston, Texas, USA.; ^21^ Department of Health Services, University of Washington, Seattle, Washington, USA.

**Keywords:** clinical trial, glutamate antagonist, neuroprotection, neurotrauma, sodium channel blocker, spinal cord injury

## Abstract

Riluzole is a sodium-glutamate antagonist that attenuates neurodegeneration in amyotrophic lateral sclerosis (ALS). It has shown favorable results in promoting recovery in pre-clinical models of traumatic spinal cord injury (tSCI) and in early phase clinical trials. This study aimed to evaluate the efficacy and safety of riluzole in acute cervical tSCI. An international, multi-center, prospective, randomized, double-blinded, placebo-controlled, adaptive, Phase III trial (NCT01597518) was undertaken. Patients with American Spinal Injury Association Impairment Scale (AIS) A-C, cervical (C4-C8) tSCI, and <12 h from injury were randomized to receive either riluzole, at an oral dose of 100 mg twice per day (BID) for the first 24 h followed by 50 mg BID for the following 13 days, or placebo. The primary efficacy end-point was change in Upper Extremity Motor (UEM) scores at 180 days. The primary efficacy analyses were conducted on an intention to treat (ITT) and completed cases (CC) basis. The study was powered at a planned enrolment of 351 patients. The trial began in October 2013 and was halted by the sponsor on May 2020 (and terminated in April 2021) in the face of the global COVID-19 pandemic. One hundred ninety-three patients (54.9% of the pre-planned enrolment) were randomized with a follow-up rate of 82.7% at 180 days. At 180 days, in the CC population the riluzole-treated patients compared with placebo had a mean gain of 1.76 UEM scores (95% confidence interval: -2.54-6.06) and 2.86 total motor scores (CI: -6.79-12.52). No drug-related serious adverse events were associated with the use of riluzole. Additional pre-planned sensitivity analyses revealed that in the AIS C population, riluzole was associated with significant improvement in total motor scores (estimate: standard error [SE] 8.0; CI 1.5-14.4) and upper extremity motor scores (SE 13.8; CI 3.1-24.5) at 6 months. AIS B patients had higher reported independence, measured by the Spinal Cord Independence Measure score (45.3 vs. 27.3; d: 18.0 CI: -1.7-38.0) and change in mental health scores, measured by the Short Form 36 mental health domain (2.01 vs. -11.58; d: 13.2 CI: 1.2-24.8) at 180 days. AIS A patients who received riluzole had a higher average gain in neurological levels at 6 months compared with placebo (mean 0.50 levels gained vs. 0.12 in placebo; d: 0.38, CI: -0.2-0.9). The primary analysis did not achieve the predetermined end-point of efficacy for riluzole, likely related to insufficient power. However, on pre-planned secondary analyses, all subgroups of cervical SCI subjects (AIS grades A, B and C) treated with riluzole showed significant gains in functional recovery. The results of this trial may warrant further investigation to extend these findings. Moreover, guideline development groups may wish to assess the possible clinical relevance of the secondary outcome analyses, in light of the fact that SCI is an uncommon orphan disorder without an accepted neuroprotective treatment.

## Introduction

Traumatic spinal cord injury (tSCI) is a life-altering event that has a significant impact on patients, their families, and the healthcare system. The annual incidence of tSCI also varies depending on the region considered, with worldwide estimates ranging from 10 to 85 per million individuals.^1–4^

Despite the immense impact of tSCI at a personal and societal level, an effective and safe pharmacological treatment for tSCI that improves neurological and functional outcomes has yet to be identified. Clinical trials with methylprednisolone (National Acute Spinal Cord Injury Study [NASCIS] II and III)^5,6^ and GM-1 ganglioside^7^ have provided suggestive but equivocal evidence of benefit.

Riluzole is a sodium channel blocking benzothiazole anticonvulsant that exerts its neuroprotective effect by helping maintain neuronal cellular ionic balance and by reducing the release of excitotoxic glutamate following tSCI.^8^ Riluzole has been widely used in the treatment of amyotrophic lateral sclerosis (ALS), a progressive neurodegenerative disorder characterized by motor neuron and corticospinal tract degeneration.^9-11^ Notably, riluzole is without potent neurotoxic and cardiotoxic adverse effects, although reversible hepatotoxicity has been reported.^10^ Riluzole is approved by the U. S. Food and Drug Administration for the chronic treatment of patients with ALS.^12^

There is strong evidence from pre-clinical studies that riluzole attenuates the secondary injury cascade leading to diminished neurological tissue destruction in animal tSCI models.^13–16^ After tSCI, there is an increase in cell membrane permeability and synaptic glutamate release, which results in prolonged excitability of postsynaptic neurons leading to neuronal edema and death. Through inhibition of voltage-gated sodium channels, riluzole inhibits glutamate release and can hence mitigate excitotoxicity. The rationale for the trial was published in advance by the North American Clinical Trial Network.^17^

The clinical safety and pharmacokinetic profile of riluzole were examined in a Phase I/IIa multi-center pilot study in the context of tSCI.^18^ The 12-h dosing window, as well as the 2-week duration of the therapy, was chosen to match the period of medication administration with the known period of glutamatergic excitotoxicity after SCI (several minutes after injury until 2 weeks after injury).^19^ The pharmacodynamics and pharmacokinetics of enterally administered riluzole in tSCI patients were established prior to the study.^20^ Completion of the Phase I/IIa study confirmed the acceptable safety profile of riluzole administration, provided pilot data suggesting a treatment benefit of riluzole in tSCI, and established the feasibility of conducting a large-scale efficacy trial investigating riluzole as a potential treatment for acute tSCI.

## Methods

### Eligibility and recruitment

Participants with acute cervical tSCI who presented to a participating hospital site within 12 h of injury were screened for inclusion. Before any screening procedures, participants were required to sign an Informed Consent Document. Patients between the ages of 18-75 (inclusive) with a Neurological Level of Injury between C4-C8, American Spinal Cord Injury Association Impairment Scale (AIS) grade “A,” “B,” or “C” based upon the first International Standards for Neurological Classification of Spinal Cord Injury (ISNCSCI) evaluation after arrival, and who were able to receive the investigational drug within the first 12 h of injury were considered for inclusion. The full list of inclusion and exclusion criteria is outlined in Table S1 in the Supplementary Material. The study utilized a randomization ratio of one riluzole subject to one placebo subject (1:1).

### Study intervention

The riluzole group received riluzole in a dose of 100 mg at enrollment (0 h) and 12 h (doses 1 and 2) followed by 50 mg doses every 12 h for the next 13 days (doses 3-28). The control group received placebo capsules in a dose of two capsules at 0 h and 12 h (doses 1 and 2) followed by one capsule every 12 h for the next 13 days (doses 3-28). Treatments other than the randomly allocated study medication were at the attending physicians' discretion, including type and timing of surgery and medical interventions. However, all investigators agreed to a principle of early surgical intervention within 24 h and to follow existing guidelines for tSCI management.

### Follow-up evaluation

Patient evaluation was conducted by study personnel who were qualified by appropriate training on the study protocols and ISNCSCI examination, and all follow-up visits were preferred to be performed in the outpatient clinic. However, if the subject was unable to visit the clinic, a study investigator or an assigned examiner could visit the subject to collect the data. Data collection by telephone and/or mail was not permitted. The study treatment and data collection for each scheduled visit are summarized in Table S2 in the Supplementary Material. The study continued until the last enrolled subject reached the 180-day follow-up. As a consequence, a portion of participants did not have a 365-day follow-up visit.

### Study end-points

Prior to unblinding and data analysis, the study group decided to change the primary efficacy end-point to Upper Extremity Motor (UEM) from total motor (TOTM) score change at 180 days. Since the development of the Riluzole in Acute Spinal Cord Injury Study (RISCIS) trial, evidence has emerged from clinical and research communities on the utility of the UEM score change as the primary end-point to allow for a focused assessment of hand and arm control.^21^ Similarly, a decision was made to replace the sensory component of the ISNCSCI exam with changes in AIS grade in the secondary efficacy end-points. This decision was made by the study group due to consensus on the lack of utility and reliability of the ISNCSCI sensory scores as a valid measure of classification of tSCI.^22^ The ISNCSCI TOTM and sensory scores were retained as secondary end-points of potential efficacy.

Other secondary efficacy end-points included the Spinal Cord Independence Measure (SCIM) at 180 days from baseline and changes between baseline and 180 days in ISNCSCI sensory scores, Lower Extremity Motor (LEM) scores, Short Form 36 Version 2 (SF-36v2™), European Quality of Life 5 Dimensions 3 Level Version health utility, Graded Redefined Assessment of Strength Sensibility and Prehension (GRASSP), and self- reported Numeric Pain Rating Scale (pain NRS) scores.

All adverse events were coded using MeDRA Version 14.1. The incidence of adverse events in each treatment group were summarized by system organ class, preferred term, severity, relation to the study drug, outcome and actions taken and time-to-event.

### Analysis samples/population

The original estimated sample size for the study was calculated to be 316. In this international multi-center study, all sites followed the exact same protocol and were subjected to monitoring and validation. Data gathered from sites were treated as one cohort.

The primary analysis was conducted in both the intent-to-treat (ITT) and completed cases (CC) population. The ITT was defined to include all participants who were randomized to a study arm and received at least one dose of the investigational product. Values for participants who did not have their 180-day follow-up were imputed to create a complete ITT population. The methodology for the imputation is described in the Supplementary Material. The CC population is defined to include all participants who received at least one dose of the study treatment and who had an efficacy measurement taken at the 180-day follow-up. Safety analyses were conducted on the modified ITT population defined to include all consenting subjects who received at least one dose of the study-directed treatment that had any follow-up.

### Statistical analysis

Study success was defined as follows: investigational treatment (riluzole) was superior to placebo. The statistical approach used was to test a single one-sided null hypothesis that the difference between the investigational and the placebo arms was equal to or less than 0. Rejection of the null hypothesis was consistent with the superiority of the investigational treatment to the placebo. The statistical significance was established at alpha = 0.025.

Testing for all secondary outcomes was based on appropriate statistical methods and one-sided superiority testing. A pre-planned subgroup analysis was conducted to evaluate differences in change in motor scores at 180 days among the patients in baseline AIS groups “A,” “B,” and “C.” All statistical analyses were conducted in RStudio version 1.4.1103

### Interim analysis and stopping rules

Enrollment of the trial was halted prematurely by the sponsor on May 1, 2020, in the face of the global COVID pandemic. At that time, 193 subjects had been enrolled in the study. Given that the trial had almost reached the sample size for the protocol-prescribed pre-planned interim analysis (60%), the Data and Safety Monitoring Board (DSMB) recommended to the sponsor to proceed with the interim analysis.

The independent DSMB statistician recommended to switch the design to a non-binding futility hypothesis (H1) rejection from the original sequential design described in the statistical analysis plan. The rationale was to allow the DSMB to review a series of sensitivity analyses rather than using a single binding approach. This change resulted in an increase in the sample size from 316 to 324.

A recommendation to stop the study was intended to be provided if the criteria for statistical success were reached or the test values reached the established futility boundary. The threshold values for futility and superiority for both the CC and ITT population are tabulated in Table S3 in the Supplementary Material. The updated parameters of the sequential design of the trial are tabulated in Table S4 in the Supplementary Material.

The decision to halt the trial in April 2021 was made during the ongoing COVID-19 pandemic in the face of safety concerns for patients and research personnel from in-person exposure. At the time there was significant uncertainty regarding the feasibility of in-person clinical research and the outlook of the pandemic. As a result, the sponsor made the difficult decision to halt the trial. This decision was made independent of the recommendations of the DSMB and the results of the interim analysis. Additional details on the methodology are summarized in the attached Supplementary Material.

## Results

Screening and enrollment in the RISCIS trial were suspended by the Sponsor (AO Spine North America/AOSNA) on May 1, 2020. From the start of the study in October of 2013, 193 participants had been enrolled (55% of the pre-planned sample size) from 21 clinical sites across North America and Australia.

Upon evaluation of the AIS examination results following enrollment and randomization, two patients were identified as having a C3 level of injury. Although this constituted a deviation from the established protocol, the decision was made to incorporate these patients into the analysis. Further, another patient was discovered to have a T1 level of injury during the review process. This individual was lost to follow-up and not included in the final analysis.

**FIG. 1.**
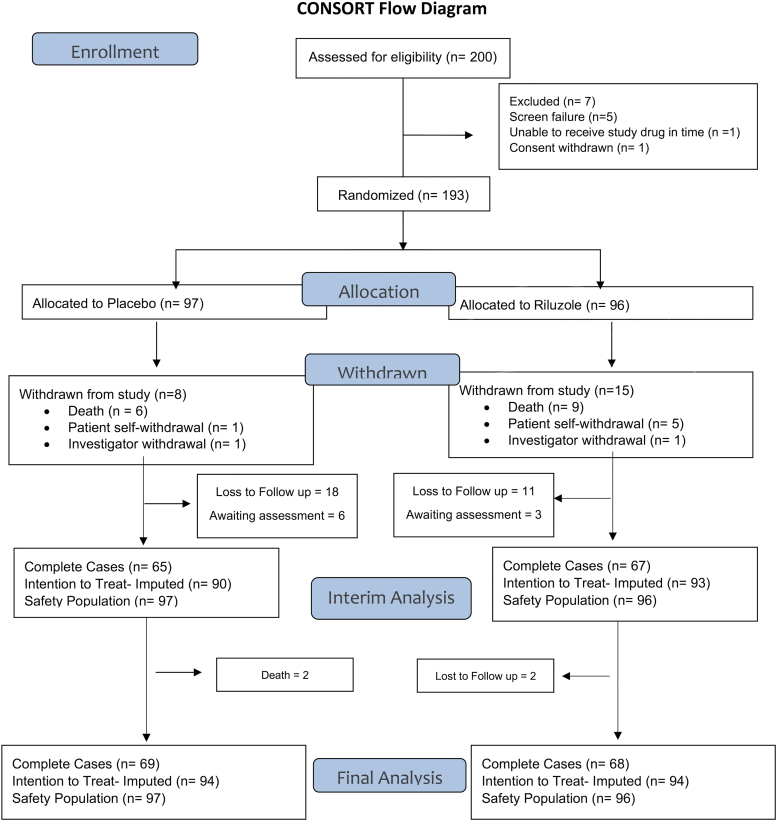
Consolidated Standards of Reporting Trials (CONSORT) flow diagram of the Riluzole in Spinal Cord Injury Study (RISCIS) at the 180-day follow-up visit.

The follow-up rate at the 180-day visit was 82.7% (139 participants) and at 365-day the follow-up rate was 82.4% (131 participants; Table S5 in the Supplementary Material). The follow-up rate was similar between riluzole and placebo groups. At the 180-day follow-up, 69 of 81 (85.19%) of the expected riluzole participants and 70 of 87 (80.46%) of the expected placebo participants attended the visit. The Consolidated Standards of Reporting Trials (CONSORT) flow diagram for subject accounting is illustrated in Figure 1.

Table 1 summarizes the demographic characteristics by treatment arm. In the treatment group, the mean age was 49.4 years (standard deviation [SD] = 17.1, range 19-74 years) with 17 females and 79 males. In the control group, the mean age was 47.6 years (SD = 16.0, range 20-74 years) with 18 females and 79 males. Most patients had an AIS A grade injury (49 or 51.58% of patients in the riluzole group and 52 or 53.61% of patients in the placebo group). In the AIS B and C groups there was an equal distribution of patients between the riluzole and placebo groups, with 19 patients in each group for the AIS B cohort and 26 patients in each group for the AIS C cohort. The majority of the patients (94.62%) received surgical decompression within 24 h.

**Table 1.** Demographic and Injury Characteristics of Patients Enrolled in the RISCIS Study

	Riluzole (*n* = 96)	Control (*n* = 97)
Age at consent (years)		
*n*	96	97
Mean	49.4	47.6
SD	17.1	16.0
Sex, *n* (%)		
Male	79 (82.3)	79 (81.4)
Female	17 (17.7)	18 (18.6)
Race, *n* (%)		
White	69 (71.9)	71 (73.2)
Black or African American	13 (13.5)	15(15.5)
Asian	10 (10.4)	7 (7.2)
Native Hawaiian or other Pacific Islander	1 (1.0)	0
American Indian or Native American	1 (1.0)	0
Other	1 (1.0)	4 (4.1)
Subject did not answer	1 (1.0)	0
Ethnicity, *n* (%)		
Hispanic or Latino	2 (2.1)	3 (3.1)
Not Hispanic or Latino	92 (95.8)	92 (94.8)
Unknown	2 (2.1)	1 (1.0)
Subject did not answer	0	1 (1.0)
Body mass index		
*n*	86	84
Mean	28.8	28.4
SD	6.0	5.8
AIS Grade, *n* (%)		
A	49 (51.58)	52 (53.61)
B	19 (20.00)	19 (19.59)
C	26 (27.37)	26 (26.80)
D	0	1 (1.05%)
Neurological Level of Injury, *n* (%)		
C3	0	2 (2.06)
C4	47 (48.96)	57 (58.76)
C5	29 (30.21)	20 (20.62)
C6	13 (13.54)	9 (9.28)
C7	5 (5.21)	5 (5.15)
C8	0	1 (1.03)
T2	0	1 (1.03)
Baseline Total Motor Score		
*n*	94	95
Mean	18.44	16.46
SD	13.39	12.62
Baseline Upper Motor Score		
*n*	95	95
Mean	14.02	12.63
SD	10.46	10.87
Baseline Lower Motor Scores		
*n*	94	97
Mean	4.38	3.76
SD	8.51	7.42

SD, standard deviation; AIS, American Spinal Injury Association Impairment Scale.

From the primary efficacy analysis in the complete cases cohort at 180 days, patients who had riluzole had 1.8 (95% confidence interval [CI]: -2.5-6.1) higher average gain in UEM scores when compared with placebo, which did not reach statistical significance (16.4 vs. 14.7; Table 2). Analysis of the other ISNCSCI motor end-points at 180 days revealed higher average in the riluzole patients compared with placebo in TOTM (34.0 vs. 31.1; d: 2.86 CI: -6.8-12.5) and LEM (17.6 vs. 16.1; d: 1.45 CI: -4.8-7.7) scores from baseline, which did not reach statistical significance in the complete cases cohort (Table 2).

**Table 2.** Mean, Number of Patients, and Difference in Means by Treatment Group for Motor Scores Gained at 180-Days

	Placebo		Riluzole			
	Mean	*n*	Mean	*n*	Difference (95% CI)	*p* Value
Complete Cases (*n*: 137)						
Primary Outcome: Change in Upper Extremity Motor Scores at 180 days	14.65	66	16.42	65	1.76 (-2.54-6.06)	0.2093
Change in Lower Extremity Motor Scores at 180 days	16.10	68	17.55	65	1.45 (-4.80-7.70)	0.3235
Change in Total Motor Scores at 180 days	31.11	66	34.00	65	2.86 (-6.79-12.52)	0.2792
Intention to Treat- Imputed data (N: 188)						
Primary Outcome: Change in Upper Extremity Motor Scores at 180 days	14.35	94	15.59	94	1.24 (-1.90-4.38)	0.2190
Change in Lower Extremity Motor Scores at 180 days	16.54	94	15.99	94	0.02 (-4.7-4.77)	0.4962
Change in Total Motor Scores at 180 days	29.83	90	31.25	93	1.42 (-5.78-8.62)	0.3490

The *p* values were calculated using a one-tailed t-test with the alternative hypothesis being that the difference in scores is greater in the riluzole group. Upper Extremity Motor group score change is the pre-established primary end-point.

CI, confidence interval.

**FIG. 2.**
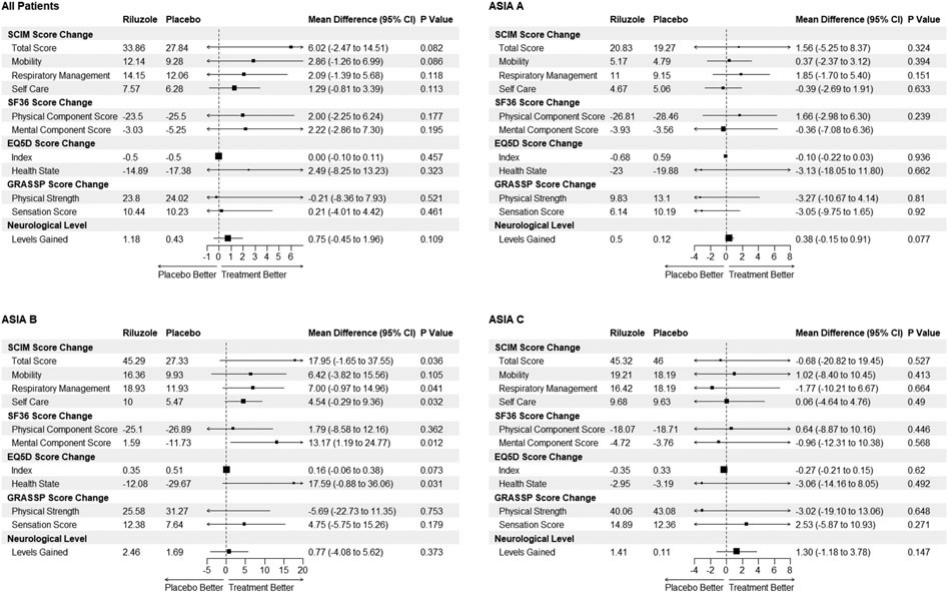
Changes in secondary and other end-points at 180 days from baseline in different baseline American Spinal Injury Association Impairment Scale subgroups in patients with traumatic cervical spinal cord injury randomized to either riluzole 100 mg oral dose twice per day (PO BID) for 24 h followed by 50 mg PO BID for 13 days after injury or placebo control. The *p* values were calculated using a one-tailed t-test with the alternative hypothesis being that the difference in scores is greater in the riluzole group. Analysis conducted on the complete cases population; *n*: 137.

Analysis of pre-specified secondary end-points (Fig. 2) revealed that patients treated with riluzole on average had a gain of 33.9 SCIM points at 180 days compared with a gain of 27.8 points in the placebo group (Fig. 2; 95% CI for change in SCIM: -2.5-14.5). At 180 days, the mean AIS grade change was comparable between the riluzole (0.98) and control (1.00) groups, with 48 patients (73.85%) who received riluzole having one or more AIS grade improvements compared with 45 patients (66.18%) who received a placebo (*p*: 0.335; Table S6 in the Supplementary Material). The descriptive statistics of change in motor scores from baseline at different time points are tabulated in Table S7 in the Supplementary Material.

The average gain was higher in the riluzole group in most of the other pre-specified end-points (Fig. 2) including neurological levels at 180 days (1.2 vs. 0.4, difference: 0.8 with 95% CI -0.5-2.0), SF36 mental (-3.0 vs. -5.2; D: 2.2 CI: -8.3-13.2) and physical component (-23.5 vs. -25.5; d: 2.0 CI:-2.3-6.2) score change at 180 days and EuroQol 5-Dimension (EQ5D) Health State change at 180 days (-14.9 vs. -17.4; d: 2.5 CI: -8.25-13.2). No statistically significant differences between the treatment groups were observed in total pinprick, sensory score (Fig. 2), or the GRASSP strength and sensation score change at 180 days.

In the AIS A population, patients in the riluzole subgroup on average had 0.50 neurological levels gained at 180 days compared with 0.12 levels in the placebo group (Fig. 2; d: 0.38, CI: -0.2-0.9). The motor score changes at 180 days from baseline for each of the AIS sub groups are displayed in Figure S1 in the Supplementary Material.

**FIG. 3.**
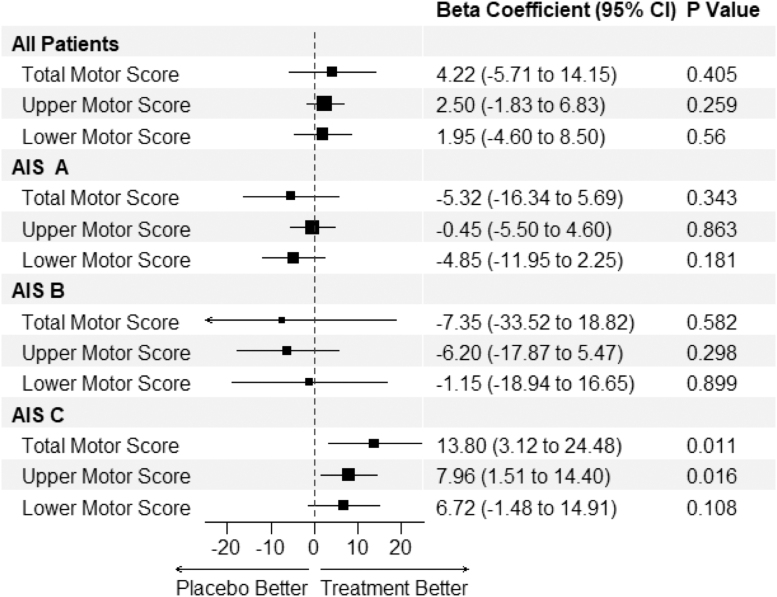
*Post hoc* multi-variate linear regression of changes in Lower and Upper Extremity and Total International Standards for Neurological Classification of Spinal Cord Injury motor scores at 180 days compared with baseline with the administration of riluzole in different baseline American Spinal Injury Association Impairment Scale subgroups in patients with traumatic cervical spinal cord injury randomized to either riluzole 100 mg oral dose twice per day (PO BID) for 24 h followed by 50 mg PO BID for 13 days after injury or placebo controlled. Estimates represent the beta coefficient. Covariates used in the model include age, Charlson comorbidity Index, gender, neurological level of injury, and race. Analysis in the complete cases cohort; *n*: 137.

In the AIS B population there were average gains in favor of riluzole with an SF-36 mental component score mean gain of 1.6 at 180 days vs. -11.6 in the placebo group (Fig. 2, d: 13.2 CI: 1.2-24.8), SCIM score gain of 45.3 vs. 27.3 (d: 18.0 CI: -1.7-38.0) and EQ5 Health Status score change of -12.1 vs. -29.7 (d: 17.6 CI: 1.2-24.8). The SCIM score improvements seen in the AIS B population were predominately in the self-care, respiration, and sphincter management domains. In the AIS C subgroup, the administration of riluzole compared with placebo was associated with an increase in Upper Motor, (standard error [SE] 8.0; CI 1.5-14.4), and Total Motor (SE 13.8; CI 3.1-24.5) score change at 180 days compared with baseline in *post hoc* multi-variate linear regression models (Fig. 3). The full results of the multi-variate model are tabulated in Table S8 in the Supplementary Material.

In the riluzole group, there were 1722 adverse events (AEs) in 96 participants and 110 serious adverse events (SAEs) in 51 participants with nine deaths (Table 3). In the placebo group, there were 1786 AEs in 97 participants with 52 SAEs in 132 participants and 10 deaths. Of the AEs in the riluzole group, 69.9% resolved (1,204) compared with 66.9% (1,195) in the placebo group. There was no withdrawal of study medication due to AEs. Adverse events by disease categories are summarized in Table S9 in the Supplementary Material. Analysis of changes in laboratory values did not reveal any statistically significant difference in elevation of liver enzymes at 14 days between Riluzole and placebo control (Table S10 in the Supplementary Material).

**Table 3.** Adverse Events in the RISCIS Study

	Riluzole (*n* = 96)			Control (*n* = 97)		
	Participants	Events	Wilson confidence interval	Participants	Events	Wilson confidence interval
Death	9 (9.78)	9	0.05-0.17	10 (9.52)	10	0.05-0.18
Serious adverse event	51 (53.1)	110	0.06-0.09	52 (53.6)	132	0.05-0.08
Any adverse event	96 (100.0)	1722		97 (100.0)	1786	
Discontinuation of study medication due to adverse event	0	0		0	0	
Outcome						
Resolved	92 (95.8)	1204		92 (94.8)	1,195	
Resolved with residual effects	37 (38.5)	77		34 (35.1)	108	
Unresolved	78 (81.3)	349		75 (77.3)	405	
Unknown	27 (28.1)	81		25 (25.8)	65	

The *p* values calculated from a chi-squared test. Analysis in the modified intent to treat population (*N*: 193).

Alternative *post hoc* approaches to the primary efficacy analysis, including analysis of covariance models and binary outcome analyses, were conducted and presented in Figure S2 and Table S11 in the Supplementary Material, respectively. These approaches, used as secondary sensitivity analyses, did not change the primary conclusions summarized above.

## Discussion

The RISCIS trial was terminated prematurely by the sponsor in the face of the COVID-19 pandemic. The burden of the pandemic was not unique to this trial; it is estimated that 2043 clinical trials were terminated or suspended as a result of the pandemic, impacting 4 million future participants.^23^ There was no evidence that riluzole was associated with drug-related serious adverse events.

Analysis of the primary end-points revealed an increased average gain in UEM scores at 180 days in the riluzole group compared with placebo, which did not reach the criteria for statistical significance. Increased recovery seen with riluzole was replicated in other end-points including all the ISNCSCI motor end-points as well as additional end-points such as neurological levels gained, SCIM change, SF-36 score change and EQ5D Health Status change from baseline. The 18-point difference in total SCIM score gained seen with riluzole patients in the AIS B subgroup (*n*: 29) is above the 10-point cutoff considered to represent substantial clinical improvement.^24^ While the improvement in the SCIM score in riluzole treated patients could be partially attributed to a greater proportion of patients with higher-level injuries in the control group, resulting in disproportionate changes in respiratory management scores, a significant change in self-care scores was also observed among patients treated with riluzole.

Pre-planned subgroup analyses revealed neurological and functional improvements with riluzole in each AIS grade subgroup. In the AIS grade A population, treatment with riluzole had a higher mean increase in neurological levels gained compared with placebo. In the AIS B sub-population, patients treated with riluzole had a statistically superior SCIM, EQ5D health status change, and SF-36 mental score change at 180 days compared with baseline. In the AIS grade C subgroup, the administration of riluzole compared with placebo was associated with a statistically significant increase in Upper Motor score, and Total Motor score change at 180 days compared with baseline in multi-variate models. There was, however, no significant change in the total SCIM score in the AIS C population.

The motor score and functional improvements observed in the riluzole group tended to be greater in the incomplete (AIS grades B and C) cervical tSCI cohort. It can be hypothesized that the limited regenerative capacity seen with a severe AIS A tSCI limits the utility of neuroprotective agents in facilitating recovery. Alternatively, it can be argued that motor and functional scores are not the most sensitive measures of improvement in the AIS A population. Subgroup analysis of motor levels gained in the AIS A population showed greater neurological level improvements in the riluzole group at 180 days from baseline (0.50 vs. 0.12 in placebo; *p*: 0.077).

The investigation of the pharmacodynamics of riluzole^25^ has yielded valuable insights into the efficacy of this drug in the context of tSCI. Administration of riluzole was found to be associated with a reduction in phosphorylated neurofilament heavy chain (pNF-H), a biomarker indicative of axonal degradation, when compared with placebo. In addition, three-dimensional pharmacokinetic/pharmacodynamic models revealed a positive correlation between riluzole concentration and neurological recovery after 6 months.

The outcomes of this trial underscore the necessity of establishing a minimally clinically important difference (MCID) in tSCI that can be employed as a study end-point in clinical trials. During the development of this trial, a change of 9 total motor score was chosen as a suitable end-point for power calculations. Over the course of the past several years, and since the initiation of the RISCIS trial, the actual MCID is thought to be much lower^26^ and more closely aligned with the changes observed in this trial. Some believe that any neurological change constitutes an MCID, while others have utilized distribution-based analyses to estimate a MCID of 5 in total motor scores.^27^

It might be advisable to redirect the emphasis towards integrating functional outcomes, such as SCIM, into the primary analyses of clinical trials. Patients may observe substantial enhancements in their quality of life due to functional improvements that might not be captured in their overall motor scores. Therefore, the influence of neuroprotective and regenerative agents on functional recovery and the augmentation of rehabilitation efforts warrants further investigation.

Given the limitations of a neurological exam in the acute stages of tSCI, and the challenges in defining a broadly applicable MCID, objective imaging and chemical biomarkers such as pNF-H may offer more accurate indicators of neurological recovery. In this regard, further research aimed at validating biomarkers of neurological recovery in tSCI would be advantageous for future trials of neuroprotective agents.

The analysis of the trial results has limitations as only 55% of the pre-planned sample size was recruited. Further, the study cohort is predominately composed of high severity injuries with AIS A and upper cervical injuries, which tend to have diminished recovery potential. Given that the trial did not reach its recruitment targets, the conclusion of this trial concerning the pre-specified superiority criteria needs to be interpreted in context. However, the pre-planned primary analyses, subgroup analyses, and *post hoc* analyses reveal encouraging outcomes with regard to potential neurological and functional recovery with the use of Riluzole in acute cervical tSCI patients.

As further trials of this magnitude will be challenging to undertake due to the significant challenges associated with conducting clinical trials in tSCI, the results of the RISCIS trial could stimulate review by guidelines committees to better delineate the potential role of riluzole in the management of tSCI. From a risk-benefit perspective, the use of riluzole appears to have an excellent safety profile in tSCI patients. While this study cannot make definitive statements on the efficacy of riluzole, there are clinically interesting effects seen in neurological and functional recovery across different outcome measures and subgroups. Given the lack of alternative pharmacological treatments for severe tSCI, there may be a lower clinical threshold for accepting a higher false positive probability with respect to potentially facilitating neurological recovery. This is particularly relevant given that tSCI is an uncommon, orphan disorder without an effective, accepted neuroprotective or neuroregenerative treatment.

The time-sensitive, heterogeneous, and relatively uncommon nature of SCI presents significant challenges that warrant innovative approaches to clinical trials. Similar challenges exist in other neurological disorders, leading to high failure rates in clinical trials targeting these conditions.^28^ In response, advocacy communities have lobbied for revised regulatory guidance on clinical trials for Duchenne muscular dystrophy^29^ and ALS,^30^ with recommendations such as virtual controls, increased use of secondary outcomes, and potential elimination of the placebo arm.

Apart from regulatory changes, a more significant focus on personalized, patient-centered outcomes and trial design could further enhance the utility of clinical trials. Multi-arm, multi-stage (MAMS) trial platforms, successfully employed in rare disease and oncology research, can also be adapted to tSCI research to reduce trial costs and patient recruitment burdens. MAMS trials have been proposed for Parkinson's disease^31^ and ALS research (ClincalTrials.gov NCT04302870), demonstrating their potential applicability in tSCI research.

## Transparency, Rigor, and Reproducibility Summary

The study design and analysis plan were pre-registered on May 14th, 2012 at ClinicalTrials.gov (NCT01597518). Pre-specified sample size was 156 per group, yielding statistical power of 0.90 for detection of an effect size of 9 for total motor score change. All subjects were assigned to riluzole or placebo using a block randomization yielding groups that did not differ in baseline characteristics. A total of 96 subjects were engaged with the study medication, and primary outcomes were assessed in 68 subjects after nine deaths and 28 incomplete assessments. All primary outcomes were assessed by investigators blinded to group assignment and could guess the group assignment with accuracy no greater than chance. All materials required to perform the interventions are widely available. The primary outcome and the key inclusion criteria are established standards in the field of tSCI. With the adaptive trial design during the interim analysis, the original sequential method was switched to a non-binding futility hypothesis with appropriate adjustments to the superiority and futility thresholds. The findings have not yet been replicated or externally validated. Individual participant data that underlines the results reported in this article, after de-identification (text, tables, figures, and appendices), will be made available alongside the study protocol by contacting the corresponding author.

## Data Sharing

For sharing purposes, reuse conditions will be respected. Individual participant data that underlie the results reported in this article, after de-identification (text, tables, figures, and appendices), will be made available along side the study protocol. The data would be made available for individual participant data meta-analysis. Proposals may be submitted up to 36 months following article publication. Proposals should be directed to michael.fehlings@uhn.ca to gain access. Proposals will be reviewed by a committee of the RISCIS investigators. Data requestors will need to sign a data access agreement.

## Supplementary Material

Supplemental data

Supplemental data

Supplemental data

Supplemental data

Supplemental data

Supplemental data

Supplemental data

Supplemental data

Supplemental data

## Supplementary Material

RISCIS Protocol

RISCIS Statistical Analysis Plan

Interim Analysis

CONSORT Adaptive Design

CONSORT Adaptive Design Abstract

CONSERVE Checklists

CONSORT Harms

Data Safety and Monitoring Board Charter

Data Safety and Monitoring Board Recommendations
