# Supplementary material for: Safety and Efficacy of Riluzole in Acute Spinal Cord Injury Study (RISCIS): A Multi-Center, Randomized, Placebo-Controlled, Double-Blinded Trial
Source: J Neurotrauma. 2023 Aug 23;40(17-18):1878–88. doi: 10.1089/neu.2023.0163 (PMC10460693; doi:10.1089/neu.2023.0163)
Supplement: Supplemental data [file 3RISCISPROTOCOL.pdf]

A Multi-Center, Randomized, Placebo-Controlled, Double-Blinded, Trial of Efficacy and Safety of Riluzole in Acute Spinal Cord Injury

Reference Number: SPN-12-001

CONFIDENTIAL INFORMATION

The information provided in this document is confidential and is intended for the use of clinical investigators. It is the property of AOSpine North America and should not be copied by or distributed to persons not involved in the clinical investigation described in this protocol, unless such persons are bound by a confidentiality agreement with AOSpine North America.

Version 1.1 September 5, 2013

Protocol Approved: September 5, 2013

COLLABORATORS

AOSpine North America
AOSpine International
Christopher Reeve Foundation*
Ontario Neurotrauma Foundation*

** Finalization of the collaborations with Christopher Reeve Foundation and Ontario Neurotrauma Foundation is pending completion of the formal agreements. Other collaborative partners may join the study.*

Acknowledgment

This protocol has been developed by the AOSpine North America Research Network team led by Dr. Michael Fehlings with input from the AOSpine North America Methodology Core led by Dr. Branko Kopjar and the North American Clinical Trials Network (NACTN) led by Dr. Robert Grossman.

Copyright 2012 2013 by AOSpine North America. All rights reserved. This material may not be duplicated, in part or in whole without written permission by AOSpine North America. Requests for copies, duplication and use of materials, in part or in whole should be addressed to AOSpine North America.

1 Protocol Approval....7
2 Investigator Statement....8
3 Abbreviations....9
4 Synopsis....11
5 Background Information....18
5.1 Justification for Investigation....18
5.1.1 Spinal Cord Injury Epidemiology and Pathophysiology....18
5.1.2 Treatment of Spinal Cord Injury....18
5.2 Riluzole....18
5.2.1 Molecular Structure....18
5.2.2 Pharmacology and Pharmacokinetics of Riluzole....19
5.2.3 Riluzole in ALS....19
5.2.4 Riluzole in Other Diseases....20
5.2.5 Riluzole in Animal Models of SCI....20
5.2.6 Pharmacokinetics of Riluzole in SCI Patients....20
5.2.7 Regulatory Status....22
5.2.8 Investigational Drug....23
6 Study Design....24
6.1 Study Scope....24
6.2 Study Objectives....24
6.3 Endpoints....24
6.3.1 Primary Efficacy Endpoint....24
6.3.2 Secondary Efficacy Endpoints....24
6.3.3 Other Endpoints....24
6.3.4 Safety Evaluation....25
6.4 Selection of Investigators and Investigational Sites....25
6.5 Inclusion Criteria....25
6.6 Exclusion Criteria....25
6.7 Study Procedures....26
6.7.1 Screening and Enrollment....26
6.7.2 Investigational Drug Dose and Administration....27
6.7.3 Steroid Administration....29

6.7.4 Blinding ... 29
6.7.5 Enrollment ... 29
6.7.6 Follow-up Evaluations ... 30
6.7.7 Study Schematic ... 30
6.7.8 Medical and Surgical Care ... 37
6.7.9 Post-surgical Rehabilitation and Occupational Therapy ... 38
6.7.10 Clinical Laboratory Procedures ... 38
6.8 Subjects Completion and Disposition ... 39
6.8.1 Screen Failure ... 39
6.8.2 Enrolled Subject ... 40
6.8.3 Withdrawn (Discontinued) Subject ... 40
6.8.4 Lost to Follow-up ... 40
6.8.5 Completed Subject ... 40
6.9 Study Site Discontinuation ... 41
6.10 Study Discontinuation ... 41
7 Statistical Analysis ... 42
7.1 Primary Efficacy Endpoint ... 42
7.2 Secondary Efficacy Endpoints ... 42
7.3 Other Endpoints ... 42
7.4 Adverse Events ... 42
7.5 Study Success ... 42
7.6 Statistical Testing of Primary Endpoint ... 42
7.7 Statistical Testing of Secondary Endpoints ... 43
7.8 Safety Evaluations ... 43
7.9 Sample Size Estimate ... 43
7.10 Justification for Effect Estimate ... 44
7.11 Statistical Performance and Simulations ... 44
7.12 Adaptive Design and Sample Size Re-estimate ... 46
7.13 Analysis Populations ... 46
7.13.1 Intention-to-Treat ... 46
7.13.2 Modified Intention-to-Treat ... 47
7.13.3 Completed Cases Population ... 47

7.13.4 Per-protocol Population ... 47
7.13.5 Primary Analysis Populations ... 47
7.14 Randomization ... 47
7.15 Evaluation of 365 days follow-up data ... 47
7.16 Pre-planned Subgroup Analyses ... 48
8 Risk Analysis ... 49
8.1 Risks ... 49
8.2 Actions to Minimize Increased Risks ... 50
9 Adverse Event Reporting ... 51
9.1 AE Definitions ... 51
9.1.1 Adverse Events ... 51
9.1.2 Suspected Adverse Reaction ... 51
9.1.3 Unexpected Adverse Event/Reaction ... 52
9.1.4 Serious (or Life-threatening) Adverse Events or Adverse Reaction ... 52
10 Investigator Responsibilities ... 53
10.1 Investigator Qualifications ... 53
10.2 IRB/REB/ERC Ethical Approval ... 53
10.3 Protocol Adherence ... 53
10.4 Review of Source Documents ... 53
10.5 Record of Investigational Drug Inventory ... 53
10.6 Data Recording and Record Retention ... 54
10.7 Notification Reporting ... 54
11 Study Data Reporting and Processing ... 55
11.1 Study Data Collection ... 55
11.2 Site Data Monitoring and Quality Control ... 55
11.3 Subject Coding ... 56
11.4 Data Processing and Quality Control ... 56
11.4.1 Electronic Data Capture System ... 56
11.4.2 Data Cleaning ... 56
11.4.3 Data Entry ... 56
11.4.4 Data Editing ... 56
11.4.5 Data Update ... 57

11.4.6 Final Data Quality Analyses 57
11.4.7 Data Back-up 57
11.4.8 Report Generation and Summary Statistics 57
11.5 Confidentiality and Protection of Study Files 57
12 Study Management 58
12.1 Operations Committee........ 58
12.2 AOSpine North America Research Network Methods Core 58
12.3 Safety Officer 58
12.4 Data Safety and Monitoring Board (DSMB) 58
12.5 Monitoring........ 58
12.5.1 Pre-Investigation Visits (Site Evaluation Visit)........ 58
12.5.2 Initiation Visit 59
12.5.3 Periodic Visits 59
12.5.4 Close-out Visit 59
12.5.5 Record of On-Site Visits 60
12.5.6 Direct Access to Source Documentation 60
13 Document Control 61
13.1 Responsibility........ 61
13.2 Protocol Amendments 61
13.3 Protocol Deviations 61
13.4 Protocol Violations........ 61
13.5 Record Retention........ 61
14 Appendices 63
14.1 Appendix A: Riluzole Drug Monograph........ 63
14.2 Appendix B: Scales and Questionnaires 63
14.3 Site and Topic Specific Protocol Addendums 63
15 References 64

1 Protocol Approval

Clinical Investigation Plan

A Multi-Center, Randomized, Placebo-Controlled, Double-Blinded, Trial of Efficacy and Safety of Riluzole in Acute Spinal Cord Injury (SPN-12-001)

Sponsor

AOSpine North America
1700 Russell Road
Paoli, PA 19301

Authorized Representative (Signatory)

[Signature on file with AOSNA] ____________________ September 5, 2013
Michael G. Fehlings MD, PhD, FRCSC, FACS Date
Principal Investigator

[Signature on file with AOSNA] ____________________ September 5, 2013
Nancy Holmes, Director Date
AOSpine North America

Reference: 05Sep2013, Version 1.1

2 Investigator Statement

SPONSOR:

AOSpine North America

A Multi-Center, Randomized, Placebo-Controlled, Double-Blinded, Trial of Efficacy and Safety of Riluzole in Acute Spinal Cord Injury

Protocol Number: SPN-12-001

Version 1.1: September 5, 2013

I, the Investigator of Record, agree to conduct this study in full accordance with the provisions of this Protocol, associate site agreement and in accordance with applicable regulations and conditions required by the trial overseeing authorities. I agree to maintain all study documentation for a minimum of two years after the study has been completed or for longer periods as required by law and regulations. Publication of the results of this study will be governed by the conditions stipulated in the Site Agreement. I agree to supervise use of the Investigational Drug at my institution and ensure that the informed consent document is obtained prior to subject enrollment.

I have read and understand the information in this Protocol and will ensure that all associates, colleagues and employees assisting in the conduct of the study are informed of the obligations incurred by their participation. I will attest to the delegation of any obligation under this Protocol to my associates, colleagues and employees by signing such delegation in the Study Delegation Log.

____

Name of Site Principal Investigator

___________________________________ ___________________________________

Signature of Site Principal Investigator Date

3 Abbreviations

**β-hCG**	β-human Chorionic Gonadotropin
**AE**	Adverse Event
**AIDS**	Acquired immune deficiency syndrome
**ALP**	Alkaline Phosphatase
**ALS**	Amyotrophic Lateral Sclerosis
**ALT**	Serum Alanine Transaminase
**ANC**	Absolute Neutrophil Count
**AOSI**	AOSpine International
**AOSNA**	AOSpine North America
**BID**	Bis In Die (Twice Daily)
**CC**	Completed Cases
**CRO**	Contract Research Organization
**DSMB**	Data Safety and Monitoring Board
**eCRF**	Electronic Case Report Form
**EDC**	Electronic Data Capture
**ERC**	Ethics Review Committee
**FDA**	Food and Drug Administration
**GCP**	Good Clinical Practice
**GGT**	Gamma-Glutamyl Transpeptidase
**GRASSP**	Graded Redefined Assessment of Strength Sensibility and Prehension
**ICD**	Informed Consent Document
**IND**	Investigational New Drug

**INR**	International Normalized Ratio
**IRB**	Institutional Review Board
**ISNCSCI**	International Standards for Classification of Spinal Cord Injury
**ISNCSCIMS**	ISNCSCI Motor Score
**ITT**	Intent-To-Treat
**mITT**	Modified Intent-To-Treat
**MPSS**	Methylprednisolone Sodium Succinate
**NACTN**	North American Clinical Trials Network
**Pain NRS**	Numeric Pain Rating Scale
**REB**	Review Ethical Board
**SAE**	Serious Adverse Event
**SAP**	Statistical Analysis Plan
**SCI**	Spinal Cord Injury
**SCIM**	Spinal Cord Independence Measure
**SF-36v2™**	Short Form 36 Version 2
**SIC**	Subject Investigational Code
**SMA**	Spinal Muscular Atrophy
**SOP**	Standard Operating Procedure
**SW**	Source Worksheet
**TEAE**	Treatment-Emergent Adverse Event
**UAE**	Unanticipated Adverse Event
**ULN**	Upper Limit of Normal

4 Synopsis

**Official Title**	A Multi-Center, Randomized, Placebo-Controlled, Double-Blinded, Trial of Efficacy and Safety of Riluzole in Acute Spinal Cord Injury
**Short Title**	Riluzole in Spinal Cord Injury Study (RISCIS)
**Sponsor**	AOSpine North America<br>Note: Organizations listed in the footnote below are anticipated to join as co-sponsors[1]
**Background and Purpose**	At present there are over 1 million people living with Spinal Cord Injury (SCI) in North America alone, with annual costs for the acute treatment and chronic care of these patients totaling four billion dollars USD. The worldwide prevalence of SCI is unknown, with estimates ranging up to 250 million individuals. The incidence of SCI in developed countries has been estimated to be between 10 – 40 cases per million inhabitants. In spite of the immense impact of SCI at a personal and societal level, an effective and safe pharmacologic treatment for SCI, shown to improve neurological and functional outcomes at long-term follow-up, remains absent.<br>The final degree of neurological tissue destruction that occurs after traumatic SCI is a product of both primary and secondary injury mechanisms. The primary mechanical injury to the cord initiates a subsequent signaling cascade of deleterious down-stream events, known collectively as secondary injury mechanisms. These secondary injury mechanisms include ischemia, interstitial and cellular ionic imbalance, free radical formation, glutamatergic excitotoxicity, lipid peroxidation and generation of arachidonic acid metabolites. Although little can be done from a therapeutic standpoint to correct damage sustained during the primary injury, by mitigating the evolution of secondary injury events there is opportunity to preserve remnant viable neurological tissue and improve neurologic outcomes.<br>There is convincing evidence from the preclinical realm that the pharmacologic agent riluzole attenuates certain aspects of the secondary injury cascade leading to diminished neurological tissue destruction in animal SCI models. Riluzole, a sodium channel blocking benzothiazole anticonvulsant, specifically exerts its neuroprotective effect by helping to maintain neuronal cellular ionic balance and by reducing the release of excitotoxic glutamate in the post-SCI setting. Several preclinical studies in the rodent SCI model have associated administration of riluzole with increased neural tissue preservation at the site of injury, in addition to improved behavioral outcomes, in comparison to administration of placebo or other sodium channel blocking drugs. In the clinical realm, while riluzole has not been

[1] AOSpine International, Christopher Reeve Foundation and Rick Hansen Institute

	studied extensively in the context of SCI, it has been widely used in the treatment of amyotrophic lateral sclerosis (ALS). A 2007 Cochrane review, summarizing the findings of 4 placebo-controlled randomized trials, concluded that at a dose of 100 mg daily, riluzole is safe and improves median survival by 2-3 months in patients with ALS. In regards to adverse events (AEs), riluzole was well tolerated, although treated patients were 2.6 times more likely to experience a three-fold increase in serum alanine transaminase (ALT) as compared to patients treated with placebo. However, this effect was found to be uniformly reversible with cessation of riluzole therapy and was only reported after several months of medication administration. Recently, the clinical safety and pharmacokinetic profile of riluzole have been studied in a multi-center pilot study in the context of traumatic SCI. A total of 36 patients received an oral dose of riluzole 50 mg twice daily for 2 weeks, with treatment initiated within 12 hours of injury for all patients. The 12 hour dosing window, as well as the 2 week duration of therapy, was chosen to match the period of medication administration to the known period of glutamatergic excitotoxicity after SCI (several minutes after injury until 2 weeks after injury). With the final analysis currently undergoing peer review, completion of this study has confirmed the acceptable safety profile of riluzole administration previously documented in the ALS literature, and has established the feasibility of conducting a large-scale efficacy trial investigating this therapy.<br>At present, there is no specific pharmacological therapy that is given uniformly to all patients with traumatic SCI. As a result, a placebo-controlled comparison group is ethical and justifiable.
**Aim**	The aim of this study is to evaluate efficacy and safety of riluzole in the treatment of patients with acute SCI. The primary objective is to evaluate the superiority of riluzole, at a dose of 100 mg Bis In Die (Twice Daily) (BID) the first 24 hours followed by 50 mg Bis In Die (Twice Daily) (BID) for the following 13 days after injury, as compared to placebo, in change between 180 days and baseline in motor outcomes as measured by International Standards for Neurological Classification of Spinal Cord Injury Examination (ISNCSCI) Motor Score, in patients with acute traumatic SCI, presenting to the hospital less than 12 hours after injury. Secondary objectives are to evaluate the effects of riluzole on overall neurologic recovery, sensory recovery, functional outcomes, quality of life outcomes, health utilities, mortality, and adverse events. The working hypothesis is that the riluzole treated subjects will experience superior motor, sensory, functional, and quality of life outcomes as compared to those receiving placebo, with an acceptable safety profile.
**Condition**	Acute (≤ 12 hours old) traumatic SCI
**Intervention**	Drug: 1. Experimental: riluzole 100 mg BID first 24 hours followed by 50 mg BID for 13 days<br>2. Control: placebo

**Phase**	II/III
**Study Type**	Interventional
**Study Design**	Allocation: Randomized, stratified by site<br>Endpoint classification: Efficacy/Safety<br>Model: Parallel assignment<br>Masking: Double-blinded<br>Primary purpose: Treatment
**Principal Investigator**	Michael G. Fehlings MD, PhD, FRCSC, FACS<br>Chairman, AOSpine North America Research Network<br>Professor of Neurosurgery<br>Director Neuroscience Program<br>University of Toronto<br>399 Bathurst St Suite 4WW-449<br>Toronto Ontario M5T 2S8<br>Tel +1 416 603 5627<br>Fax +1 416 603 5298<br>Email michael.fehlings@uhn.on.ca
**Co-PI**	Robert G. Grossman, MD<br>Chairman, Department of Neurosurgery<br>The Methodist Hospital<br>Professor of Neurological Surgery<br>Weill College of Medicine Cornell University<br>Chair, North American Clinical Trials Network (NACTN)<br>6560 Fannin, Suite 944<br>Houston, Texas 77030<br>Tel +1 713 441 3810<br>Fax +1 713 793 1004<br>Email rgrossman@tmhs.org
**Primary Efficacy Endpoint**	• Change in ISNCSCI Total Motor Score between 180 days and baseline
**Secondary Efficacy Endpoints**	• Change in ISNCSCI grade between baseline and 180 days<br>• Spinal Cord Independence Measure (SCIM) at 180 days
**Other Endpoints**	• Change in ISNCSCI Sensory Scores (Light Touch and Pin Prick) between 180 days and baseline<br>• Change in ISNCSCI Upper Extremity Motor Score between 180 days and baseline<br>• Change in ISNCSCI Lower Extremity Motor Score between 180 days and baseline<br>• Change in Short Form 36 Version 2 (SF-36v2™) PCS, MCS and 8 dimensions between 180 days and pre-injury (recall)<br>• Change in EQ-5D health utility between 180 days and pre-injury (recall)

	• Graded Redefined Assessment of Strength Sensibility and Prehension (GRASSP) at 14 days or Discharge (whichever occurs first) and 180 days<br>• Change in Numeric Pain Rating Scale (Pain NRS) at 14 days, 84 days and 180 days
**Safety Evaluation**	• Comparison of rate of adverse events by body system and preferred term between the riluzole and placebo arms
**Follow-up**	• Surgery (if applicable); 72 ± 12 hours post-injury; 7 ± 1 day; 14 + 2 days after enrollment; discharge from acute care; 84 days ±14 days; 180 days ± 30 days; 365 days ± 45 days
**Estimated Enrollment**	Total of 351 subjects (includes 10% attrition) randomized 1:1 to riluzole and placebo arms
**Study Start Date**	January 1, 2013
**Estimated Study Completion**	30 months duration (24 months to enroll and 6 months to follow-up)
**Arms**	Investigational: riluzole<br>Control: placebo
**Eligibility Inclusion Criteria**	• Age between 18 and 75 years inclusive<br>• Able to cooperate in the completion of a standardized neurological examination by ISNCSCI standards (includes patients who are on a ventilator)<br>• Willing and able to comply with the study Protocol<br>• Signed Informed Consent Document (ICD) by patient, legal representative or witness<br>• Able to receive the Investigational Drug within 12 hours of injury<br>• ISNCSCI Impairment Scale Grade "A," "B" or "C" based upon first ISNCSCI evaluation after arrival to the hospital<br>• Neurological Level of Injury between C4-C8 based upon first ISNCSCI evaluation after arrival to the hospital<br>• Women of childbearing potential must have a negative serum β-human chorionic gonadotropin (β-hCG) pregnancy test or a negative urine pregnancy test
**Eligibility Exclusion Criteria**	• Injury arising from penetrating mechanism<br>• Significant concomitant head injury defined by a Glasgow Coma Scale score < 14 with a clinically significant abnormality on a head CT (head CT required only for patients suspected to have a brain injury at the discretion of the investigator)<br>• Pre-existent neurologic or mental disorder which would preclude accurate evaluation and follow-up (i.e. Alzheimer's disease, Parkinson's disease, unstable psychiatric disorder with

hallucinations and/or delusions or schizophrenia)
- Previous history of spinal cord injury
- Recent history (less than 1 year) of chemical substance dependency or significant psychosocial disturbance that may impact the outcome or study participation, in the opinion of the investigator
- Is a prisoner
- Participation in a clinical trial of another Investigational Drug or Investigational Device within the past 30 days
- Hypersensitivity to riluzole or any of its components
- Neutropenia measured as absolute neutrophil count (ANC) measured in cells per microliter of blood of < 1500 at screening visit
- Creatinine level of > 1.2 milligrams (mg) per deciliter (dL) in males or > 1.1 mg per dL in females at screening visit
- Liver enzymes (ALT/SGPT or AST/SGOT) 3 times the upper limit of normal (ULN) at screening visit
- Active liver disease or clinical jaundice
- Subject is currently using, and will continue to use for the next 14 days any of the following medications which are classified as CYP1A2 inhibitors or inducers*:
  Inhibitors:
  - Ciprofloxacin
  - Enoxacin
  - Fluvoxamine
  - Methoxsalen
  - Mexiletine
  - Oral contraceptives
  - Phenylpropanolamine
  - Thiabendazole
  - Zileuton

  Inducers:
  - Montelukast
  - Phenytoin

*Note: no washout period required; if these medications are discontinued, subjects are eligible to be enrolled in the trial

- Acquired immune deficiency syndrome (AIDS) or AIDS-related complex
- Active malignancy or history of invasive malignancy within the last five years, with the exception of superficial basal cell carcinoma or squamous cell carcinoma of the skin that has been definitely treated. Patients with carcinoma in situ of the uterine cervix treated definitely more than 1 year prior to enrollment may enter the study
- Lactating at screening visit

**Investigational Sites**	Up to 35 sites
**Statistical Plan**	The statistical analysis will test the null hypothesis of the superiority of riluzole compared to placebo in change of ISNCSCI Motor Score between the baseline and the 180-day follow-up (Δ ISNCSCIMS).<br><br>Statistical Test. The statistical testing of H_0 for the primary endpoint will be organized as a 2-stage sequential adaptive design. There will be one interim analysis at about 60% of the accrued sample and the final analysis. The overall sequential design will be organized in the following way. The statistical design will address efficacy and futility. The statistical testing of H_0 hypothesis will be performed as a one-way test with alpha level .025, testing the superiority of riluzole arm compared to placebo arm. The superiority of placebo over riluzole (i.e. harmful effect of riluzole) will not be tested as it has no clinical implication. Alpha-spending for the testing of H_0 will resemble an O'Brien-Fleming distribution. The testing for futility (H_1) will consequently be organized as one-way testing. The beta spending for futility testing will follow γ -distribution with the parameter (-1). The results of the interim analysis will be reviewed by the DSMB and will not be shared with the sponsor, participating investigators or patients, except in the case that the study reaches stopping criteria.<br><br>Sample Size. Based on the above statistical design specifications and empirically-derived standard deviation for ISNCSCIMS change of 24.08 from a large case series of prospectively followed SCI subjects in an earlier study, a sample size of 316 subjects (158 in each arm) will have 90% power to detect 9 points difference in the Δ ISNCSCI Motor Score at one-sided $\alpha = .025$. To account for losses to follow-up of up to 10%, the study will enroll 351 subjects.<br><br>The sample size estimate is based on certain assumptions. The main assumptions affecting the sample size is that of the true effect size and the standard deviation for the difference in the Δ ISNCSCIMS. These assumptions will be verified during the study and sample size adjustment will be performed if needed, using the adaptive techniques. The sample size adjustment will be performed after the first interim analysis of the data, currently planned at about 60% of the data available at 180 days follow-up. The details of the adaptive design will be provided in a separate Statistical Analysis Plan (SAP).<br><br>Study success. Study will be considered to successfully confirm the working hypothesis if H_0 for the primary endpoint has been rejected either at interim or the final analysis.<br><br>Secondary outcomes. Testing for all secondary outcomes will be based on appropriate statistical methods and two-way superiority testing. Secondary outcomes will not be tested at the interim analysis, except if

	the stopping rules were met.<br><br>Preplanned subgroup analysis. A pre-planned subgroup analysis will compare differences in Δ ISNCSCIMS among the subjects with ISNCSCI Impairment Scale Grade "A," "B" and "C." Other pre-planned subgroup analyses can be performed if pre-specified in the Statistical Analysis Plan.<br><br>Safety. Safety will be monitored through the course of the study by a designated Safety Officer. Trends in serious adverse events (SAEs), laboratory events and treatment-emergent adverse events (TEAEs) will be reviewed by external Data Safety and Monitoring Board (DSMB). The DSMB will evaluate safety information against the pre-specified safety stopping rules.
**Health Authority**	USA: Institutional Review Boards (IRB) at participating sites<br>Canada: Health Canada<br>Europe: Competent authorities and Ethics Committees in respective countries<br>Rest of the world: Ethics Committees at participating sites

5 Background Information

5.1 *Justification for Investigation*

5.1.1 *Spinal Cord Injury Epidemiology and Pathophysiology*

The annual incidence of traumatic spinal cord injury (SCI) varies depending on the region considered, with international estimates ranging from 10 to 85 per million populations [1-3]. At present there are over one million people living with SCI in North America, with annual costs for the acute treatment and chronic care of these patients totaling 4 billion dollars USD [4, 5]. In spite of the immense impact of SCI at a personal and societal level, a highly effective and safe pharmacologic treatment for SCI, shown to improve neurological and functional outcomes, remains absent [6].

The final degree of neurological tissue destruction that occurs after traumatic SCI is a product of both primary and secondary injury mechanisms [7-9]. The primary mechanical injury to the cord initiates a post-lesion signaling cascade of deleterious down-stream events, known collectively as secondary injury mechanisms. These secondary injury mechanisms include ischemia, interstitial and cellular ionic imbalance, free radical formation, glutamatergic excitotoxicity, lipid peroxidation and generation of arachidonic acid metabolites [10-13]. Although little can be done from a therapeutic standpoint to correct damage sustained during the primary injury, by mitigating the evolution of secondary injury events, there is opportunity to preserve remnant viable neurological tissue and hence optimize outcomes [14].

5.1.2 *Treatment of Spinal Cord Injury*

Clinical guidelines for the management of SCI have been established and are widely accepted by physicians who treat patients with SCI [15]. These guidelines include restoration of spinal stability, decompression of the spinal cord and cardiopulmonary and metabolic support of the patient. However, beyond supportive care, there are no medical or surgical treatments that have been clearly demonstrated to improve functional outcome in human SCI. Clinical trials with methylprednisolone (NASCIS II and III) [16] [17] and GM-1 ganglioside [18] have provided suggestive but unequivocal evidence of benefit. A recent prospective, multi-center study has suggested that early decompression, within the first 24 hours after injury, is associated with better neurological outcomes than late surgery [19].

At present, there is no specific pharmacological therapy that is given uniformly to all patients with traumatic SCI. Hence, it is ethically justifiable to use placebo as control in the trial of riluzole in the context of SCI.

5.2 *Riluzole*

5.2.1 *Molecular Structure*

Riluzole is a capsule-shaped, white, film-coated tablet for oral administration containing 50 mg of riluzole. Chemically it is 2-amino-6-(trifluoromethoxy) benzothiazole. Its molecular formula is $C_8H_5F_3N_2OS$ and its molecular weight is 234.2. Its structural formula is as follows:

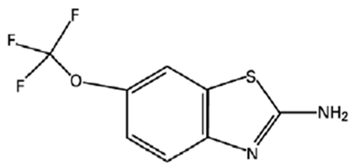

5.2.2 Pharmacology and Pharmacokinetics of Riluzole

In human studies, riluzole has been administered intravenously in a single dose of 50 mg and orally in a variety of dose combinations ranging from single doses of 25 mg, 50 mg and 100 mg, incremental doses up to 300 mg and in repeated doses of 25 mg, 50 mg and 100 mg BID [20]. Riluzole is rapidly absorbed from the gastrointestinal tract and reaches maximum plasma concentration (C_{max}) within approximately 1 hour after oral administration [20, 21]. Studies show that when administered at a dose of 50 mg BID, the C_{max} is achieved, on average, at 0.75 - 0.9 hours (SD = 0.5 hours) [20, 21]. After reaching the C_{max}, the plasma concentration rapidly declines [20-22]. The steady state through plasma concentrations are achieved within five days [20]. At a dosage of 50 mg BID, the terminal elimination half-life was 14.7 hours [20]. Several studies have reported that men have lower peak and trough levels of riluzole compared to women [21, 23]. Riluzole is highly bound to plasma proteins (96%) and is metabolized in the liver by an enzyme of the cytochrome P450 (CYP) family; specifically by a member of the CYP 1A2 subfamily, whose substrates include acetaminophen, caffeine and warfarin.

A pivotal, double-blind, randomized, controlled trial of riluzole in patients with amyotrophic lateral sclerosis (ALS) has investigated efficacy and safety of riluzole in doses of 25 mg BID, 50 mg BID and 100 mg BID [24]. The dose of 25 mg BID was not significantly better than placebo. Doses of 50 mg BID and 100 mg BID were superior to placebo, with no difference between the two doses. The dose 50 mg BID had the best benefit/harm ratio.

5.2.3 Riluzole in ALS

In the clinical realm, while riluzole has not been studied extensively in the context of SCI, it has been widely used in the treatment of ALS [24-27]. Riluzole administration has been shown in two randomized controlled trials to increase tracheostomy-free survival in patients with ALS, a progressive neurodegenerative disorder characterized by motoneuron and corticospinal tract degeneration [24, 25, 28]. A 2007 Cochrane review, summarizing the findings of 4 placebo-controlled, randomized trials, concluded that when given at a dose of 50 mg twice daily, riluzole is safe and improves median survival by 2-3 months in patients with ALS [29]. In regard to AEs, riluzole was well tolerated, with the exception that treated patients were 2.6 times more likely to experience a threefold increase in serum alanine transaminase (ALT) as compared to patients treated with placebo [29]. This effect was found to be uniformly reversible with cessation of riluzole therapy and was only reported after several months of medication administration. Notably, riluzole is without potent neurotoxic and cardiotoxic adverse effects [25], although potential hepatotoxicity has been noted [30]. In light of these safety and efficacy data, riluzole is the only medication approved by the Food and Drug Administration (FDA) for the chronic treatment of patients with ALS [14].

5.2.4 Riluzole in Other Diseases

In a recent study of prolonged administration of riluzole in Huntington's disease, no benefit was found in slowing disease progression, but riluzole was well-tolerated. Adverse effects were similar in 357 subjects treated with riluzole as compared to 180 placebo subjects. Thirteen subjects had elevated liver enzymes, which led to five subjects discontinuing treatment [31].

Multiple studies are under way to evaluate the efficacy of riluzole in conditions including depression, muscular atrophy, obsessive-compulsive disorder and schizophrenia.

Among the ongoing studies is the AOSpine North America study of efficacy and safety of riluzole in cervical spondylotic myelopathy.

5.2.5 Riluzole in Animal Models of SCI

There is convincing evidence from the preclinical realm that riluzole attenuates certain aspects of the secondary injury cascade leading to diminished neurological tissue destruction in animal SCI models [32-34]. Several studies from a number of independent laboratories, in various species of animals, have shown that riluzole is neuroprotective and promotes functional neurological recovery in models of brain and spinal cord ischemic and traumatic injury [35-38]. Riluzole specifically exerts its neuroprotective effect by helping to maintain neuronal cellular ionic balance and by reducing the release of excitotoxic glutamate in the post SCI setting [39]. Several preclinical rat studies have associated administration of riluzole with increased neural tissue preservation at the site of injury, in addition to improved behavioral outcomes, in comparison to placebo and other sodium-channel blocking medications [33, 34].

Given the lack of synaptic connections within white matter, the axon-sparing property attributed to riluzole can likely be assigned to its ability to decrease the levels of [Na^+] and intracellular calcium [Ca^{2+}] [40].

5.2.6 Pharmacokinetics of Riluzole in SCI Patients

The pharmacokinetics of riluzole in the patients enrolled in the Phase I SCI study have been published previously [41].

In the Phase I study, 50 mg of riluzole was administered every 12 hours either orally or via nasogastric tube, starting within 12 hours of injury for 28 doses. Plasma samples for pharmacokinetic study were collected one to two hours pre-dose and two hours post-dose for trough and peak concentrations, respectively, on days 3 and 14 after the initial dose.

The riluzole dose and dosing schedule was selected using human data and scaling from animal data. From the human data the most conservative approach was used, based on the FDA-approved dose for ALS patients. In confirmatory dose-ranging studies of riluzole in ALS, using doses of 50, 100 and 200 mg/day, a daily dose of 50 mg BID of riluzole was confirmed to have the best benefit-to-risk ratio [24].

Riluzole pharmacokinetics was evaluated in 33 patients on day 3 and in 32 patients on day 14, both C_{peak} and C_{trough} samples were collected and quantifiable. The plasma concentration and the systemic exposure to riluzole (AUC_{0-12}) varied significantly among patients. Maximum concentration (C_{max}) ranged from 24 to 409 ng/ml (mean 129 ± 14 ng/ml, SE) on day 3, and 9 to 317 ng/ml (mean 77 ± 14 SE) on day 14 [41].

The pharmacokinetics of riluzole (C_{max}, C_{min}, AUC_{0-12}, CL/F and V/F) changed during the acute and sub-acute phases of SCI during the 14 days of administration, a phenomenon consistently observed in all patients at all clinical sites. Mean C_{max}, C_{min} and AUC_{0-12} (129 ng/ml, 46 ng/ml and 982 ng *hr/ml, respectively) were significantly higher on day 3 than on day 14 (77 ng/ml, 19 ng/ml and 521 ng *hr/ml, respectively), resulting from lower CL (50 L/hr vs 106 L/hr) and a smaller V (557 L vs 1298 L) on day 3 [41].

In comparing the pharmacokinetic parameters of riluzole in SCI patients with those in healthy volunteers, the C_{max} on Day 3 and Day 14, as well as AUC_{0-12} on Day 14 were lower than those in healthy subjects. However, it may be difficult to determine the cause of this difference due to the different doses given: 9 doses [21] and 20 doses [20] in healthy volunteers versus a maximum of 6 doses on Day 3 and 28 doses on Day 14 in the Phase I trial.

Riluzole pharmacokinetics in SCI were compared to its pharmacokinetics observed in ALS [42] and pediatric spinal muscular atrophy (SMA) [22]. The C_{max} and $AUC_{0-\infty}$ in SCI patients on the same dose basis did not achieve the comparable levels as in ALS or SMA patients, but were lower (128.8 ng/L and 827.8ng*hr/ml on Day 3 and 76.5 ng/ml and 337.8 ng*hr/ml on Day 14) compared to those in patients with ALS (231 ng/ml and 3409 ng*hr/ml) and SMA (371 ng/ml and 2257 ng*hr/ml). The decreased bioavailability (F) in SCI may be due to reduced GI absorption. The apparent clearance (CL/F) and volume of distribution (V/F) in SCI population, 60.4-148 L/hr and 663-2080 L, were substantially higher than those in ALS subjects (25.9 L/hr and 361 L) and SMA patients (22.2 L/hr and 299 L).

5.2.6.1 Safety: Adverse Events and Medical Complications

SCI patients have a high incidence of physiological disturbances and medical complications occurring acutely following injury, as documented in a recent publication of data from the North American Clinical Trial Network (NACTN) SCI registry [43]. Using the definitions of moderate and severe complications described in that paper, the incidence of complications occurring within 30 days of injury was carefully monitored within the Phase I riluzole study. The frequency of these complications in this study were: infection, including pneumonia (39%); pulmonary, including respiratory failure, lobar collapse, atelectasis and pneumothorax (33%); hematological, including anemia, thrombocytopenia and coagulopathy (22%); cardiac, including arrhythmia and shock (14%); psychiatric, including cognitive decline and depression (14%); gastrointestinal/genitourinary, including severe ileus and hematuria (11%); and skin, including pressure sores (8%). The frequency of these complications was similar to that occurring in patients in the NACTN SCI registry [43]. There were no serious adverse events (SAEs) attributable to riluzole. There was no mortality.

5.2.6.2 Safety: Elevation of Liver Enzymes and Bilirubin

Elevation of liver enzymes has been reported in patients with ALS undergoing treatment with riluzole [24]. Elevation of liver enzymes is also known to occur acutely in patients with SCI, and in animal models of SCI, possibly due to impairment of blood flow to the liver [44, 45]. Elevation of alanine aminotransferase (ALT) and aspartate aminotransferase (AST) are considered to be sensitive indicators of drug-induced damage to liver cells. Elevation of gamma-glutamyl transpeptidase (GGT) is a less specific indictor of drug-induced damage to liver cells. Elevation of alkaline phosphatase (ALP) is considered to be primarily an indicator of obstruction of the bile duct.

In the Phase I riluzole in SCI study [41], liver enzymes and bilirubin were monitored on admission and during the administration of riluzole. Elevated levels of liver enzymes and/or bilirubin were found on admission in 9 - 37% of patients. Thirteen percent of patients had mild (> upper limit of normal (ULN) - 2.5x ULN) or moderate (> 2.5 – 5x ULN) elevations of ALT, 37% had mild or moderate elevations of AST, 11 % had mild elevations of GGT and 9% had mild elevations of bilirubin. Some patients had elevation of a single enzyme, while others had 2 or 3 elevated enzymes.

Liver enzyme levels were monitored on days 3, 7, 10 and 14 of administration of riluzole. The incidence of elevation of enzyme levels increased during the administration of riluzole, with increasing frequency in the second week of administration. Two-thirds of patients had mild or moderate elevations of ALT and AST on at least one day of testing. One patient had a severe (>5 – 20x ULN) elevation of ALT (6 x ULN) and another had a severe elevation of AST (5.5 x ULN). These elevations returned to normal at 3 and 6 months. Fifty percent of patients had mild or moderate elevations of GGT and one patient had a severe elevation of GGT (7 x ULN). Seventeen percent had mild or moderate elevations of ALP. Fourteen percent had mild or moderate elevation of bilirubin. No patient had an elevated bilirubin on day 14, the last day of administration of riluzole. The appearance of an increased level of a liver enzyme was not necessarily followed by a progressive increase in the level of that enzyme. In many cases, the elevated concentration had returned to a normal level at the next date of testing. The elevation of one enzyme was not necessarily linked to the elevation of another enzyme.

No relationship was found between the maximal plasma concentration (C_{max}) of riluzole and enzyme levels.

5.2.7 Regulatory Status

Riluzole is approved by the FDA and Health Canada for treatment of ALS. It is not approved by the FDA or Health Canada for treating patients with acute SCI.

The Investigational New Drug (IND) regulations [21 CFR 312.2(b)] state that clinical investigation of a drug product that is lawfully marketed in the United States is exempt from the requirements for an IND if all of the following apply:

1. The investigation is not intended to be reported to FDA as a well-controlled study in support of a new indication for use, nor intended to be used to support any other significant change in the labeling for the drug;
2. The investigation is not intended to support a significant change in the advertising for a prescription drug product;
3. The investigation does not involve a change in route of administration, dosage level, patient population or other factor that significantly increases the risks (or decreases the acceptability of risks) associated with use of the drug product;
4. The investigation is conducted in compliance with the requirements for institutional review (21 CFR Part 56) and informed consent (21 CFR Part 50);
5. The investigation is conducted in compliance with the requirements of 21 CFR 312.7, i.e., the drug may not be represented as safe or effective for the purposes for which it is under investigation, nor may it be commercially distributed or sold.

In addition, 21 CFR 312.2(b) (5) states a clinical investigation involving the use of a placebo is exempt from the IND requirements if the investigation does not otherwise require submission of an IND.

The FDA has been contacted requesting clarification as to whether the trial meets the above requirements for IND exemption. The FDA has determined that the trial meets all of the requirements for exemption and therefore an IND is not required to conduct the investigation in accordance with 21 CFR 312.2(b)(4) (IND 115533; from June 13, 2012).

Health Canada regulates investigator-driven research in the same way as industry-sponsored research. The "No Objection Letter" (equivalent to US FDA IND approval) has been granted by Health Canada to conduct this study.

In other countries, country-specific respective regulatory procedures will be followed and appropriate approvals obtained.

5.2.8 Investigational Drug

5.2.8.1 Preparation of Investigational Drug

Active. The riluzole tablets will be pulverized into powder. Based on the packing statistics of the pulverized powder and desired strength of the capsules needed, microcrystalline cellulose will be added to completely fill the capsules. Capsules will be gelatin-based and BSE- and prion-free certified. This method of preparation is preferred to over-encapsulation of the riluzole tablets in this particular study to allow the contents of the capsule to be administered to participants that are on a nasogastric feeding tube.

Placebo. Same as the active capsules but will only contain microcrystalline cellulose.

5.2.8.2 Packaging

Capsules will be packaged into amber prescription vials that will be labeled with:

- A unique study ID#
- Randomization code #
- Lot#
- Expiration date

Labeling requirements vary in different countries. Country-specific requirements will be followed in labeling.

6 Study Design

6.1 Study Scope

This is an international, multi-center, prospective, double-blinded, randomized, placebo-controlled Phase II/III clinical trial to evaluate if riluzole at a dose of 100 mg BID for the first 24 hours followed by 50 mg BID for 13 days is superior to placebo in subjects with acute traumatic SCI.

The study will involve up to 35 investigational sites and enroll 351 subjects (includes 10% allowance for attrition). A randomization ratio of one riluzole subject to one placebo-controlled subject (1:1) will be utilized. The sample size may change during the interim analysis due to the adaptive statistical design.

6.2 Study Objectives

The aim of this study is to evaluate efficacy and safety of riluzole in the treatment of patients with acute SCI. The primary objective is to evaluate the superiority of riluzole, at a dose of 100 mg BID for the first 24 hours followed by 50 mg BID for the following 13 days after injury, as compared to placebo, in change between 180 days and baseline in motor outcomes as measured by International Standards for Neurological Classification of Spinal Cord Injury Examination (ISNCSCI) Motor Score, in patients with acute traumatic SCI, presenting to the hospital less than 12 hours after injury. Secondary objectives are to evaluate the effects of riluzole on overall neurologic recovery, sensory recovery, functional outcomes, quality of life outcomes, health utilities, mortality, and adverse events. The working hypothesis is that the riluzole treated subjects will experience superior motor, sensory, functional, and quality of life outcomes as compared to those receiving placebo, with an acceptable safety profile.

6.3 Endpoints

6.3.1 Primary Efficacy Endpoint

- Absolute change in International Standards for Neurological Classification of Spinal Cord Injury Examination (ISNCSCI) Total Motor Score (ISNCSCIMS) between 180 days and baseline

6.3.2 Secondary Efficacy Endpoints

- Change in ISNCSCI grade between baseline and 180 days
- Spinal Cord Independence Measure (SCIM) at 180 days

6.3.3 Other Endpoints

- Change in ISNCSCI Sensory Scores (Light Touch and Pin Prick) between 180 days and baseline
- Change in ISNCSCI Upper Extremity Motor Score between 180 days and baseline
- Change in ISNCSCI Lower Extremity Motor Score between 180 days and baseline

- Change in Short Form 36 Version 2 (SF-36v2™) PCS, MCS and 8 dimensions between 180 days and pre-injury (recall)
- Change in EQ-5D health utility between 180 days and pre-injury (recall)
- Graded Redefined Assessment of Strength Sensibility and Prehension (GRASSP) at 14 days or Discharge (whichever occurs first) and 180 days
- Change in Numeric Pain Rating Scale (pain NRS) at 14 days, 84 days and 180 days

6.3.4 Safety Evaluation

Safety evaluation will be a continued effort throughout the study progress. The conduct of the evaluation will be based on 21CFR312.32 (Revised as of April 1, 2013) and the Guidance for Industry and Investigators Safety Reporting Requirements for INDs and BA/BE Studies.[46] Incidence of AEs will be summarized by body system using preferred terminology and compared between the riluzole and placebo arms. All AEs will be listed and all abnormal lab values reviewed. Any treatment-emergent lab AEs will be followed until resolved or declared unresolved. All serious adverse events will be evaluated at the time of the occurrence by the Safety Officer. Trends in AEs and SAEs will be periodically evaluated by the Data Safety and Monitoring Board (DSMB).

6.4 Selection of Investigators and Investigational Sites

Investigators will be selected among board-certified physicians with both clinical experience in the management of spinal cord injury and clinical trial investigator experience. Selected investigational sites will provide evidence of qualification and resources for conducting clinical research to the Sponsor/CRO. Sponsor/CRO may perform site pre-qualification visit.

6.5 Inclusion Criteria

- Age between 18 and 75 years inclusive
- Able to cooperate in the completion of a standardized neurological examination by ISNCSCI standards (includes patients who are on a ventilator)
- Willing and able to comply with the study Protocol
- Informed Consent Document (ICD) signed by patient, legal representative or witness
- Able to receive the Investigational Drug within 12 hours of injury
- ISNCSCI Impairment Scale Grade “A,” “B” or “C” based upon first ISNCSCI evaluation after arrival to the hospital
- Neurological Level of Injury between C4-C8 based upon first ISNCSCI evaluation after arrival to the hospital
- Women of childbearing potential must have a negative serum β-hCG pregnancy test or a negative urine pregnancy test

6.6 Exclusion Criteria

- Injury arising from penetrating mechanism
- Significant concomitant head injury defined by a Glasgow Coma Scale score < 14 with a clinically significant abnormality on a head CT (head CT required only for patients suspected to have a brain injury at the discretion of the investigator)

- Pre-existent neurologic or mental disorder which would preclude accurate evaluation and follow-up (i.e. Alzheimer's disease, Parkinson's disease, unstable psychiatric disorder with hallucinations and/or delusions or schizophrenia)
- Prior history of spinal cord injury
- Recent history (less than 1 year) of chemical substance dependency or significant psychosocial disturbance that may impact the outcome or study participation, in the opinion of the investigator
- Is a prisoner
- Participation in a clinical trial of another Investigational Drug or device within the past 30 days
- Hypersensitivity to riluzole or any of its components
- Neutropenia measured as ANC measured in cells per microliter of blood of < 1500 at screening visit
- Creatinine level of > 1.2 milligrams (mg) per deciliter (dL) in males or > 1.1 mg per dL in females at screening visit
- Liver enzymes (ALT/SGPT or AST/SGOT) 3 times the ULN at screening visit
- Active liver disease or clinical jaundice
- Subject is currently using, and will continue to use for the next 14 days any of the following medications which are classified as CYP1A2 inhibitors or inducers:*

  Inhibitors:
  - Ciprofloxacin
  - Enoxacin
  - Fluvoxamine
  - Methoxsalen
  - Mexiletine
  - Oral contraceptives
  - Phenylpropanolamine
  - Thiabendazole
  - Zileuton

  Inducers:
  - Montelukast
  - Phenytoin

*Note: no washout period required; if these medications are discontinued, subjects are eligible to be enrolled in the trial

- Acquired immune deficiency syndrome (AIDS) or AIDS-related complex
- Active malignancy or history of invasive malignancy within the last five years, with the exception of superficial basal cell carcinoma or squamous cell carcinoma of the skin that has been definitely treated. Patients with carcinoma in situ of the uterine cervix treated definitely more than 1 year prior to enrollment may enter the study
- Lactating at screening visit

6.7 Study Procedures

6.7.1 Screening and Enrollment

Screening and enrollment is schematically shown in Figure 1.

Subjects with acute SCI who present to a participating center within 12 hours of injury will be screened for inclusion. Prior to any screening procedures, subjects will sign the ICD. If the subject meets all of the eligibility criteria, he/she will be randomized and enrolled into the study. The randomization will occur as soon as possible after the eligibility has been determined and the first dose of Investigational Drug will be administered not later than 12 hours from the time of injury. Given the nature of this study and very short period available for the screening, in some rare instances a patient may be enrolled and receive the Investigational Drug prior to receipt of all screening laboratory results. In such rare cases, a clinically significant abnormal value for any of the tests may result in the discontinuation of the patient.

The subject will be considered enrolled into the study when the randomization occurs, and the first dose of study medication is administered.

Figure 1 Screening and enrollment design

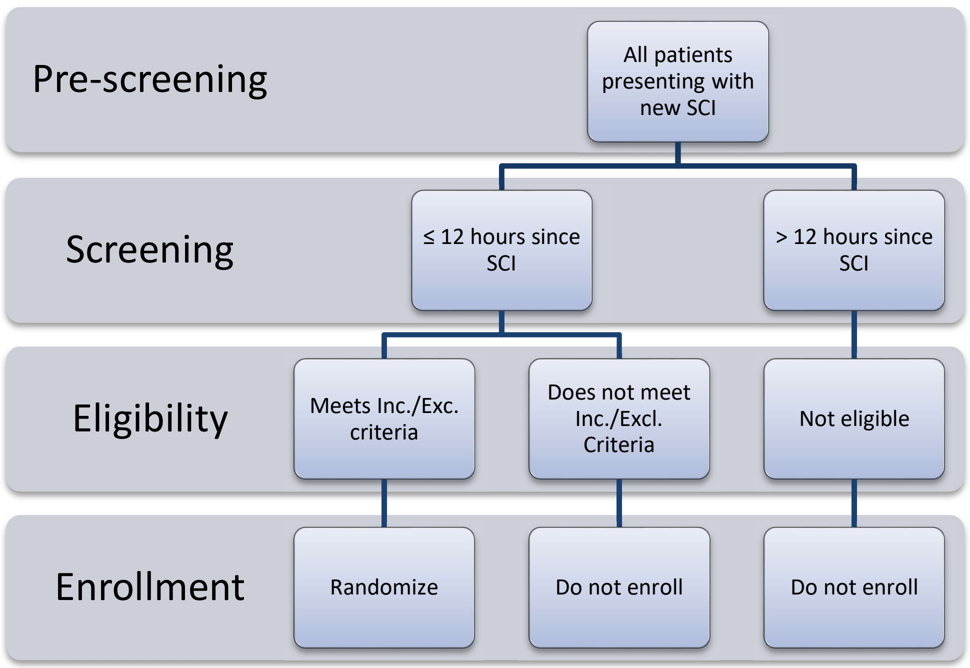

6.7.2 Investigational Drug Dose and Administration

6.7.2.1 Dose

Subjects will receive a total of 28 doses of Investigational Drug, administered twice a day, for a total of 14 days. The riluzole group will receive riluzole in a dose of 100 mg at 0 hours and 12 hours (doses 1 and 2) followed by 50 mg doses every 12 hours for the next 13 days (doses 3 - 28). The control group will receive placebo capsules in a dose of two capsules at 0 hours and 12 hours (doses 1 and 2) followed by one capsule every 12 hours for the next 13 days (doses 3 to 28).

6.7.2.2 Administration of Study Drug

The first two doses of study drug must each be given according to the Protocol schedule (i.e. at 0 hours and 12 hours). The third dose can be administered to align with the standard hospital medication administration schedule. If the next standard hospital medication administration time falls 4 or more hours after the second dose, the third dose should be administered at that time. If the next standard hospital medication administration time falls less than 4 hours after the second dose, that time shall be skipped and the third dose should be administered at the second standard hospital medication administration time. Doses 4 through 28 should be administered every 12 hours.

For example, a hospital's standard medication administration times are 09:00 (9:00 AM) and 21:00 (9:00 PM).

- Patient A's first dose is given at 03:30 (3:30 AM) and the second dose is given at 15:30 (3:30 PM). More than 4 hours elapse before the next standard medication administration time, so the third dose is given at 21:00 (9:00 PM), the fourth dose at 09:00 (9:00 AM) and afterwards every 12 hours.
- Alternatively, Patient B's first dose is given at 06:00 (6:00 AM) and the second dose is given at 18:00 (6:00 PM). The standard medication administration time (21:00) is only 3 hours after the second dose, so the third dose is given at 09:00 (9:00 AM) the next day, the fourth dose at 21:00 (9:00 PM) and afterwards every 12 hours.

For subjects who are unable to receive oral medications, the filler from each capsule will be mixed with 3 mL of water and will be delivered via nasogastric tube.

The investigational drug should be taken at least an hour before, or two hours after, a meal to avoid a food-related decrease in bioavailability. If the subject is on a continuous feeding schedule via nasogastric tube, it is recommended that feeding is stopped to accommodate the above drug administration schedule, as long this is not clinically contraindicated. If there is a clinical contraindication to stop feeding via nasogastric tube, the drug should be administered without interruption of continuous feeding.

6.7.2.3 Monitoring Liver Chemistries and Investigational Drug Discontinuation

Liver chemistries will be monitored in all subjects as riluzole can increase serum aminotransferase, even in patients without a prior history of liver abnormality. Enzymes (ALT, AST, ALP and GGT) and serum bilirubin will be monitored on days 3, 7 and 14. The procedure to follow-up subjects with elevated enzymes and decision to discontinue medication will be made in accordance with the FDA Guidance for Industry "Drug-Induced Liver Injury: Premarketing Clinical Evaluation," 2009.

Follow-up of subject with increase of serum AT to >3xULN

- If enzyme elevation is greater than 3 times normal, repeat testing within 48 - 72 hours including ALT, AST, ALP and bilirubin to confirm the abnormalities and to determine trend.

- Monitor for clinical symptoms of hepatic injury such as nausea, vomiting, fever, right upper quadrant pain or tenderness, rash or eosinophilia (> 5%).
- Other clinical maneuvers and tests should be per standard of care.

Criteria to discontinue Investigational Drug due to possible drug induced liver injury

- ALT or AST >8xULN
- ALT or AST >3xULN and (TBL >2xULN or International Normalized Ratio (INR) >1.5)
- ALT or AST >3xULN with the appearance of fatigue, nausea, vomiting, right upper quadrant pain or tenderness, fever, rash, and/or eosinophilia (>5%)

If the patient reaches the above criteria to discontinue investigational medication the blind will not be broken, except in the case of patient safety concerns. All trial subjects showing possible investigational drug-induced liver injury will be followed until all abnormalities return to normal or to the baseline state, until declared clinically insignificant or until the status is declared as unresolved. However, no subjects will be followed-up beyond the final study follow-up visit.

6.7.3 Steroid Administration

Intravenous methylprednisolone sodium succinate (MPSS) administration will be permitted in the study according to the discretion of the site specific principal investigator. However, when administered, only the dosing regimen prescribed by the second National Spinal Cord Injury Study will be permitted:

- MPSS IV 30mg/kg bolus given over first hour, followed by 5.4 mg/kg/hour for 23 hours

6.7.4 Blinding

Subjects, Investigators, and study research personnel will be blinded to the subject treatment arm allocation. The AOSpine North America Methods Core will maintain link between the randomization information and the Investigational Drug information. This information will be accessible only to selected restricted personnel at the Methods Core, which will not include study monitors or other personnel in direct contact with the study sites. Treatment allocation will be un-blinded for the purpose of interim analysis presented to the DSMB. The results of the interim analysis and the un-blinded data will not be shared with Investigators, subjects or those involved in the execution of the study. Emergency un-blinding for the safety reasons will be permitted. Sites will be supplied with emergency un-blinding materials.

6.7.5 Enrollment

Subject enrollment occurs at the time of Randomization. If a subject is consented, screened and for any reason does not get randomized, the subject will NOT be considered enrolled in the study.

The randomization will occur by opening the lowest sequential number of the sealed randomization envelopes at each specific site. Inside the envelope there will be a unique subject randomization number. The authorized research staff will match the unique subject

randomization number with the number on the Investigational Drug container. The randomization will be recorded in the Randomization Log.

6.7.6 Follow-up Evaluations

Subjects will be evaluated according to the schedule set forth in this clinical Protocol. The schedule includes enrollment; surgery; 72 ± 12 hours post-injury; 7 days ± 1 day after enrollment; 14 days + 2 days; discharge from acute care; 84 days ± 14 days; 180 days ± 30 days; and 365 days ± 45 days. The study will continue until the last enrolled subject reaches the 180-day follow-up. As a consequence, a portion of subjects will not have 365-day follow-up visit.

All follow-up schedules are based on “Day 0” which for this study is the day of screening/enrollment visit, except for the visit 72 hours post-injury.

6.7.7 Study Schematic

The schedule of visits and data to be collected at each visit is shown in Table 1. All visits are preferred to be performed in the clinic. However, if the subject is unable to visit the clinic, a study investigator or an assigned examiner may visit the subject to collect the data. Data collection by phone and/or mail is not permitted.

Table 1 Schedule of study activities

	Screening / Enrollment	Surgery (if applicable)	72 hours ± 12 hours post-injury	7 days ± 1 day post-enrollment	14 days + 2 days	Discharge from Acute Care	84 days ± 14 days	180 days ± 30 days	365 days ±45 days	Unscheduled Visit
Sign ICD	X									
Health Information Release Form (if applicable)	X									
Review Inclusion/Exclusion	X									
Obtain Demographics	X									
Screening Labs	X									
Clinical Labs			X	X	X					
Pregnancy Test (if applicable)	X									
ISNCSCI	X		X		X		X	X	X	
Randomization	X									
Dispense Investigational Drug	X									
Complete Investigational Drug Log	X				X					
Medication Compliance SW[&]					X					
Charlson Comorbidity Score	X									
Injury Severity Score	X									
SF-36v2.0™	X[$]						X	X	X	
EQ-5D	X[$]						X	X	X	
Obtain & complete socio-economic & health behavior SWs	X									
Obtain & complete medical history SWs	X									
Spine Trauma Injury Data SW	X									
Concomitant Medications	X	X	X	X	X	X	X	X	X	X
Vital Signs	X	X	X		X	X				
Record Operative Data		X								
MRI**			X**							
SCIM					X		X	X	X	
GRASSP					X***	X***		X		
Pain NRS					X		X	X	X	
Report AEs & SAEs (including intra-operative)		X	X	X	X	X	X	X	X	X
Discharge Information						X				
Physical & Occupational Therapy						X	X	X	X	
Verify Data & Enter into eCRF within 48 hours*	X	X	X	X	X	X	X	X	X	X

*Data should be entered into the eCRF within 48 hours, but no later than 14 calendar days from collection; **MRI between 48-72 hours at the discretion of the Investigator; [&] Medication compliance should be completed daily; ***GRASSP will be performed at 14 days or Discharge (whichever occurs first); [$] Recall of status prior to the injuryStudy Timeline

6.7.7.1 Pre-screening

Patients receiving care at the investigative sites will be pre-screened as potential subjects for the study. In order to do so, only the existing information obtained per standard routine medical procedures will be used. Pre-screening information (without identifiable information) will be obtained and transferred to Sponsor/ CRO. No study-specific screening procedures, activities or questionnaires will be performed during the pre-screening.

6.7.7.2 Screening/Enrollment

Patients considered potential candidates for the study based on pre-screening will sign an Institutional Review Board (IRB)/Review Ethical Board (REB)/Ethical Review Committee (ERC) approved ICD <u>prior to participating in any study activities.</u>

The following procedures are performed during the visit:

- Obtain signed ICD, place it in the patient file, provide copy to subject
- Obtain signature on the health information release form (when applicable)
- Assign a Subject Investigational Code (SIC)
- Verify that the patient meets all inclusion/exclusion criteria and perform the following:
    - Demographics
    - Screening clinical laboratory blood tests (liver enzymes (ALT/SGPT and AST/SGOT), creatinine, ANC)
    - Pregnancy test (if applicable)
- Perform and record ISNCSCI examination
- Review and verify all data collected to confirm the subject meets all study inclusion/exclusion criteria
- Check Screening Source Worksheets (SW) for completeness
- **Enroll subject:** Perform randomization procedure and enter randomization code into the Randomization Log
- Ensure Screening Form is updated with the enrollment information
- Administer *Dose # 1* of Investigational Drug and record on the Investigational Drug Dispensing SW
- Log the subject's medication accountability information into the Investigational Drug Accountability Log
- Log Investigational Drug administration into Medication Compliance SW
- Make a note in the subject's chart not to take medications on the exclusion list for 14 days
- Complete Charlson Comorbidity Score
- Obtain Injury Severity Score
- Subject completes questionnaires (recall of the status prior to the injury)
    - SF-36v2™, EQ-5D
- Obtain socio-economic, health behavior, medical history and information about concomitant medications
- Complete Spine Trauma Injury Data SW
- Record date and time of hospital admission
- Check and record concomitant medications
- Obtain and record vital signs

- Ensure the subject receives Investigational Drug during the hospital stay
- Record date and time of injury and other injury details
- Check Enrollment SWs for completeness
- Verify data and enter into electronic Case Report Form (eCRF) preferably within 48 hours but no later than14 calendar days from collection

6.7.7.3 Surgery

- Record operative data
- Check and record concomitant medications
- Obtain and record intra-operative complications
- Record immediate postoperative AE/SAE
- Obtain and record vital signs
- Check SWs for completeness
- Verify data and enter into eCRF preferably within 48 hours but no later than 14 calendar days from collection

6.7.7.4 72 hours ± 12 hours

The following activities should be performed during the visit:

- Perform and record ISNCSCI
- Obtain and record concomitant medications
- Obtain and record AE/SAE
- Obtain and record vital signs
- Order clinical lab tests
- Obtain MRI between 48 -72 hours post-injury (at the discretion of the investigator or if standard of care)
- Check SWs for completeness
- Verify data and enter into eCRF preferably within 48 hours but no later than 14 calendar days from collection

6.7.7.5 7 days ± 1 day

The following activities should be performed during the visit:

- Order clinical lab tests
- Obtain and record concomitant medications
- Obtain and record AE/SAE
- Check SWs for completeness
- Verify data and enter into eCRF preferably within 48 hours but no later than 14 calendar days from collection

6.7.7.6 14 days +2 days

The following activities should be performed during the visit:

- Obtain and record concomitant medications
- Obtain and record AE/SAE
- Obtain and record vital signs
- Investigator performs and records:
  - ISNCSCI
  - SCIM
  - GRASSP evaluation (if discharge from acute care has not yet happened)
- Order clinical lab tests
- Subject completes questionnaires for:
  - Pain NRS
- Complete Medication Compliance SW
- Schedule next study visit
- Check SWs for completeness
- Verify data and enter into eCRF preferably within 48 hours but no later than 14 calendar days from collection

6.7.7.7 Discharge from acute care

The following activities should be performed during the visit:

- Obtain and record concomitant medications
- Obtain and record AE/SAE
- Obtain and record vital signs
- Perform GRASSP if discharged from acute care before 14 days follow-up
- Obtain and record physical and occupational therapy information
- Complete discharge information
- Schedule next study visit
- Check SWs for completeness
- Verify data and enter into eCRF preferably within 48 hours but no later than 14 calendar days from collection

6.7.7.8 84 days ± 14 days

The following activities should be performed during the visit:

- Obtain and record concomitant medications
- Obtain and record AE/SAE
- Obtain and record physical and occupational therapy information
- Investigator performs and records:
  - ISNCSCI
  - SCIM
- Subject completes questionnaires for:
  - EQ-5D
  - SF-36v2™
  - Pain NRS
- Schedule next study visit
- Check SWs for completeness

- Verify data and enter into eCRF preferably within 48 hours but no later than 14 calendar days from collection

6.7.7.9 180 days ± 30 days

This is the main endpoint study visit. The following activities should be performed during the visit:

- Obtain and record concomitant medications
- Obtain and record AE/SAE
- Obtain and record physical and occupational therapy information
- Investigator performs and records:
  - ISNCSCI
  - SCIM
  - GRASSP evaluation
- Subject completes questionnaires for:
  - EQ-5D
  - SF-36v2™
  - Pain NRS
- Schedule next study visit (if applicable)
- Check SWs for completeness
- Verify data and enter into eCRF preferably within 48 hours but no later than 14 calendar days from collection

6.7.7.10 365 days ± 45 days

The following activities should be performed during the visit:

- Obtain and record concomitant medications
- Obtain and record AE/SAE
- Obtain and record physical and occupational therapy information
- Investigator performs and records:
  - ISNCSCI
  - SCIM
- Subject completes questionnaires for:
  - EQ-5D
  - SF-36v2™
  - Pain NRS
- Check SWs for completeness
- Verify data and enter into eCRF preferably within 48 hours but no later than 14 calendar days from collection

6.7.7.11 Unscheduled Visit

- Obtain and record concomitant medications
- Obtain and record AE/SAE
- Check SWs for completeness

- Verify data and enter into eCRF preferably within 48 hours but no later than 14 calendar days from collection

6.7.7.12 Dispensing the Investigational Drug

Each study site will be supplied with the Investigational Drug. All Investigational Drug containers will have an identical appearance. Each container will be labeled with a unique number that will match a number in a randomization envelope.

Authorized site research staff will select the Investigational Drug package to dispense to the subject by matching the container label to the unique randomization number identified in the randomization envelope.

- Authorized Hospital Staff/Nurse will be instructed as follows:
    - Subject to be given one capsule every 12 hours via oral or NG administration (see additional explanations at Section 6.7.2)
    - The investigational drug should be taken at least an hour before, or two hours after a meal to avoid a food-related decrease in bioavailability. If a dose is missed, the next dose should be administered as originally planned

6.7.7.13 Breaking the Randomization Code

The AOSNA Methods Core is responsible for randomization assignment. The sites will be provided with the un-blinding information in case of emergency. In the event of an emergency, the principal investigator at the site will make the decision regarding the un-blinding. The investigator should not open the investigational product assignment blind unless knowledge of the subject's treatment is required for the subject's clinical care and safety. Documentation of breaking the blind should be recorded with the reason, date and time the blind was broken and the names of the personnel involved. The reason for un-blinding will be submitted to Sponsor/CRO within 24 hours and to IRB/REB/Ethical Committee per their regulations.

6.7.7.14 Storage

Investigational product/placebo must be stored in a secure, locked cabinet with limited access. Neither the Investigator nor any member of the study staff will distribute any of the Investigational Drug to any person not participating in this study. The drug should be stored at a temperature between 15°-30°C (59°-86°F) and protected from bright light.

The Investigational Drug will be dispensed at the discretion and by the direction of the Investigator in accordance with the conditions specified in this Protocol. It is the Investigator's responsibility to ensure that accurate records of Investigational drug issuance and return are maintained.

It is expected that the site staff will maintain temperature logs in the investigational product storage area, recording the temperature at least once each working day.

6.7.7.15 Investigational Drug Accountability

The investigational site will maintain an inventory of the Investigational Drug. This will include:

- Name of person designated as responsible for the inventory of the investigational product
- Amount received including date, and lot number
- Amount currently in inventory
- Amounts dispensed to each subject, identified by subject initials and a unique subject study number
- Amount destroyed, if applicable – this should not occur without prior notification to Sponsor/CRO
- Non-study disposition (e.g. wasted, broken)
- Amount returned to Sponsor or designee, if applicable

Sponsor, or its designee, will provide forms to facilitate investigational product inventory control. All investigational product accountability forms and treatment logs must be retained in the site's regulatory binder. These records must be available for inspection by the Sponsor, its designees or by regulatory agencies at any time.

Investigational Drug shipment to sites will be accompanied by a Drug Shipment Form which must be signed and dated upon receipt and copy sent back to the Sponsor/CRO. This form must be filed in the site regulatory binder.

6.7.7.16 Assessment of Subject Compliance

Compliance will be recorded by the Study Research Coordinator or Investigator during enrollment at 14-day post-enrollment and will be documented on the appropriate SW (Medication Compliance SW). Subjects must have 80% or greater compliance. The subject is considered compliant if he/she has taken at least 24 capsules.

Failure to comply will be considered a Protocol Violation and will be recorded on the Protocol Violation Form. Non-compliant subjects will remain in the study and will undergo the same study procedures as compliant subjects.

6.7.8 Medical and Surgical Care

Medical care for the acute SCI, including blood pressure management, will follow established standards of care [15]. Decompressive surgery for SCI in the acute setting post-injury will be performed as per standard of care at the treating institution. Wherever possible, however, surgical decompression of the spinal cord will be undertaken within 24 hours of injury. The surgical approach, medical devices and bone grafts/bone graft substitutes used will be at the surgeon's discretion.

6.7.9 Post-surgical Rehabilitation and Occupational Therapy

Post-treatment rehabilitation and occupational therapy procedures will be per standard of care at the investigational site. The number of hours of rehabilitation and occupational therapy will be recorded.

6.7.10 Clinical Laboratory Procedures

Blood draws for the clinical laboratory examinations will be taken at screening and days 3, 7 and 14 after the treatment initiation (Table 2).

Table 2 Clinical lab blood tests

Examination	Screening	72 hours ± 12 hours	7 ± 1 days post treatment	14 + 2 days post treatment
**AST (SGOT)**	X	X	X	X
**ALT (SGPT)**	X	X	X	X
**ALP**	X	X	X	X
**GGT**	X	X	X	X
**Bilirubin**	X	X	X	X
**INR**	X	X	X	X
**Creatinine (mg/dL)**	X	X	X	X
**Red Blood Cell (RBC)**	X	X	X	X
**White Blood Cell (WBC)**	X	X	X	X
**Hemoglobin (Hbg)**	X	X	X	X
**Hematocrit (Hct)**	X	X	X	X
**Neutrophils**	X	X	X	X
**Immature Polys**	X	X	X	X
**Lymphocytes**	X	X	X	X
**Monocytes**	X	X	X	X
**Eosinophils**	X	X	X	X
**Basophils**	X	X	X	X
**Platelet Count**	X	X	X	X
**Mean Cell Hemoglobin Concentration (MCHC)**	X	X	X	X
**Reticulocyte**	X	X	X	X
**ANC**	X	X	X	X

6.8 Subjects Completion and Disposition

6.8.1 Screen Failure

Screen failure is defined as a subject who has signed the ICD but does not meet the eligibility criteria, was not enrolled by the Investigator for any reason or withdraws consent for any reason

prior to enrollment. A subject may be considered a screen failure any time prior to enrollment. Screening SWs will be completed for all screen failures, and the reason for failure will be indicated.

6.8.2 Enrolled Subject

A subject is considered to be enrolled into the trial when Randomization is completed.

6.8.3 Withdrawn (Discontinued) Subject

Reasonable efforts to keep each subject in the study will be made and must be documented by the Investigator. A subject will be withdrawn from the study for any of the following reasons:

- In rare cases subject may be enrolled prior to receiving all screening laboratory tests. If the results show clinically significant abnormalities, the subject may be discontinued.
- Subject voluntarily withdraws consent after enrollment and terminates participation.
- The Investigator withdraws the subject. If this decision is made for safety reasons or non-compliance with the study Protocol or procedures the Sponsor/CRO will be notified immediately.
- The Investigator or the Sponsor stops the study or stops the patient's participation for medical, safety, regulatory or other reasons consistent with applicable laws, regulations and Good Clinical Practice (GCP).

For each case, detailed information will be obtained explaining circumstances leading to the withdrawal. This will be recorded on the Subject Withdrawal Form. Investigational Drug assigned to the withdrawn subject shall not be assigned to another subject. The remaining study medication for the withdrawn subject will be obtained from the subject and kept at the site to be processed at the end of the study according to the disposal or return instructions.

For safety reasons, a subject who withdraws from the study for any reason before completion of the dose regimen and the last scheduled lab test will be assessed for safety evaluation purposes. This shall occur within 30 days of the last dose of the Investigational Drug.

6.8.4 Lost to Follow-up

A subject will be considered lost to follow-up if he/she does not appear for the scheduled study visit and study personnel are unable to contact the subject. Study personnel must make a reasonable effort to contact the subject and document the following contact attempts prior to declaring a subject to be lost to follow-up: three phone calls with at least 2 days in between each call followed by a certified letter. The first phone call must occur no less than 3 business days after subject has failed to show up for a visit.

6.8.5 Completed Subject

A subject will be considered as having completed the study if he/she has completed all assessments through the 180-day visit. The 365-day visit will be used until the last subject completes the 180-day follow-up visit.

6.9 Study Site Discontinuation

Study site participation may be discontinued if the Sponsor, the Investigator or the IRB/REB/ERC of the study site determines it necessary for medical, safety, regulatory or other reasons consistent with applicable laws, regulations and GCP.

6.10 Study Discontinuation

The study will be discontinued if the Sponsor decides it necessary for medical, safety, regulatory or other reasons consistent with applicable laws, regulations and GCP. The study may be also discontinued if it reaches a futility conclusion during the interim analysis.

7 Statistical Analysis

Details of the statistical analysis, including the adaptive changes and handling of missing values will be described in the Statistical Analysis Plan (SAP), which will be provided as a separate document. This section provides key elements of the statistical approach.

7.1 Primary Efficacy Endpoint

The primary efficacy endpoint in this study is absolute difference in ISNCSCI Motor Score (ISNCSCIMS) between 180-day follow-up and baseline

$$\Delta ISNCSCIMS_{180\text{-}b} = ISNCSCIMS_{180\ days} - ISNCSCIMS_{baseline} \quad (1)$$

7.2 Secondary Efficacy Endpoints

- Change in ISNCSCI grade between baseline and 180 days
- SCIM at 180 days

7.3 Other Endpoints

- Change in ISNCSCI Sensory Score (Light Touch and Pin Prick) between 180 days and baseline
- Change in ISNCSCI Upper Extremity Motor Score between 180 days and baseline
- Change in ISNCSCI Lower Extremity Motor Score between 180 days and baseline
- Change in SF-36v2™ PCS, MCS and 8 dimensions between 180 days and pre-injury (recall)
- Change in EQ-5D health utility between 180 days and pre-injury (recall)
- GRASSP at 84 days and 180 days
- Change in pain NRS for at 14 days, 84 days, 180 days

7.4 Adverse Events

AEs will be recorded according to descriptions provided in Section 9.

7.5 Study Success

Study success is defined as follows:

Investigational treatment (riluzole) is superior to placebo. Study success is achieved if the one-sided null hypothesis of no superiority of riluzole group in $\Delta ISNCSCIMS_{180\text{-}b}$ is rejected.

7.6 Statistical Testing of Primary Endpoint

The appropriate statistical approach is to test a single one-sided null-hypothesis that the difference between the investigational and the placebo arm is equal to or less than 0. Rejection of the null-hypothesis is consistent with superiority of the investigational treatment to the placebo.

$$H0: \mu_I - \mu_c \leq 0$$

$$H1: \mu_I - \mu_c > 0$$

Where:

μ_I and μ_C are the means of the two independent normal distributions;

μ_I is the mean population value of the $\Delta ISNCSCIMS_{180\text{-}b}$ in the patients treated with riluzole;

μ_c is the mean population value of the $\Delta ISNCSCIMS_{180\text{-}b}$ in the patients treated with placebo.

The hypothesis will be tested by one-sided t-test at the alpha level of 0.025. If the test value exceeds the nominal critical point, the H0 will be rejected. If the test-value crosses the futility boundary, the H0 will be accepted.

7.7 Statistical Testing of Secondary Endpoints

Secondary endpoints will be tested for superiority. The statistical significance will be established by two-sided $\alpha = 0.05$.

7.8 Safety Evaluations

AEs will be classified by body system and preferred term. Relative frequencies of AEs will be compared between the riluzole and placebo arms using chi-square test with alpha = 0.05.

7.9 Sample Size Estimate

The required sample size is 316 evaluable subjects, or 158 subjects per study arm. The rationale for the sample size is as follows.

Sample size has been calculated to provide 90% power in testing the primary superiority hypothesis. The estimate has been calculated by PROC SEQDESIGN for SAS/STAT. Standard deviation for the primary outcome parameter has been made using the data from the STASCIS study (data on file with the Sponsor) (Table 3). This estimate is based on subjects with neurological level of injury from C4-C8 at arrival, the ISNCSCI Impairment grade “A,” “B” or “C” and time from injury to arrival of less than 12 hours.

Table 3 Standard deviation estimate for the primary efficacy endpoint

Endpoint	Standard Deviation
$\Delta ISNCSCIMS_{180\text{-}b}$	24.08

Table 4 Sample size estimate ΔISNCSCIMS$_{180\text{-}b}$

Plan ID	Parameter
Type of the Hypothesis	1-Sided
Type I Error (α)	0.025
Power (1 - β)	0.90
Randomization Ratio (Investigational vs. Control)	1:1
Planned Number of Interim Looks	2
Spacing of Looks	60%, 100%
Hypothesis to be Rejected	H0 or H1 (binding)
Boundary Family	Published Function
Boundary to Reject H0	Est. O'Brien-Fleming
Boundary to Reject H1	Gamma (-1)
Difference of Means Assuming H_1	9
Standard Deviation (σ)	24.08
Sample Size	316 (158 per arm)

Under the above assumptions, the estimated sample size is 316 evaluable subjects (158 in the riluzole arm and 158 in the placebo arm). In order to account for loss of power due to loss of follow-up and possible adjustments for baseline factors we will increase sample size by 10% to **351** *enrolled* subjects.

7.10 Justification for Effect Estimate

There is no published minimally significant difference for ISNCSCIMS. The current effect estimate of 9 is arbitrarily set. This difference translates to 0.37 Cohen's d and is at the lower boundary of the moderate effect.

7.11 Statistical Performance and Simulations

The statistical plan aims at efficient design that allows the demonstration of treatment efficacy while also allowing for early stop due to futility if the treatment does not appear to be successful.

Figure 2 shows rejection boundaries for H0 and H1 on the absolute delta scale for the interim and the final analysis.

Figure 2 Rejection boundaries for ISNCSCI motor score

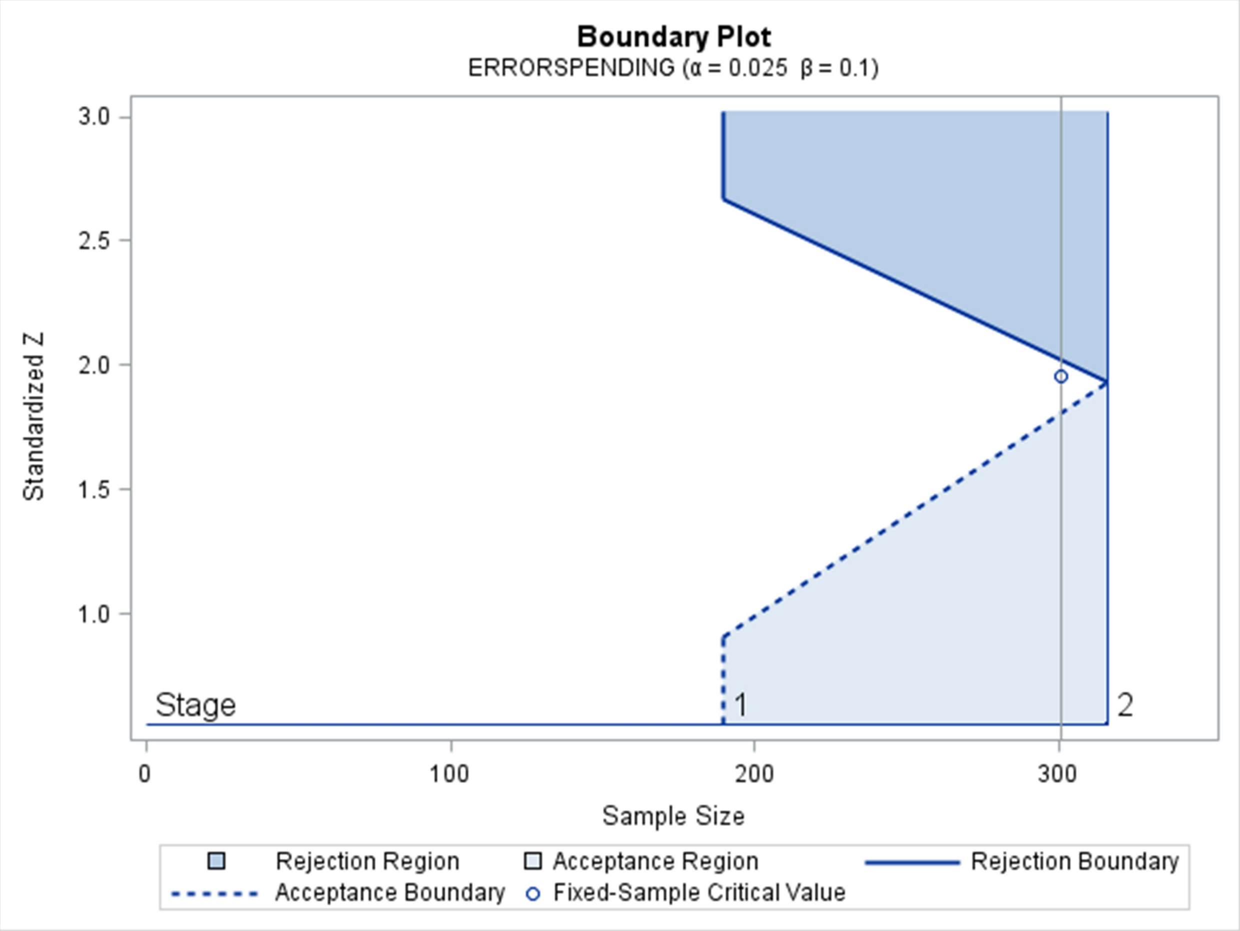

Table 5 shows the results of the statistical simulations of trial performance. Under the futility scenario (i.e. the difference in treatment effect between the placebo and riluzole equals zero), the trial has 81.8% probability to stop at the interim analysis and 2.5% probability to wrongly reject H0 at the final analysis. The 2.5% probability is consistent with chosen alpha level for the study. If the true effect is 50% of the anticipated effect (i.e. 4.5 points difference between the cohorts assuming no changes in the SD), the trial will have 62.9% power, before possible sample size adaptive adjustments. If the effect size is as expected, the trial will have 46.2% power at the interim and 90% power at the final analysis. Finally, if the effect is for 50% larger than anticipated (i.e. 13.5 points difference and the same SD), the trial will have 88.3% power at the interim and 99.8% at the final data look, before possible sample size adjustment.

Table 5 Statistical simulation of trial performance

True Difference	Expected Stopping Stage	Source	Stopping Probabilities	
			Interim Analysis	Final Analysis
**0.0000**	1.179	Reject Null	0.00381	0.02500
**0.0000**	1.179	Accept Null	0.81764	0.97500
**0.0000**	1.179	Total	0.82145	1.00000
**4.5000**	1.565	Reject Null	0.08343	0.37114
**4.5000**	1.565	Accept Null	0.35203	0.62886
**4.5000**	1.565	Total	0.43546	1.00000
**9.0000**	1.490	Reject Null	0.46172	0.90000
**9.0000**	1.490	Accept Null	0.04785	0.10000
**9.0000**	1.490	Total	0.50957	1.00000
**13.5000**	1.115	Reject Null	0.88301	0.99761
**13.5000**	1.115	Accept Null	0.00158	0.00239
**13.5000**	1.115	Total	0.88459	1.00000

7.12 Adaptive Design and Sample Size Re-estimate

The current sample size estimate is based on numerous assumptions. The main assumption is that of the true effect size and the standard deviation for the $\Delta ISNCSCIMS_{180\text{-}b}$. These assumptions will be verified during the study and sample size readjustment will be performed if needed, using the adaptive techniques. Other adaptive adjustments may occur at the same time. The adaptive adjustments will be performed after the first interim analysis of the data, currently planned at 60% data available at 180 days. The details of the adaptive changes will be described in a separate SAP.

7.13 Analysis Populations

For definitions of subjects' status in the study see Section 6.8.

7.13.1 Intention-to-Treat

Consenting subjects who qualify, are randomized to a study arm and receive at least one dose of study medication (i.e. enrolled subjects) will be included in the Intent-To-Treat (ITT) analytic

sample primary analysis population, regardless of the actual treatment received. Screen failures will be recorded on the Screening SW and will not be included in the ITT group. Values for the subjects who do not have the 180 day end point will be imputed to create a complete ITT population.

7.13.2 Modified Intention-to-Treat

The modified Intent-To-Treat (mITT) population is defined as all consenting subjects who receive at least one dose of study-directed treatment and have any follow-up. The mITT population will be used for safety analyses.

7.13.3 Completed Cases Population

The Completed Cases (CC) population is defined as all enrolled subjects and eligible patients who received at least one dose of study-directed treatment (medication) and had 180 days of follow-up defined as any efficacy measurement taken at this visit.

7.13.4 Per-protocol Population

The Per-protocol population (PP) population is a subset of CC population and it includes all enrolled patients entirely consistent with the Protocol and who received at least 80% of study-directed treatment and had 180 days of follow-up. Any visits where Protocol violations occurred will be reviewed for possible exclusion from PP population.

The patients with major Protocol violations will be tabulated by violation type and affected visit(s), where applicable. All Protocol violations will be listed.

7.13.5 Primary Analysis Populations

Primary efficacy analysis will be performed on the ITT population. Primary safety analysis will be performed on the mITT population.

7.14 Randomization

The randomization will be performed at 1:1 ratio between the investigational and control arms. Randomization will be carried out according to the blocks procedure. Block randomization is used to assign eligible subjects to the treatment arms in order to avoid serious imbalance in the number of subjects assigned to each group within a center. The pattern of the blocks will be concealed to avoid selection bias. Two block sizes will be used, with 2 and 4 subjects per block. Block sizes will be chosen at random. This scheme makes breaking the blinding by working out the block pattern extremely difficult and reasonably protects against biased allocation of subjects.

7.15 Evaluation of 365 days follow-up data

365 days follow-up will be available for majority, but not for all subjects. The main purpose of the 365 days follow-up data is to evaluate sustainability of the possible effect. The sustainability analysis will be based on repeated measurement analysis and testing of interaction terms between the treatment arm and follow-up time as well as Bayesian analytic approaches. The details will be described in SAP.

7.16 Pre-planned Subgroup Analyses

A pre-planned sub-group analysis will be conducted to evaluate differences in $\Delta ISNCSCIMS_{180\text{-}b}$ among the patients in baseline ISNCSCI Impairment Groups "A," "B" and "C." The rationale for this pre-planned analysis is that these groups experience different recovery of ISNCSCIMS which may result in between-group differences in $\Delta ISNCSCIMS_{180\text{-}b}$. Motor recovery, as measured by change in ISNCSCIMS between acute admission and 6 months follow-up, is known to vary according to individuals' baseline level of injury severity, as defined by ISNCSCI grade. For instance, for cervical patients, in data obtained from the NACTN prospective registry, the average improvement in ISNCSCIMS from admission to 6 months follow-up was 12.4 (SD=22.3) points for ISNCSCI grade "A" patients, 27.0 (SD=26.3) points for ISNCSCI grade "B" patients and 52.3 (SD=24.1) points for ISNCSCI grade "C" patients. The variability in motor recovery depending on the baseline injury severity has been fully incorporated into the sample size estimate calculations described in Section 7.9. At present, the data from the Phase I study of riluzole in SCI are under analysis. Such data, when available, will be incorporated in the planning of this sub-group analysis. However, this pre-planned sub-group analysis is of interest from an exploratory perspective and will be used for purposes of planning future studies which may serve to further refine riluzole's role in the treatment of SCI. Statistical details of this analysis will be provided in the SAP.

Other pre-planned sub-group analyses may be performed. They will be pre-specified in the SAP before any study data is un-blinded or analyzed for efficacy.

8 Risk Analysis

8.1 Risks

The risks that occur in this study are associated with the general risks of managing spinal trauma and SCI patients.

Anticipated AEs which may occur as a direct result of SCI, decompression (or other) surgery or general anesthesia will be present regardless of the participation in the trial. These risks will be described in ICD.

The events cited in Table 6 reflect all AEs reported in placebo-controlled trials of ALS patients treated with either riluzole 100mg/day or placebo, where the incidence in the riluzole group was greater than placebo by at least 1%.

Table 7 shows the most common TEAEs considered possibly related to study medication occurring in a post-marketing study and for which the incidence was ≥ 1%.

Table 6 Incidence of AEs in placebo-controlled trials

Preferred Term	Riluzole (100 mg/day) (N=395) (Reported as %)	Placebo (N=406) (Reported as %)
Asthenia	17	11
Pain	5	2
Nausea	14	9
Abdominal Pain	5	4
Vomiting	4	2
Paraesthesia oral	1	0
Headache	7	6
Dizziness	3	2
Somnolence	2	1
Tachycardia	3	2
Alanine aminotransferase (ALT/SGPT) abnormal	29	14
Aspartate aminotransferase (AST/SGOT) abnormal	23	19
Gamma-glutamyltransferase (GGT) abnormal	15	11
Blood bilirubin abnormal	12	8

Table 7 The most common TEAEs considered possibly related to study medication occurring in a post-marketing study and for which the incidence was ≥ 1%

SYSTEM ORGAN CLASS	Preferred Term	N=414 (%)
Gastrointestinal Disorder	Nausea	6
	Stomach Discomfort	3
	Diarrhea	2
	Dyspepsia	1
	Constipation	1
	Hypoaesthesia oral	1
General disorders and administration site condition	Fatigue	4
Nervous system disorder	Dizziness	4
	Headache	1
	Dysgeusia	1
Psychiatric Disorders	Insomnia	1
Skin and Subcutaneous Tissue Disorders	Rash	1
	Pruritus	1
Hepatobiliary Disorders[&]	Alanine aminotransferase (ALT/SGPT) abnormal	1

[&]Alert terms (ALT and/or AST values >10 times the upper limit of normal) or abnormal values that led to Investigational Drug termination

Other side effects associated with use of riluzole, with a frequency of < 1%, may occur.

8.2 Actions to Minimize Increased Risks

The surgical procedures used in this study are considered standard of care. The investigational treatment of this study is an off-label use of an approved drug. Study-related procedures involve non-invasive clinical examinations and patient questionnaires.

Risks to the patients are managed by:

- Use of an approved drug with known side effects profile
- Continuous monitoring of safety events by Safety Officer
- Pre-planned stopping rules for safety discontinuation of the study during the interim analysis by an independent DSMB
- Interim efficacy analysis to determine an early stop in the case of futility
- Monitoring of liver chemistry
- Procedure for un-blinding in the case of emergency
- GCP compliant procedures to manage patient confidentiality and study operations

9 Adverse Event Reporting

AE information will be collected throughout the study and all AEs will be captured. The Investigator or Research Coordinator will record all AEs on the appropriate SW. The applicable SW will capture the date of onset, severity, duration, outcome and relationship to the Investigational Drug. The Investigator is responsible for determination of severity and relationship to the Investigational Drug.

The Investigator will assess subjects at each study visit for the occurrence of AEs. In order to avoid bias in eliciting AEs, subjects should be asked non-leading questions. All AEs, regardless of severity, reported by the subject or found on examination or laboratory report, must be recorded.

The Investigator or Research Coordinator must report all AEs considered serious in nature and the causality to study drug to the CRO within 24 hours of becoming aware of the AE. Follow-up reports must be submitted to the CRO as additional information becomes available.

All AEs must be followed until resolution or a stable clinical endpoint is reached. All treatments and outcomes of the AE must be recorded.

All AEs and SAEs must be followed until:

- AE is resolved (i.e. return to normal/baseline values)
- AE is declared clinically insignificant
- AE has stabilized
- Subject is lost to follow-up or withdraws consent
- Subject completes study, including required follow-up visits
- Study closure

The Investigator must, following GCP guidelines, continue to treat (or refer subject to an appropriate practitioner for continuing treatment) any AE that remains unresolved after the subject has completed study participation.

9.1 AE Definitions

9.1.1 Adverse Events

Adverse event means any untoward medical occurrence associated with the use of a drug in humans, whether or not considered drug related. AEs may occur at any time during study participation and are not limited to the period of exposure to the Investigational Drug. AEs may include abnormal laboratory findings, medical complications and changes in the subject's condition.

9.1.2 Suspected Adverse Reaction

Suspected adverse reaction means any adverse event for which there is a reasonable possibility that the drug caused the adverse event. 'Reasonable possibility' means there is evidence to suggest a causal relationship between the drug and the adverse event. A suspected adverse reaction implies a lesser degree of certainty about causality than adverse reaction, which means any adverse event caused by a drug.

9.1.3 Unexpected Adverse Event/Reaction

An adverse event or suspected adverse reaction is considered “unexpected” if it is not listed in the investigator brochure or is not listed at the specificity or severity that has been observed; or, if an investigator brochure is not required or available, is not consistent with the risk information described in the general investigational plan. When new adverse event information is received, the Safety Officer and DSMB will be responsible to determine whether the event is “unexpected” for safety reporting purposes

9.1.4 Serious (or Life-threatening) Adverse Events or Adverse Reaction

An adverse event or suspected adverse reaction is considered “serious” if, in the view of **either** the investigator **or** sponsor, it results in any of the following outcomes:

- Leads to death
- Is life threatening, or places the participant at immediate risk of death
- Requires or prolongs inpatient hospitalization
- Results in a significant, persistent or permanent change, impairment, damage or disruption in the patient's body function/structure, physical activities and/or quality of life
- Results in congenital anomaly/birth defect
- Any other serious or important event that may jeopardize the patient and may require medical or surgical intervention (treatment) to prevent one of the other outcomes.

An adverse event or suspected adverse reaction is considered “life-threatening” if, in the view of **either** the investigator **or** sponsor, its occurrence places the patient or subject at immediate risk of death.

Within 24 hours of becoming aware of an SAE, the Investigator must complete the SAE SW (providing as much information as is immediately available) and submit to the CRO. The Investigator must also report all SAE’s to the IRB/REB/ERC, as required by the IRB/REB/ERC.

[Adapted from FDA 21 CFR 312.32(a) Revised as of April 1, 2013].

10 Investigator Responsibilities

10.1 Investigator Qualifications

Investigators shall have proper medical qualifications, training and licensure to perform the clinical duties involved in the trial. Investigators shall have Human Subjects Research Protection or equivalent training. Finally, Investigators shall allocate sufficient time to perform duties involved in this clinical trial, to delegate duties to qualified research staff, to supervise the research team and to maintain the facility qualified for the study.

10.2 IRB/REB/ERC Ethical Approval

This study must have initial and continuing approval (when applicable) from an IRB/REB/ERC responsible for approving clinical studies or from such body responsible for overseeing clinical investigations in the respective country where the investigation is conducted. This can be a local or a central IRB.

Furthermore, no study activities, including pre-screening or screening of subjects may commence until the IRB/REB/ERC approval letter is received by the Sponsor/CRO. A copy of the IRB/REB/ERC approval letter must be filed on-site in the site's regulatory binder. Where appropriate, amendments to the Protocol will be submitted for IRB/REB/ERC review and approval before implementation.

10.3 Protocol Adherence

The Investigator agrees to conduct the study in accordance with this Protocol. Prior to initiating the study, the Investigator must sign and return the Investigator Agreement and the Protocol Signature Page of this Protocol.

An Investigator may not make any changes to the Protocol or study procedures, without first receiving written approval from the Sponsor/CRO and the IRB/REB/ERC, except when necessary to eliminate apparent immediate hazards to a subject.

10.4 Review of Source Documents

The Investigator agrees that the Sponsor/CRO's employees or designees, as well as representatives of FDA, Health Canada and regulatory agencies in the countries where the investigation is conducted will have the right to audit and review pertinent medical records relating to this clinical trial.

10.5 Record of Investigational Drug Inventory

The Investigator will maintain a Medication Accountability Log of all Investigational Drug received, used or returned during this study. The Medication Accountability Log should be available during all monitoring visits. All Investigational Drug not used in this study must either be returned to the Sponsor/CRO at the completion of the study (or earlier at the Sponsor/CRO's request) or with written permission, may be destroyed at the site.

10.6 Data Recording and Record Retention

- All data will be available in source documentation, including SWs for each subject enrolled in the study.
- The Sponsor/CRO will review completed source documentation, including SWs. The Investigator will ensure that subject medical records are made available for review by the Study Monitor and/or regulatory agencies, as required.
- All subject study records are to be maintained in a secure storage facility for at least two years after study completion or for a longer period as required by the local regulations. Subject study records will not be destroyed without authorization from the Sponsor/CRO. This includes the following documentation:
    - SW, ICD, and all study logs and forms
    - Investigational Drug Accountability Logs and Investigational Drug shipment receipts of all products shipped to the site
    - Correspondence with the IRB/REB/ERB, Sponsor/CRO, FDA/Health Canada or other regulatory agency, Study Monitor or other Investigators
    - Study Protocol (all versions, if applicable)
    - Protocol and ICD approvals from the IRB/REB/ERB
    - Clinical Study Agreement and curricula vitae of Investigator(s) and other staff and the Study Delegation Form

10.7 Notification Reporting

The Investigator is responsible for all reporting required as per IRB/REB/Ethical committees.

11 Study Data Reporting and Processing

11.1 Study Data Collection

The SWs are designed to accommodate the specific features of the trial design. The SWs contains all data items specified in this Investigational Plan.

All forms and other study related materials should be submitted according to the schedule reflected in Table 8

Table 8: Schedule for Forms and Reports Submission

Event	SWs & Reports	Submission Schedule
**Screening & Enrollment**	Screening and Enrollment Log	Within 7 days
**All source data**	Entered into eCRF	Within 48 hours, but not later than 14days
**Other**	Progress Notes, Narrative Summaries, Protocol Deviations, Protocol Violations, Study Drop-out, etc.	Within 14 days
**SAEs / Emergency Un-blinding**	SAE, Emergency Un-blinding Forms with relevant records	Within 24 hours

11.2 Site Data Monitoring and Quality Control

Primary data collection based on source-documented hospital chart reviews, SWs and subject interviews will be performed by the Investigator and/or Study Coordinator(s) at each site. Source data will be entered into the eCRF within 48 hours and at latest, within 14 calendar days of data collection.

Data entered into the eCRF will be continuously verified by the Sponsor/CRO. Any deficiencies and/or inconstancies identified will be communicated regularly by electronic requests for clarifications and corrections. In addition, frequent communication between the Study Coordinator(s) and the Study Monitor will take place.

All clinical sites will be monitored periodically by the Sponsor/CRO for Protocol adherence, adherence to applicable SOPs, accuracy of SW completion, GCP and compliance to applicable regulations. Corrective actions will be requested in the case of non-compliance.

In the initial phase of the trial, the Sponsor/CRO will coordinate and host teleconference calls between the Monitor, data management personnel and each site, as necessary, to resolve any problems concerning the Protocol and data collection. Every effort will be made to ensure compliance with the Protocol. In addition, Sponsor/CRO will maintain personal contact with the investigators and staff throughout the study by phone, mail, email and on-site visits.

A Recruitment Status Report generated from the EDC will identify variations in recruitment frequency among sites. The overall recruitment will be evaluated against the targeted recruitment dynamic.

11.3 Subject Coding

Documents sent to the Sponsor/Sponsor's designee will not contain subject names or other directly identifiable patient information. Each subject will be assigned a unique SIC. The SIC will consist of 8 characters in an alphanumeric combination. The site will maintain the link between the SIC and the names. An example is shown below in Table 9.

Table 9: Example of the SIC

Site Code			Connector	Subject Study Number			
**A**	**B**	**C**	**-**	**1**	**2**	**3**	**4**

Personal information may be reviewed for the purpose of verifying data in the SWs. This can be performed by the Monitor, authorized Sponsor/CRO representative, regulatory agencies or quality assurance personnel. Personal medical information will be treated as confidential at all times.

11.4 Data Processing and Quality Control

11.4.1 Electronic Data Capture System

The study will use a specialized EDC called OpenClinica® (Copyright © OpenClinica LLC and collaborators, Waltham, MA, USA, www.OpenClinica.com). The installation of OpenClinica® used in this study is operated by the Methods Core of the AOSNA. OpenClinica® enables 21 CFR Part 11-compliant software solution to store clinical research data.

Conventional data verification sub-routines will be programmed to test entry and logical errors, while all individual (subject-based) eCRF will be linked for cross-reference. Periodic analysis of each data field across cases will be performed in order to examine the expected distributions and to identify outliers for possible mistakes. All research staff that will be responsible for data entry will receive training.

11.4.2 Data Cleaning

All eCRFs will be subjected to initial inspection for omitted data, gross data inconsistencies and timeliness of reporting. Any deficiencies will be resolved using electronic tracking, revision of errant forms at the clinical site and entry of corrected data into eCRF

11.4.3 Data Entry

Data entry will be directly into the eCRF system from source data and will be performed at the investigative sites. Study Monitor will verify the accuracy of data transcription according to specifications in the Monitoring Plan and Data Management Plan.

11.4.4 Data Editing

Each data record is evaluated with electronic intra-form and inter-form edit checking on a regular interval. All errors are then referred to the clinical site for review and correction. Only the Investigator and Study Coordinator are authorized and allowed to edit and modify the data. The Sponsor/CRO does not have access rights to change the stored study data. The OpenClinica®

stores audit records of all changes made to the data, with electronic time and date stamp and electronic signature. Once entered, the data cannot be erased; it can be only edited.

11.4.5 Data Update

The cycle of data edit will be ongoing until all the data is clean. The Sponsor CRO will monitor the clinical site for source documentation verification. If further data entry or source documentation errors are discovered during the site visit, the Investigator or Study Coordinator will be expected to make the appropriate corrections at that time.

11.4.6 Final Data Quality Analyses

All datasets exported for analyses will undergo a final data cleaning procedure using programmed logical routines unique to each exported dataset.

11.4.7 Data Back-up

The OpenClinica® system maintains continuous mirroring back-up system on secure operating servers. Additionally, full back-ups will be performed daily at an off-site location.

11.4.8 Report Generation and Summary Statistics

A customized report is generated for record keeping and subject scheduling, serving as an overview of the current recruitment, follow-up and data processing status.

11.5 Confidentiality and Protection of Study Files

Access to the EDC is protected with an industry strength passcode for each individual user. Passcodes are user-specific and protect confidentiality and the data by allowing variable levels of access. The data transmittal to the EDC database uses approved industry standards for secure data encryption and transmission. Hard copies of SWs are kept in a locked, secure location when not in use. Additional data safety procedures are in place but are not disclosed for safety purposes.

12 Study Management

12.1 Operations Committee

The Operations Committee for the study will be composed of one representative from each qualified participating collaborative organization, Study PI, Study co-PI(s) and the Lead Investigator of the AOSNA Research Network Methods Core. This committee will meet as needed by conference or teleconference to monitor subject recruitment, clinical site progress and Protocol compliance. It will also be responsible for reviewing the final results, determining the methods of presentation and publication and selection of secondary projects and publications.

12.2 AOSpine North America Research Network Methods Core

AOSNA Research Network Methods Core will be responsible for supervising key logistical trial operations, in particular clinical data management, operations of the EDC system and safe release of data.

12.3 Safety Officer

A Safety Officer will be responsible for evaluation of all SAEs with available information to determine whether the event meets the definitions of suspected adverse reaction, serious and unexpected based on the definition provided by 21 CFR 312.21 The Safety officer will provide recommendations for subsequent actions to be taken when it becomes appropriate.

12.4 Data Safety and Monitoring Board (DSMB)

The DSMB serves two functions. First it will review the accumulative clinical data relating to the safety of the investigational treatment and, if needed, recommend to the Sponsor additional safety measures, including stopping the study for the safety reasons. Second, it will review the results of the Interim Statistical Analysis, which may lead to adaptive change in sample size or discontinuation of the study for futility. The operations of the DSMB are described in the DSMB Charter.

12.5 Monitoring

Monitoring activities will be performed both on- and off-site according to GCP guidelines. The Study Monitor (or other Sponsor /CRO representative) will conduct the Site Initiation Visit, periodic site visits and a close-out visit for each site. Some sites may also have a Pre-Investigation Visit.

12.5.1 Pre-Investigation Visits (Site Evaluation Visit)

The Monitor or other Sponsor/CRO representative will ensure that the Investigator clearly understands and accepts the obligations incurred in undertaking this clinical investigation. Prior to the initiation of the clinical investigation, the Monitor or Sponsor/CRO may visit the clinical site to ensure that the Investigator:

- Understands the nature of the Protocol
- Understands the requirements for an adequate and well-controlled study

- Understands and accepts the obligation to conduct the clinical investigation in accordance with applicable sections of Title 21 of CFR and/or any other applicable regulation
- Understands and accepts the obligation to obtain ICD in accordance with 21 CFR Part 56
- Understands and accepts the obligation to obtain IRB/REB/ECR approval before the investigation may be initiated and to further ensure a continuing review of the study by the IRB in accordance with CFR 21 Part 56, and to keep the Sponsor/CRO informed of such IRB approval and subsequent IRB actions concerning the study
- Has access to an adequate number of suitable subjects to conduct the investigation
- Has adequate facilities and staff for conducting the clinical investigation
- Has sufficient time from other obligations to carry out the responsibilities to which the investigator is committed by applicable regulations

12.5.2 Initiation Visit

The monitor will visit each study site at least once before the beginning of the clinical trial. No Screening or other visits may be performed before Site Activation.

12.5.3 Periodic Visits

The Sponsor/CRO will assure throughout the clinical investigation that the Investigator's obligations, as set forth in applicable regulations and in GCP guidelines, are being fulfilled and that the facilities used in the clinical investigation continue to be acceptable. Personal contact between the Study Monitor and the Investigator will be maintained throughout the clinical investigation. The Study Monitor will visit each site frequently enough to ensure that:

- Facilities/staff used by the Investigator continue to be adequate for purposes of the study
- The study Protocol is being followed
- Protocol Amendments (if applicable) have been reported to and approved by the IRB/REB/ECR
- Accurate, complete and current records are maintained
- Accurate, complete and timely reports are made to Sponsor/CRO and the IRB/REB/ECR
- The Investigator is carrying out the agreed upon activities and has not delegated them to other unspecified staff

During periodic visits the Monitor will compare a representative number of subject records, SWs and other supporting documents with the Investigator's reports and eCRFs to determine that: The information recorded is complete, accurate and legible. There are no omissions of specific data elements

- Missing visits or examinations are noted and an acceptable rationale is reported
- Subjects failing to complete the study and the reason for each failure are noted
  ICD has been documented in accordance with 21 CFR Parts 50 and 56

As guidance, the first periodic visits will be performed after the enrollment of the first one or two subjects. Subsequent Monitor visits will take place about every 180 days; however visit frequency may be altered based on site performance and special or unforeseen circumstances.

12.5.4 Close-out Visit

The Monitor must conduct a close-out visit at each participating investigative site. The Monitor will conduct the close-out visit when:

- All participants enrolled at the site have completed study-related activities; and
- A reasonable amount of time has been given to the site to enter data into OpenClinica® and to resolve outstanding discrepancies.

12.5.5 Record of On-Site Visits

The Monitor will maintain a record of the findings, conclusions and action taken in order to correct deficiencies for each on-site visit. The record will include the date of the visit, the name and address of the Investigator, any findings or conclusions and any actions taken to correct deficiencies.

12.5.6 Direct Access to Source Documentation

The Study Monitor must be allowed direct access to source documentation for the purpose of verifying that the data in the eCRF are consistent with the original source data. Findings from this review of SWs and source documents will be discussed with investigational staff. The Sponsor/CRO expects that the relevant investigational staff, including the Investigator and Study Coordinator(s), will be available, the source documents will be accessible and a suitable environment will be provided for review of study-related documents during monitoring visits. The monitor will meet with the Investigator to provide feedback on the conduct of the study.

13 Document Control

13.1 Responsibility

Methods Core of the AOSpine North America Research Network is responsible for document control, Protocol versions and for controlling site or topic-specific Protocol amendments. Protocol and Protocol amendments will be signed off on by the Sponsor.

13.2 Protocol Amendments

No changes to the IRB/REB/ERC -approved Protocol are allowed except when removing immediate threats for patient safety. Any change to the Protocol made to protect the life and well-being of the enrolled Subjects must be reported to the Sponsor/CRO within 5 days.

13.3 Protocol Deviations

A Protocol deviation is a non-adherence to the Protocol that does not involve Inclusion/Exclusion criteria, primary efficacy variable and/or GCP guidelines. Protocol deviations are minor and do not impact the study in a major way. Protocol deviations are to be reported to the Sponsor/CRO.

13.4 Protocol Violations

A Protocol violation is any significant divergence from the Protocol on the part of the subject, Investigator, other site staff or Sponsor/CRO that affects Inclusion/Exclusion criteria, primary efficacy variable or GCP guidelines. The Sponsor/CRO will be notified immediately. Violations will be recorded at the site and reported to the IRB as required. Protocol Violations will be reported to the Sponsor/CRO. The Investigator may implement a deviation from, or a change in, the Protocol to eliminate an immediate hazard(s) to trial subjects without prior IRB/REB/ERC approval. As soon as possible, the implemented deviation or change, the reasons for it, and, if appropriate the proposed Protocol amendment(s) should be submitted:

a) To the IRB/REB/ERC for review and approval/favorable opinion;
b) To the Sponsor/CRO for agreement and, if required;
c) To the regulatory authority(ies).

13.5 Record Retention

In compliance with ICH/GCP guidelines, the Investigator/Institution will maintain all SWs and source documents that support the data collected from each subject, as well as all study documents in ICH/GCP Section 8: Essential Documents for the Conduct of a Clinical Trial, and all study documentation as specified by the applicable regulatory requirement(s).

Clinical research records shall be stored in a manner that ensures privacy, confidentiality, security and accessibility of the records both during and after the conduct of clinical trial. The Investigator/Institution will take measures to prevent accidental or premature destruction of those documents. Essential documents must be retained for at least 2 years after study completion.

If the responsible investigator retires, relocates, or withdraws from responsibility of keeping the study records, custody must be transferred to a person who will accept the responsibility. The

Sponsor/CRO must be notified in writing of the name and address of the new custodian. Under no circumstance shall the Investigator relocate or dispose of any study documents before having obtained written approval from the Sponsor/CRO.

If it becomes necessary for the Sponsor/CRO or appropriate regulatory authority to review any documentation relating to the study, the Investigator must permit access to such reports.

14 Appendices

14.1 Appendix A: Riluzole Drug Monograph

14.2 Appendix B: Scales and Questionnaires

14.3 Site and Topic Specific Protocol Addendums

15 References

1. Ackery, A., C. Tator, and A. Krassioukov, *A global perspective on spinal cord injury epidemiology.* J Neurotrauma, 2004. **21**: p. 1355-1370.
2. Wyndaele, M. and J. Wyndaele, *Incidence, prevalence, and epidemiology of spinal cord injury: what learns a worldwide literature survey?* Spinal Cord, 2006. **44**: p. 523-529.
3. Sekhon, L. and M. Fehlings, *Epidemiology,demographics, and pathophysiology of acute spinal cord injury.* Spine, 2001. **26**: p. S1-12.
4. DeVivo, M., *Causes and costs of spinal cord injury in the United States.* Spinal Cord, 1997. **35**: p. 809-813.
5. Foundation, C.a.D.R. *Paralysis Facts and Figures*. 2011 July 12, 2011]; Available from: http://www.christopherreeve.org/site/c.mtKZKgMWKwG/b.5184189/k.5587/Paralysis_Facts__Figures.htm.
6. Rowland, J., et al., *Current status of acute spinal cord injury pathophysiology and emerging therapies: promise on the horizon.* Neurosurgical Focus, 2008. **25**(5): p. E2.
7. Fehlings, M. and L. Sekhon, eds. *Cellular, ionic and biomolecular mechanisms of the injury process*. Contemporary Management of Spinal Cord Injury: From Impact to Rehabilitation, ed. B. E and T. CH. 2000, American Association of Neurologic Surgeons: Chicago. 33-50.
8. Tator, C. and M. Fehlings, *Review of the secondary injury theory of acute spinal cord trauma with emphasis on vascular mechanisms.* J Nerosurg, 1991. **75**: p. 15-26.
9. Amar, A. and M. Levy, *Pathogenesis and pharmacological strategies for mitigating secondary damage in acute spinal cord injury.* Neurosurgery, 1999. **44**: p. 1027-1039.
10. Braughler, J., L. Duncan, and R. Chase, *Interaction of lipid peroxidation and calcium in the pathogenesis of neuronal injury.* Cent Nerv Syst Trauma, 1985. **2**: p. 269-283.
11. Tator, C., *Vascular effects and blood flow in acute spinal cord injuries.* J Neurosurgical Sci, 1984. **28**: p. 115-119.
12. Wallace, M. and C. Tator, *Successful improvement of blood pressure, cardiac output, and spinal cord blood flow after experimental spinal cord injury.* Neurosurgery, 1987. **20**: p. 710-714.
13. Rothman, S. and J. Olney, *Glutamate and the pathophysiology of hypoxic-ischemic brain damage.* Ann Neurology, 1986. **19**: p. 105-111.
14. Hawryluk, G., et al., *Protection and repair of the injured spinal cord: a review of completed, ongoing, and planned clinical trials for acute spinal cord injury.* Neurosurgical Focus, 2008. **25**: p. E14.
15. Hadley, M.N., et al., *Guidelines for the management of acute cervical spine and spinal cord injuries.* Clin Neurosurg, 2002. **49**: p. 407-98.
16. Bracken, M.B., et al., *Administration of methylprednisolone for 24 or 48 hours or tirilazad mesylate for 48 hours in the treatment of acute spinal cord injury. Results of the Third National Acute Spinal Cord Injury Randomized Controlled Trial. National Acute Spinal Cord Injury Study.* JAMA, 1997. **277**(20): p. 1597-604.
17. Bracken, M.B., et al., *A randomized, controlled trial of methylprednisolone or naloxone in the treatment of acute spinal-cord injury. Results of the Second National Acute Spinal Cord Injury Study.* N Engl J Med, 1990. **322**(20): p. 1405-11.
18. Geisler, F.H., et al., *The Sygen multicenter acute spinal cord injury study.* Spine (Phila Pa 1976), 2001. **26**(24 Suppl): p. S87-98.

19. Fehlings, M.G., et al., *Early versus delayed decompression for traumatic cervical spinal cord injury: results of the Surgical Timing in Acute Spinal Cord Injury Study (STASCIS).* PLoS One, 2012. **7**(2): p. e32037.
20. Le Liboux, A., et al., *Single- and multiple-dose pharmacokinetics of riluzole in white subjects.* J Clin Pharmacol, 1997. **37**(9): p. 820-7.
21. Le Liboux, A., et al., *A comparison of the pharmacokinetics and tolerability of riluzole after repeat dose administration in healthy elderly and young volunteers.* J Clin Pharmacol, 1999. **39**(5): p. 480-6.
22. Abbara, C., et al., *Riluzole pharmacokinetics in young patients with spinal muscular atrophy.* Br J Clin Pharmacol, 2011. **71**(3): p. 403-10.
23. Groeneveld, G.J., et al., *An association study of riluzole serum concentration and survival and disease progression in patients with ALS.* Clin Pharmacol Ther, 2008. **83**(5): p. 718-22.
24. Lacomblez, L., et al., *Dose-ranging study of riluzole in amyotrophic lateral sclerosis. Amyotrophic Lateral Sclerosis/Riluzole Study Group II.* Lancet, 1996. **347**(9013): p. 1425-31.
25. Bensimon, G., L. Lacomblez, and V. Meininger, *A controlled trial of riluzole in amyotrophic lateral sclerosis. ALS/Riluzole Study Group.* N Engl J Med, 1994. **330**(9): p. 585-91.
26. Bensimon, G., et al., *A study of riluzole in the treatment of advanced stage or elderly patients with amyotrophic lateral sclerosis.* J Neurol, 2002. **249**(5): p. 609-15.
27. Yanagisawa, N., et al., *Efficacy and safety of riluzole in patients with amyotrophic lateral sclerosis: double-blind placebo-controlled study in Japan.* Igakuno Ayumi, 1997. **182**: p. 851-866.
28. Hugon, J., *Riluzole and ALS therapy.* Wien Med Wochenschr, 1996. **146**(9-10): p. 185-7.
29. Miller, R., et al., *Riluzole for amyotrophic lateral sclerosis (ALS)/motor neuron disease (MND)*, in *Cochrane Database of Systematic Reviews*2009.
30. Bensimon, G. and A. Doble, *The tolerability of riluzole in the treatment of patients with amyotrophic lateral sclerosis.* Expert Opin Drug Saf, 2004. **3**(6): p. 525-34.
31. Landwehrmeyer, G.B., et al., *Riluzole in Huntington's disease: a 3-year, randomized controlled study.* Ann Neurol, 2007. **62**(3): p. 262-72.
32. Tator, C. and I. Koyanagi, *Secondary injury mechanisms of spinal cord trauma: a novel therapeutic approach for the management of secondary pathophysiology with the sodium channel blocker riluzole.* J Neurosurg, 1997. **86**: p. 483-492.
33. Fehlings, M. and G. Schwartz, *Evaluation of the neuroprotective effects of sodium channel blockers after spinal cord injury: improved behavioral and neuroanatomical recovery with riluzole.* J Neurosurg, 2001. **94**: p. 245-256.
34. Schwartz, G. and M. Fehlings, *Secondary injury mechanisms of spinal cord trauma: a novel therapeutic approach for the management of secondary pathophysiology with the sodium channel blocker riluzole.* Prog Brain Res, 2002. **137**: p. 177-190.
35. Heurteaux, C., et al., *Deletion of the background potassium channel TREK-1 results in a depression-resistant phenotype.* Nat Neurosci, 2006. **9**(9): p. 1134-41.
36. Ates, O., et al., *Comparative neuroprotective effect of sodium channel blockers after experimental spinal cord injury.* J Clin Neurosci, 2007. **14**(7): p. 658-65.
37. Lang-Lazdunski, L., et al., *Riluzole prevents ischemic spinal cord injury caused by aortic crossclamping.* J Thorac Cardiovasc Surg, 1999. **117**(5): p. 881-9.
38. Schwartz, G. and M.G. Fehlings, *Evaluation of the neuroprotective effects of sodium channel blockers after spinal cord injury: improved behavioral and neuroanatomical recovery with riluzole.* J Neurosurg, 2001. **94**(2 Suppl): p. 245-56.
39. Jorgensen, M. and N. Diemer, *Selective neuron loss after cerebral ischemia in the rat: possible role of transmitter glutamate.* Acta Neurol Scand, 1982. **66**: p. 536-546.

40. Agrawal, S.K., R. Nashmi, and M.G. Fehlings, *Role of L- and N-type calcium channels in the pathophysiology of traumatic spinal cord white matter injury.* Neuroscience, 2000. **99**(1): p. 179-88.
41. Chow, D.S., et al., *Pharmacology of riluzole in acute spinal cord injury.* J Neurosurg Spine, 2012. **17**(1 Suppl): p. 129-40.
42. Groeneveld, G.J., et al., *Riluzole serum concentrations in patients with ALS: associations with side effects and symptoms.* Neurology, 2003. **61**(8): p. 1141-3.
43. Grossman, R.G., et al., *Incidence and severity of acute complications after spinal cord injury.* J Neurosurg Spine, 2012. **17**(1 Suppl): p. 119-28.
44. Vertiz-Hernandez, A., et al., *L-arginine reverses alterations in drug disposition induced by spinal cord injury by increasing hepatic blood flow.* J Neurotrauma, 2007. **24**(12): p. 1855-62.
45. Mestre, H., et al., *Spinal cord injury sequelae alter drug pharmacokinetics: an overview.* Spinal Cord, 2011. **49**(9): p. 955-60.
46. *Guidance for Industry and Investigators. Safety Reporting Requirements for INDs and BA/BE Studies* U.S.D.o.H.a.H. Services, Editor 2012.
