# Supplementary material for: Safety and Efficacy of Riluzole in Acute Spinal Cord Injury Study (RISCIS): A Multi-Center, Randomized, Placebo-Controlled, Double-Blinded Trial
Source: J Neurotrauma. 2023 Aug 23;40(17-18):1878–88. doi: 10.1089/neu.2023.0163 (PMC10460693; doi:10.1089/neu.2023.0163)
Supplement: Supplemental data [file 4RISCISSAPv1.pdf]

RISCIS

**Author**	Sreedevi Menon
**Reviewer**	Liping Sun
**Approved by**	Branko Kopjar
**Approval Date**	
**Document Number**	RISCIS-STAT-SAP
**Version Number**	1.0

**Treatment Groups**	Experimental: Riluzole 100 mg BID first 24 hours followed by 50 mg BID for 13 days<br>Control: Placebo
**Protocol Number**	SPN-12-001
**Protocol Title**	A Multi-Center, Randomized, Placebo Controlled, Double-Blinded, Trial of Efficacy and Safety of Riluzole in Acute Spinal Cord Injury (RISCIS)
**Sponsor**	AO Spine North America
**Contract Research Organisation**	Nor Consult, LLC

**Revision Chronology**		
**Version**	**Version Date**	**Reason for change**
0.1	22nd January, 2014	Draft initial version
1.0	31st January 2019	Update to a few sections

**Approved By**	Branko Kopjar, MD, PhD
**Signature**	
**Date**	5th February 2019

1. Table of Contents

1. Table of Contents 2
2. List of Abbreviations 3
3. Background 4
4. Study Objective 4
5. Study Design 5
6. Sample Size Calculations 5
7. Analysis Samples/Populations 6
8. Definition 6
9. Handling of Data 7
9.1. Missing values imputations for ISNCSCIMS, SF-36v2, EQ-5D, SCIM, GRASSP and NRS 7
9.2. Safety endpoints 8
10. Planned Statistical Analysis 8
10.1. Analysis Conventions 8
10.2. Interim Analysis 9
10.3. Descriptive Methods 11
10.4. Analyses of the Patient Populations 13
10.5. Subgroup analysis 14
10.6. Primary Analysis 14
10.6.1. Study success 15
10.7. Secondary Analysis 15
10.8. Other Endpoints 15
10.9. Safety Analysis and/or further analysis 16
11. Listings, Tables and Figures 17
12. Software 18

2. List of Abbreviations

AE	Adverse Events
ASIA	American Spinal Injury Association
ASIAMS	American Spinal Injury Association Motor Score
BID	Twice a day (bis in die)
BMI	Body Mass Index
CC	Completed Cases
CRO	Contract Research Organization
CRF	Case Report Form
DSMB	Data Safety and Monitoring Board
GRASSP	Graded Redefined Assessment of Strength Sensibility and Prehension
ICD	Informed Consent Document
ISNCSCI	International Standards for Classification of Spinal Cord Injury
ISNCSCIMS	ISNCSCI Motor Score
ITT	Intent-to-treat
MAR	Missing at Random
MCAR	Missing Completely at Random
MCS	Mental Composite Score
MedDRA	Medical Dictionary for Regulatory Activities
mITT	Modified Intent-To-Treat
MH	Medical History
MRI	Magnetic resonance imaging
NRS	Numeric Rating Scale
PCS	Physical Component Score
PP	Per-protocol
SAE	Serious Adverse Event
SAP	Statistical Analysis Plan
SCI	Spinal Cord Injury
SCIM	Spinal Cord Independence Measure
SD	Standard Deviation
SF36	Short Form 36
SSR	Sample Size Re-estimation
STL	Statistical Tables and Listings
SW	Software
TEAE	Treatment-Emergent Adverse Event
WHODD	World Health Organization Drug Dictionary

3. Background

The annual incidence of traumatic spinal cord injury (SCI) varies depending on the region considered, with international estimates ranging from 10 to 85 per million populations. At present there are over one million people living with SCI in North America, with annual costs for the acute treatment and chronic care of these patients totaling 4 billion dollars USD. In spite of the immense impact of SCI at a personal and societal level, a highly effective and safe pharmacologic treatment for SCI, shown to improve neurological and functional outcomes, remains absent.

The final degree of neurological tissue destruction that occurs after traumatic SCI is a product of both primary and secondary injury mechanisms. The primary mechanical injury to the cord initiates a post-lesion signaling cascade of deleterious down-stream events, known collectively as secondary injury mechanisms. These secondary injury mechanisms include ischemia, interstitial and cellular ionic imbalance, free radical formation, glutamatergic excitotoxicity, lipid peroxidation and generation of arachidonic acid metabolites. Although little can be done from a therapeutic standpoint to correct damage sustained during the primary injury, by mitigating the evolution of secondary injury events, there is opportunity to preserve remnant viable neurological tissue and hence optimize outcomes.

Clinical guidelines for the management of SCI have been established and are widely accepted by physicians who treat patients with SCI. These guidelines include restoration of spinal stability, decompression of the spinal cord and cardiopulmonary and metabolic support of the patient. However, beyond supportive care, there are no medical or surgical treatments that have been clearly demonstrated to improve functional outcome in human SCI. Clinical trials with methylprednisolone (NASCIS II and III) and GM-1 ganglioside have provided suggestive but unequivocal evidence of benefit. A recent prospective, multi-center study has suggested that early decompression, within the first 24 hours after injury, is associated with better neurological outcomes than late surgery.

At present, there is no specific pharmacological therapy that is given uniformly to all patients with traumatic SCI. Hence, it is ethically justifiable to use placebo as control in the trial of riluzole in the context of SCI.

4. Study Objective

The aim of this study is to evaluate efficacy and safety of riluzole in the treatment of patients with acute SCI. The primary objective is to evaluate the superiority of riluzole, at a dose of 100mg BID for the first 24 hours followed by 50 mg BID for the following 13 days after injury, as compared to placebo, in change between 180 days and baseline in motor outcomes as measured by International Standards for Neurological Classification of Spinal Cord Injury Examination (ISNCSCI) Motor Score, in patients with acute traumatic SCI, presenting to the hospital less than 12 hours after injury. Secondary objectives are to evaluate the effects of riluzole on overall neurologic recovery, sensory recovery, functional outcomes, quality of life outcomes, health utilities, mortality, and adverse events. The working hypothesis is that the riluzole treated subjects will experience superior motor, sensory, functional, and quality of life outcomes as compared to those receiving placebo, with an acceptable safety profile.

Study success is defined as follows.

Investigational treatment (riluzole) is superior to placebo. Study success is achieved if the one-sided null hypothesis of no superiority of riluzole group in $\Delta ISNCSCIMS_{180\text{-}b}$ is rejected.

5. Study Design

This is an international, multi-center, prospective, double-blinded, randomized, placebo controlled Phase II/III clinical trial to evaluate if riluzole at a dose of 100 mg BID for the first 24 hours followed by 50 mg BID for 13 days is superior to placebo in subjects with acute traumatic SCI.

The study will involve up to 35 investigational sites and enroll 351 subjects (includes 10% allowance for attrition). A randomization ratio of one riluzole subject to one placebo-controlled subject (1:1) will be utilized. The sample size may change during the interim analysis due to the adaptive statistical design.

6. Sample Size Calculations

Sample size has been calculated to provide 90% power in testing the primary superiority hypothesis. The estimate has been calculated by PROC SEQDESIGN for SAS/STAT. Using the data from the performed STASCIS study (data on file with the Sponsor); the following empirical estimates have been obtained for the primary outcome parameters.

Endpoint	Standard Deviation
Δ $ISNCSCIMS_{180\text{-}b}$	24.08

Sample size estimate Δ $ISNCSCIMS_{180\text{-}b}$

Plan ID	Parameter
**Type of the hypothesis**	1-sided
**Type I Error (α)**	0.025
**Power ($1 - \beta$)**	0.90
**Randomization Ratio (Investigational vs. Control)**	1:1
**Planned Number of Interim Looks**	2
**Spacing of Looks**	60%, 100%
**Hypothesis to be Rejected**	H_0 or H_1 (binding)
**Boundary Family**	Published Function
**Boundary to Reject H_0**	Ian DeMets (resembles O'Brien-Fleming boundary)
**Boundary to Reject H_1**	Gamma (-1)
**Difference of Means Assuming H_1**	9
**Standard Deviation (σ)**	24.08
**Sample Size**	316 (158 per arm)

Under the above assumptions, the estimated sample size is 316 evaluable subjects. (158 in the Riluzole arm and 158 in the control arm). In order to account for loss of power due to loss of follow-up and possible adjustments for baseline factors the sample size will be increased by 10% to 351 enrolled subjects.

7. Analysis Samples/Populations

Geographic Cohort

This is an international study with sites in Australia, Canada and the United States. Further global expansion is possible. All sites follow the same protocol, the sponsor will adequately and in the same fashion monitor all sites, and the data gathering and the validation mechanism will be the same across all sites. Data gathered in all sites will be treated as one cohort.

Intent-to-Treat (ITT) population

Consenting subjects who are randomized to a study arm and receive at least one dose of the investigational product (i.e. enrolled subjects) will be included in the Intent-To-Treat (ITT) analysis population, regardless of the actual treatment received. Screen failures will be recorded on the Screening Source Worksheet and will not be included in the ITT group. Values for the subjects who do not have the 180-day end point will be imputed to create a complete ITT population.

Completed Cases (CC) Population

The Completed Cases (CC) population is defined as all enrolled subjects and eligible patients who receive at least one dose of study-directed treatment (medication) and had 180 days of follow-up defined as any efficacy measurement taken at this visit.

Per-Protocol (PP) Population

The per-protocol population (PP) population is a subset of CC population and it includes all enrolled patients entirely consistent with the protocol who received at least 80% of study directed treatment and had 180 days of follow-up. Any visits where protocol violations occurred will be reviewed for possible exclusion from PP population.

Modified Intent-to-Treat (mITT)

The modified Intent-To-Treat (mITT) population is defined as all consenting subjects who receive at least one dose of study-directed treatment and have any follow-up. The mITT population will be used for safety analyses.

8. Definition

- **Age:** The age will be calculated as the exact number of days between the date of birth and date of informed consent converted to years (i.e. difference in days/365.25).
- **Body Mass Index (BMI):** BMI is obtained by dividing the weight (kg) at by the square of height (m) at screening/ enrollment i.e. weight (kg)/ height^2 (m).
- **Baseline:** Screening/enrollment visit is considered as the baseline visit for all parameters.
- **Scores:** The global scores or scores under each dimension of the questionnaires under consideration will be determined per specific scoring instructions for each questionnaire.
- **Change in score between baseline and follow up visits** for the assessments will be considered as the difference between the follow up visit and the baseline score (follow up score – baseline score). However, based on the description in the analysis section below, the absolute change will be considered during analysis.

- **Prior/ Concomitant Medication:** All medications with start and end date prior to the date of first drug intake will be considered as prior medications. All other medications will be considered as concomitant medications.

- **Treatment-Emergent AEs:** All AEs with start date on or after the date of first drug intake will be considered as treatment emergent AEs.

- **Serious Adverse Events:** Serious adverse events are defined as the adverse events which meet the definition of serious and are marked as such in the AE form.

- **Screen failure:** Screen failure is defined as a subject who has signed the ICD but does not meet the eligibility criteria, was not enrolled by the Investigator for any reason or withdraws consent for any reason prior to enrollment. A subject may be considered a screen failure any time prior to enrollment.

- **Enrolled Subject:** A subject is considered to be enrolled into the trial if they have been randomized.

- **Withdrawn (Discontinued) Subject:** A subject who does not complete the study. This will be recorded on the Subject Withdrawal Form.

- **Lost to Follow-up:** A subject will be considered lost to follow-up if he/she does not appear for the scheduled study visit and study personnel are unable to contact the subject.

- **Completed Subject:** A subject will be considered as having completed the study if he/she has completed all assessments through the 180-day visit. The 365-day visit will be used to determine the possible sustainability of the effect. The last subject will not be followed up past the 180-day visit.

9. Handling of Data

9.1. Missing values imputations for ISNCSCIMS, SF-36v2, EQ-5D, SCIM, GRASSP and NRS

If the 6-month score is missing for ISNCSCIMS, SF-36v2, EQ-5D, SCIM, GRASSP and NRS the multiple imputation procedure available in SAS (SAS PROC MI) will be used as a robust approach to handle missing values. The basis for the multiple imputation technique has been described by Rubin D, 1987 and Schafer J. The statistical parameters for the imputed samples will be estimated by SAS PROC MIANALYZE. The MIANALYZE procedure uses the method described by Barnard, J. and D. B. Rubin, 1999.

Prior to imputation missing cases will be classified into Missing at Random (MAR)/ Missing Completely at Random (MCAR) and Not-Missing-at-Random (nMAR). Only the MAR/MCAR will be imputed using the SAS PROC MI. NMAR cases will be treated as:

(i) excluded from the analysis and
(ii) imputed using the specific value for each case based on the circumstances for missing values.

Ten imputed samples will be created. The seed point for the imputation will be the value "7041776" (the date of the U.S. Declaration of the Independence) to assure that seed point does not bias the imputation results. The imputations will be performed using the Markov Chain Model and the full imputation and multiple chains.

The imputation will target the entire missing score value and not the individual questions (items) composing the score. The mandatory variables for the imputation model will include study arm and available follow-up score values. Next, other baseline variables to be selected for the imputation model will be screened by applying the following methodology, which is similar to the method described by Hosmer and Lemeshow (2000). First, Pearson correlation coefficients "r" and the corresponding p values will be calculated for the H_0:r=0 between the predictor candidate variables and the values of the missing scores at 6 months (dependent variables). If the predictor candidate has a p value >= .2, it will not be included for the final imputation model. The results of screening will be tabulated for review. All predictors that have p value ≤ .2 will be included in the final model. The predictors that are measured on the nominal scale will be re-coded into dummy variables. The study arm will be forced into the imputation model regardless of the p-value.

Imputations will be performed on ITT population.

9.2. Safety endpoints

Safety endpoints will not be imputed. Rather, all available follow-ups will be used to describe safety events.

10. Planned Statistical Analysis

This section describes the various statistical analyses that are planned to be performed for this study. Primary analysis of primary , secondary and other efficacy endpoints will be performed on the ITT population. Safety will be analyzed on mITT population.

Secondary analyses of primary, secondary and other efficacy endpoints will be performed on the CC and PP populations.

Characteristics of the study variables will be evaluated to validate assumptions needed for the statistical test procedures. Histograms will be studied and tests performed to determine consistency of variables with basic assumptions. For continuous variables, the assumption of normality and equality of variance will be tested. For other variables, the numerical density of the observations will be evaluated to determine appropriateness of chosen categorical procedures. These evaluations include limitations of tabulated cell frequencies on the use of Chi-square tests.

Means, standard deviations, medians and ranges will be reported for all continuous variables. Dichotomous variables will be reported as percentages and the numerator and denominator will be reported and defined. Adverse events will be reported by count and percentage.

10.1. Analysis Conventions

The following conventions will be applied to all data presentations and analyses

- ❑ Tables, listings and figures will be presented in landscape orientation
- ❑ Tables and figures will be presented for analysis populations as defined in the titles of the tables
- ❑ Listings will be presented for the populations
- ❑ All the displays will be in Tahoma 8pt font.

- All tables and listings will have footer sections which will contain the name of the program.
- Summary tables and figures will contain footnotes in the body of the title that reference any data listings or tables associated with the table or figure (e.g. Reference: Listing 16.X.XX).
- Additional footnotes may be added as per the STL shells.
- The outputs have to be created as per the shells that will be provided along with this analysis plan.
- Guidelines provided in the STL shells under "Programming notes" should be referred to by the programmers for clarity on shells.
- All means and medians will be formatted to one more decimal place than the measured value. Standard deviation values will be formatted to two more decimal places than the measured value. Minimum and maximum values will be presented with the same number of decimal places as the measured value.
- p-values and confidence intervals will be formatted to three decimal places.
- The number and percentage of responses will be presented in the form XX (XX.X %) where the percentage will be in parentheses.
- All summary tables will include the total number of subjects in the population that is being analyzed. This information will be given in the column headings in each table.
- All listings will be ordered by treatment group and subject number.
- Date variables will be formatted as DDMMMYYYY for presentation.
- SAS® Version 9.3 will be used for all data analysis

10.2. Interim Analysis

The interim analysis will be performed at about 60% of the initial sample. The sample for the interim analysis will be chosen on a sequential enrollment basis (i.e. all subjects being randomized until the specific study date, which gives approximately 60% of the sample, will be included in the interim analysis).

The interim analysis will test single superiority hypothesis. In addition, the binding futility hypothesis will be tested. The alpha spending function will be Ian DeMets (resembles O'Brien-Fleming boundary) and the beta spending function (futility) as gamma (-1).

The threshold values for hypotheses rejection at the time of the interim analysis depend on the actual sample size. For the illustration purposes, the sample of 190 subjects at interim analysis will have the following threshold values:

RISCIS

Table 10.2.1. Nominal critical points for rejection of H_0 and H_1 at the interim analysis (N=190)

			Nominal Critical Point			
Look	Cumul.	Info	Reject H_0		Reject H_1	
#	Accrual	Fract.	Lower	Upper	Lower	Upper
1	190	0.600	2.669		0.912	

The actual nominal critical points will depend upon the sample size involved at the time of the analysis. The input information will be prepared by the SAS software.

The testing will be performed using the validated SAS Adaptive design routines.

Stopping rules

The study will stop at the interim analysis if:

1. If the criteria for the study statistical success was reached
2. If the test value reaches futility boundary the Sponsor may decide to stop the study.

If the study does not reach stopping criteria at the interim analysis, the study will proceed into the next (final) data look. Prior to that, the adaptive change will be performed, if indicated.

The interim analysis will be performed by an independent blinded statistician and reviewed by the Data Safety and Monitoring Board (DSMB). The statistician will be blinded using a dummy treatment allocation. The DSMB will advise the Sponsor of the status to the extent needed.

Adaptive Change

The adaptive sample size re-estimation (SSR) after the first interim analysis is pre-planned per the study protocol. If the study does not reach the stopping rules at the interim analysis, the sample size re-estimation will be performed using the conditional power approach. The actual conditional power function to be used will be decided upon after simulations at which time this SAP will be updated. This will occur before the first interim analysis. The following conditions will apply for the adaptive change:

1. If the sample size re-adjustment shows a need for upwards re-adjustment, the sample size will be adjusted up to 540 cases maximum. The 540 cases is equivalent to about .25 Cohen's standardized difference or approximately 6 absolute points on a 0—100 ASIAMS. This number represents lowest possible difference that is possibly still clinically interesting.

2. Maintain overall unconditional one-sided alpha level at pre-determined level of .025;

3. Keep overall conditional power at least 60%;

The sample size re-adjustment will be performed using the East ver. 5.4 (Cytel, Inc. Cambridge, MA) or later version that supports adaptive design change for the superiority trial of normally distributed variables, or a set of SAS routines for adaptive change using CRO internal SAS macros.

Caution is needed in interpreting the results of the adaptive change. Due to the modest sample size at the time of the interim analysis, small variations in the outcomes may result in substantial differences in the sample size suggestions. The sample size adjustment depends on the observed difference at the time of the interim analysis as well as the overall variance.

If the analysis indicates a need for adaptive change, the range of values under different scenarios and a continuum of conditional probabilities will be presented to choose a reasonable scenario.

10.3. Descriptive Methods

All the data collected during the study will be presented as tables or listings. The below section describes the outputs that will be generated against the data collected. The outputs will be generated for the enrolled subjects.

- **Inclusion/Exclusion criteria**

The information pertaining to the inclusion and exclusion criteria will be listed.

- **Screening Information**

All screening information will be listed and may be tabulated.

- **Demography**

Demographic variables include date of birth, age, gender, height, weight at enrollment, BMI, Race and Ethnicity. All the variables will be listed in the demography listing.

In addition, descriptive statistics for relevant variables including mean, standard deviation, median, minimum and maximum will be presented in tabular format for enrolled subjects with respect to the treatment groups. The age will be calculated as mentioned in section 8 of this document.

- **Socioeconomic Status**

All the parameters collected as part of the socio economic status namely marital status, employment status, household income, education and insurance will be listed and the number and percentage of patients in each category of these socio economic parameters and treatment group will be tabulated.

- **Pregnancy Test Results**

The pregnancy test results will be listed.

- **Charlson Comorbidity Index**

The conditions reported as part of the Charlson Comorbidity Index will be listed.

- **Vital Signs**

The vital sign parameters will be listed. Descriptive statistics for parameters will be presented by visit and treatment group. The descriptive statistics for the change in vital signs over the visits with respect to baseline will also be presented.

- **Medical History**

Medical History terms will be coded using MedDRA version 14.1. A listing will be created to present the medical history information collected. A table will be created to present the count and percentage of patients with diagnosis/procedure falling under each body system and preferred term and treatment group.

RISCIS

❑ Spine Trauma Injury Data

The spine trauma injury data will be listed.

❑ Injury Severity Score

The injury severity score will be listed and the descriptive statistics for the score by region and overall score will be tabulated by treatment group.

❑ Randomization

The details pertaining to the randomization information will be listed. The data will be tabulated in the Patient Disposition table.

❑ Health Behavior

The health behavior data will be listed.

❑ Surgery Information

The surgery information data will be listed.

❑ Discharge Information

The discharge information along with the physical and occupational therapy data will be listed.

❑ MRI Reporting Log

MRI reporting log data will be listed.

❑ Investigational Drug Compliance

The data pertaining to the investigational drug compliance will be listed. The treatment compliance will be further tabulated by treatment group.

❑ Analysis Population

The information pertaining to the analysis population flag will be presented for each patient as a listing. The data will be tabulated in the Patient Disposition table.

❑ Protocol Deviations

Protocol deviations will be classified into minor or major. The patients reporting protocol deviations will be listed along with the deviation and its classification.
The subjects with major protocol violations will be tabulated by violation type and affected visits and treatment group.

❑ Study Completion

The study completion listing will be developed based on the data obtained in the study completion form.

❑ Patient Disposition

A table representing the counts and percentages of patients attaining the various study milestones namely screen failures, enrolment, randomization, meeting analysis population criteria, protocol deviations, study completion or withdrawal, lost to follow up and un-blinded

by treatment group will be presented. The reason for discontinuation will also be summarized within the table. Section 8 describes the milestones mentioned here. In addition, the number and proportion of patients eligible for and compliant with each follow-up examination will be presented. The proportion seen at any follow-up visit will be computed in two ways: it will be computed based on the eligible patients and it will be computed based on eligible patients minus patients lost to follow-up.

If the proportion of patients withdrawn is larger than the 15% from either treatment group, an analysis of the demographic and prognostic (baseline ISNCSCI Motor Score and severity of the acute spinal cord injury at baseline) characteristics will be made between patients who withdraw and those who remain in the study. For continuous variables, parametric or non-parametric analysis of variance will be used. For categorical variables, Chi-square or Fisher's exact test will be applied.

- ❑ **Prior and Concomitant Medications**

Prior and concomitant medications will be listed and tabulated by treatment group. Listings will present the information collected against these terms and a flag to identify prior and concomitant therapies. Separate tables will be generated to present the counts and percentages of patients with prior and concomitant medications falling in each therapeutic class and chemical subgroup (obtained as part of WHODD coding).

- ❑ **Efficacy and safety endpoints**

All efficacy and safety endpoints will be listed. Tables corresponding to these parameters will be generated as described in the analysis sections below. Graphical representation of the data analyzed will be provided. Vertical bar charts will be provided to represent the change in end points over the visits.

10.4. Analyses of the Patient Populations

The similarity of two treatment groups and similarity of patients from different study sites with respect to important demographic or other variables, either known or suspected to have an influence on the outcome variables, will be determined using two-sided test with significance level 0.05. The absence of similarity for any variable will identify that variable as a potential covariate in subsequent safety and effectiveness analyses.

The demographic and prognostic variables measured at study entry namely baseline ISNCSCI Motor Score and severity of the acute spinal cord injury at baseline will be compared between the treatment groups. Continuous variables will be compared with two-sample t-test or Wilcoxon rank sum test, and categorical variables will be compared with Fisher's exact test or Chi-square test.

All the demographic and prognostic variables will be compared across study sites to determine homogeneity of study sites in patient characteristics. For continuous variables, parametric or non-parametric analysis of variance will be used. For categorical variables, Chi-square or Fisher's exact test will be applied. Factors found to differ significantly by study site will identify that variable as a possible covariate in subsequent analyses. Smaller study sites (those with six or less enrolled patients) with insufficient numbers of patients to allow a meaningful analysis will be combined into one or more pseudo-sites to allow the comparison to be done. The size of any pseudo-site created in this way will not exceed the size of the study site with the largest enrollment.

This analysis will not be used as a basis for data pooling across study sites. Data pooling will be done on a clinical basis, i.e., the sites used a common protocol, the sponsor adequately

monitored the study to assure protocol compliance, and the data gathering and validation mechanisms were the same across all study sites.

In addition, an analysis will be done on primary efficacy endpoint to determine if there is a site by treatment interaction. Site by treatment interactions of a quantitative nature, i.e., all sites show the treatment to be beneficial, but perhaps to a different degree by study site, will not be considered to be an impediment to pooling. Site by treatment interactions that are qualitative in nature, i.e., the vast majority of sites show the treatment to be beneficial, but one or more sites show the treatment to be detrimental, will require extensive evaluation of the sites with contrary results to attempt to determine what factors at those sites led to the result.

In study sites with small numbers of patients, it will not be possible to evaluate a site by treatment interaction. The reason being what may appear to be a site by treatment interaction may be a small numbers phenomenon.

10.5. Subgroup analysis

A sub group analyses will be performed to determine the effect of sites and treatment on the change in ISNCSCI Motor Scores between baseline and follow up visits. Site*treatment interaction will be further assessed. For this purpose, sites with a small number of enrolled subjects will be grouped together. The analyses will be of the type of multiple regressions with ISNCSCI Motor Score as dependent variable, site and treatment as independent variable and age, gender, baseline ISNCSCI Motor Score and severity of the acute spinal cord injury at baseline as confounders.

Further, subgroup analysis will be performed within ASIA Impairment Grades. A pre-planned sub-group analysis will be conducted to evaluate differences in $\Delta ISNCSCIMS_{180\text{-}b}$ among the patients in baseline ASIA Impairment Groups. The rationale for this pre-planned analysis is that these groups experience different recovery of ISNCSCIMS which may result in between-group differences in $\Delta ISNCSCIMS_{180\text{-}b}$. The analyses will be of the type of multiple regressions with change in ISNCSCI Motor Score between baseline and 180 days as dependent variable, baseline ASIA Impairment grades and treatment as independent variable and age, gender, baseline ISNCSCI Motor Score and severity of the acute spinal cord injury at baseline as confounders.

10.6. Primary Analysis

The primary efficacy endpoint in this study is absolute difference in ISNCSCI Motor Score (ISNCSCIMS) between 180 days follow-up and baseline.

$$\Delta ISNCSCIMS_{180\text{-}b} = ISNCSCIMS_{180\ days} - ISNCSCIMS_{baseline}$$

The appropriate statistical approach is to test a single null-hypothesis that the difference between the treatment groups is equal to or less than 0. Rejection of null-hypothesis is consistent with superiority of investigational treatment.

$$H_0: \mu_I - \mu_c \leq 0$$

$$H_1: \mu_I - \mu_c > 0$$

Where:

μ_I and μ_C are the means of the two independent normal distributions;

μ_I is the mean population value ISNCSCI in the Riluzole arm;

μ_c is the mean population value ISNCSCI in the Placebo arm;

The hypothesis will be tested by covariance analysis with the alpha level of 0.025. The actual statistical testing will be performed using SAS PROC MIXED using the treatment group as the CLASS variable. Missing values imputations will be performed using SAS PROC MI as described in above sections and final estimates will be derived using SAS PROC MIANALYZE. Parameters identified as covariates based on analysis between sites will also be taken into consideration. Both imputed and non-imputed samples will be analyzed under the ITT population. Sensitivity analysis will be performed and a descriptive comparison of the results in the two cases will be provided.

The output table will present the descriptive statistics for the ISNCSCI Motor scores at the visits under consideration and the p-values associated with the analysis.

10.6.1. Study success

The study success is defined as follows:

Investigational treatment (riluzole) is superior to placebo. Study statistical success is achieved if the one-sided null hypothesis of no superiority of Riluzole group in $\Delta ISNCSCIMS_{180-b}$ is rejected at alpha level of .025.

10.7. Secondary Analysis

The secondary endpoints will also be analyzed in lines with the approach adopted for the primary endpoint. Thus, the appropriate statistical approach is to test a single null-hypothesis that the difference between the treatment groups is equal to or less than 0. The hypothesis will be tested by two-sided alpha =0.05.

Both imputed and non-imputed samples will be analyzed under the ITT population. Sensitivity analysis will be performed and a descriptive comparison of the results in the two cases will be provided. Parameters identified as covariates based on analysis between sites will also be taken into consideration. The secondary endpoints that will be analyzed are listed below.

- ❑ Change in ISNCSCI sensory score between baseline and 180 days
- ❑ The total Spinal Cord Independence Measure (SCIM) at 180 days

Superiority of Riluzole group over placebo will be tested with respect to the change in ISNCSCI sensory score between baseline and 180 days. A similar analysis will be performed on the reported SCIM values at 180 days.

The output tables will present the descriptive statistics for the scores at the visits under consideration and the p-values associated with the analysis.

10.8. Other Endpoints

The other endpoints mentioned below will also be analyzed in lines with the approach suggested in the above sections.

- ❑ Change in ISNCSCI Sensory Scores from between 180 days and baseline
- ❑ Change in ISNCSCI Upper Extremity Motor Score between 180 days and baseline
- ❑ Change in ISNCSCI Lower Extremity Motor Score between 180 days and baseline
- ❑ Change in SF-36v2.0™ PCS score between 180 days and baseline
- ❑ Change in SF-36v2.0™ MCS score f between 180 days and baseline
- ❑ Change in SF-36v2.0™ eight dimensions between 180 days and baseline
- ❑ Change in EQ-5D health utility from baseline to 84 days, 180 days and 365 days
- ❑ Graded Redefined Assessment of Strength Sensibility and Prehension (GRASSP) at 14 days or Discharge (whichever occurs first) and 180 days.
- ❑ Change in Pain NRS at 14 days, 84 days, and 180 days.

10.9. Safety Analysis and/or further analysis

All the adverse events will be coded using the MedDRA Version 14.1. All the information collected in the AE form in the CRF and the preferred term corresponding to each reported term will be presented as a listing. In addition to this, SAEs will be presented as a separate listing. Further, AEs with start date on or after date of first dose intake will be labeled as TEAEs.

❑ **Other AE tables**

An overview of the adverse events will be presented. All adverse events recorded during the study will be summarized by treatment group. The summary will be comprised of the number of patients with any adverse event, the total number of reported adverse events and unique adverse events.

The incidence of adverse events in each treatment group will be summarized by system organ class, preferred term, severity, relation to the study drug/surgery, outcome and action taken and time-to-event.

Summary tables corresponding to the AEs will contain the incidence of events and the number and percentage of patients experiencing an adverse event. The percentage will be computed against the number of subjects in the mITT population. The 95% exact binomial confidence intervals will also be provided for proportion of patients reporting any particular AE. While counting the number of subjects, a subject with more than one adverse event with the same preferred term will be counted only once using the incidence with highest severity (severity tables), or strongest relationship to study therapy (tables of relationship to treatment) or most severe outcome or action taken. The descending order of the above characteristics will be considered as below

- **Severity**
  - Severe
  - Moderate
  - Mild
- **Relationship to study drug/procedure**
  - Definite
  - Suspected
  - Unrelated
- **Outcome**
  - Death

- Unresolved
- Resolved with Residual Effects
- Resolved
- Unknown

- **Action taken**

  - Remedial therapy (drug/transfusion)
  - Hospitalization
  - Surgical procedure
  - Other conservative approach
  - None

All the above tables will be repeated for TEAEs and serious adverse events. Separate listings will be provided for adverse events and serious adverse events.

A listing of all subjects who discontinued the study due to an adverse event will be provided. The following will be provided for each subject: treatment group, age and gender, type of the event, adverse event description, start and stop dates of the adverse events, severity, and relationship, action taken and whether the adverse event is serious and/or unexpected.

❑ **Clinical Laboratory Data**

The following laboratory samples will be collected during the course of the study.

- Liver Function
- Kidney Function
- Blood Panel

All results corresponding to laboratory evaluations will be converted into SI units. The laboratory results will be listed by patient and visit. Abnormal results will be flagged as H or L based on values being above or below the normal range respectively. Values within the normal ranges will be flagged using **.

The incidences of laboratory abnormalities with respect to the classification of values based on the normal range will be listed by parameter and visit. Summary statistics will be presented for laboratory results with respect to visit along with the change from baseline values. Quantitative results will be summarized using count of patients, mean, standard deviation, minimum, median and maximum. The summary statistics for the change from baseline for quantitative variables will also be presented.

The listing and tables will be presented with respect to the data that is collected at respective visits.

11. Listings, Tables and Figures

For all tables and listings, the titles will appear in the body of the document. The title will include the sponsor name, protocol name, date and time of generation, version (Draft/Final), page number in the format Page X of Y, Table/Listing number and name.

RISCIS

An example is given below

AO Spine North America Draft –DDMMMYYYY:HH:MM

Protocol: RISCIS Page x of y

Table XX.X.X

Table Title

Population

12. Software

SAS Version 9.3 or newer will be used for the analysis
