# Supplementary material for: Safety and Efficacy of Riluzole in Acute Spinal Cord Injury Study (RISCIS): A Multi-Center, Randomized, Placebo-Controlled, Double-Blinded Trial
Source: J Neurotrauma. 2023 Aug 23;40(17-18):1878–88. doi: 10.1089/neu.2023.0163 (PMC10460693; doi:10.1089/neu.2023.0163)
Supplement: Supplemental data [file 5InterimAnalysis.pdf]

A Multi-Center, Randomized, Placebo-Controlled, Double-Blinded, Trial of Efficacy and Safety of Riluzole in Acute Spinal Cord Injury

Interim Statistical Analysis Report

Ver 2020 Oct 22

Prepared by

Nor Consult, LLC

1 Background

Due to the COVID-19 pandemic, screening & enrollment to the RISCIS trial was suspended by the Sponsor (AOSpine North America/AOSNA) on May 1, 2020. At that time, 193 subjects had been enrolled to the study. Given that the trial had almost reached the sample size for the protocol-prescribed pre-planned interim analysis (60%), the Data Safety Monitoring Board recommended to AOSNA to proceed with the interim analysis. The statistical analysis plan for the interim analysis has been created by trial Lead Biostatistician, Dr. Branko Kopjar and approved by the DSMB Statistician Dr. Dale Usner. It is attached as Appendix to this document.

2 Explanation of the Analysis Arms A and B

The analysis is presented as "semi-unblinded". The treatment arms (Investigational and Control) are labeled "A" and "B", in no specific order. In other words, either the Investigational Arm is labeled "A" and Control Arm labeled "B", or Control Arm is labeled "A" and Investigational Arm is labeled "B". The labeling has been created by a data programmer assigned by the Contract Research Organization and only the data programmer knows which label corresponds to what arm. The labeling will be saved in a sealed envelope. Two sets of analyses were performed. First set ("A superior B") assumes that the group A is the Investigational Arm (riluzole). The second set ("B superior A") assumes that the group B is the Investigational Arm (Riluzole). The DSMB will first review the results of the statistical testing without knowledge of the actual arms assignment. The DSMB will make their recommendations for both scenarios (i.e. that the Investigational Arm is labeled A and that the Investigational Arm is labeled B). If the recommendations align (i.e. to continue the trial in either scenario or to stop for futility in either scenario), the DSMB will not be unblinded. However, if the recommendations diverge (e.g. to stop for futility if A is the Investigational Arm but to continue if B Is the Investigational Arm), the DSMB will open the fully unblinded code.

3 Changes Compared to Original Analysis Plan

All changes to original analysis plan were agreed upon between the Lead Biostatistician and DSMB Statistician prior data unblinding and data review.

3.1 Primary Endpoint

The protocol defined primary endpoint for the study is change in International Standards for Neurological Classification of Spinal Cord Injury (ISNCSCI) Total Motor Score between 180-day follow-up and screening/admission (baseline) score.

As a result of observations and developments that were made across the spinal cord injury (SCI) clinical and research communities since the initiation of the trial, there is now an international consensus that ISNCSCI Total Motor Score is not a valid measure for trials of acute SCI.[1] ISNCSCI Total Motor Score is a sum of ISNCSCI Total Motor Score for Lower Extremities and ISNCSCI Total Motor Score for Upper Extremities. The literature consensus shows that the Total Motor Score should not be calculated. Current consensus, and widely recognized practice, is to use change in ISNCSCI Total Motor Score for Upper Extremities between 180-day follow-up and screening/admission (baseline) score as a primary endpoint for trials of treatment of acute SCI.

Prior to any data analysis and prior to unblinding the data, the RISCIS study group decided to switch to ISNCSCI Total Motor Score for Upper Extremities (UEMS). This interim analysis will only be performed using UEMS. Sponsor (AOSNA) and the Principal Investigator were advised of this of this in advance (prior to the review of any study data). The Lead Biostatistician and the DSMB statistician agreed to this change and concurred that no violation of statistical principles occurred and that no alpha value adjustment is required.

3.2 Switching to Non-Binding Futility

The original sequential design is described in the IP and includes one interim analysis. The parameters of the design are provided in the protocol. The independent DSMB statistician has recommended to switch the design to a non-binding futility hypothesis (H1) rejection. The rationale was to allow the DSMB to review a series of sensitivity analyses rather than a single binding approach. This change resulted in an increase in the sample size from 316 to 324. The parameters for the design are provided in Section 0.

[1] Bond LM, McKerracher L. Cervical spinal cord injury: tailoring clinical trial endpoints to reflect meaningful functional improvements. Neural Regen Res. 2014;9(16):1493-1497. doi:10.4103/1673-5374.139470

4 Approach

4.1 Sample Selection for the Interim Analysis

The interim analysis sample includes 184 consecutive subjects enrolled on or before November 2, 2019. The nine remaining enrolled subjects had not yet reached the 180-day follow-up visit at the time when the decision to proceed with interim analysis was made.

4.2 Analysis population

The analysis utilized the intention-to-treat population (ITT).

4.3 Data Imputations

The imputation approach is described in the Statistical Analysis Plan for the Interim Analysis in Appendix). All imputations were performed prior to unblinding the data. Availability of the data are shown in Table 1.

Table 1 Imputation overview Upper Extremity Motor Total Score

	Interim Sample	Baseline	180-day follow-up	Change from baseline#	% change from the baseline available	% of the planned sample size (N=316)
Prior any imputation	183	181	126	124	67.76%	38.27%
After item imputation (Sec 7.2)	183	183	126	126	68.85%	38.89%
After regression imputation (Sec 7.3)	183	183	156	156	85.25%	48.15%
After average imputation (Sec 7.4)	183	183	183	183	100.00%	56.48%

#Change from baseline is Primary Endpoint

5 Alpha and Beta Spending and Threshold p-values

Table 2 Parameters of the sequential design

Plan ID	Parameter
**Type of the Hypothesis**	1-Sided
**Type I Error (α)**	0.025
**Power (1 - β)**	0.90
**Randomization Ratio (Investigational vs. Control)**	1:1
**Planned Number of Interim Looks**	2
**Spacing of Looks**	60%, 100%
**Hypothesis to be Rejected**	H0 or H1 (nonbinding)
**Boundary Family**	Published Function
**Boundary to Reject H0**	Est. O'Brien-Fleming
**Boundary to Reject H1**	Gamma (-1)
**Difference of Means Assuming H_1**	9
**Standard Deviation (σ)**	24.08
**Sample Size**	324 (162 per arm)

Source: Investigational Protocol Table 4.

Table 3 Threshold values for different sizes of interim sample are

	N change from baseline	% change from the baseline available	% of the planned sample size (N=324)	Futility (P > threshold value)	Superiority (P < threshold value)
Prior any imputation	124	67.21%	38.27%	0.45404	0.0002750
After item imputation (Sec 7.2)	126	68.31%	38.89%	0.44387	0.0003079
After regression imputation (Sec 7.3)	156	85.25%	49.37%	0.30251	0.00124
After average imputation (Sec 7.4)	183	100.00%	57.91%	0.20131	0.00323

6 Statistical Test

Statistical testing was performed using mixed model with “itrtrgrp” (A and B) as fixed factor and adjusting for baseline value of the score. All stages of creating an imputed sample for Upper Extremity Motor Score will be included in the analysis. Altogether, this will be four analyses.

7 Results

7.1 Not Imputed Data (CC Population)

Table 4 Upper Total Motor Score LS Mean# by Treatment Arm in Unimputed Data

Treatment arm	N	UMTS LS Mean (95% C.I.)
A	60	13.76 (10.69, 16.83)
B	64	16.32 (13.35, 19.29)

UTMS – Upper Total Motor Score
Adjusted for baseline UMTS

Table 5 Difference in Upper Total Motor Score by Treatment Arm in Unimputed Data

Direction	Difference	Lower C.I.	Upper C.I.	P value (one-sided)	Alpha threshold	Beta threshold	Decision
A super to B	-2.5587	-6.8319	inf.	0.8809	0.0002911	0.44894	Stop: Futility
B super to A	2.5587	-1.7145	inf.	0.1191	0.0002911	0.44894	Continue until next interim

7.2 Item Imputed (CC Population)

Table 6 Upper Total Motor Score LS Mean# by Treatment Arm in Item Imputed Data

Treatment arm	N	UMTS LS Mean (95% C.I.)
A	62	13.55 (10.55, 16.56)
B	64	16.29 (13.34, 19.25)

UTMS – Upper Total Motor Score
Adjusted for baseline UMTS

Table 7 Difference in Upper Total Motor Score by Treatment Arm in Item Imputed Data

Direction	Difference	Lower C.I.	Upper C.I.	P value (one-sided)	Alpha threshold	Beta threshold	Decision
A super to B	-2.7372	-6.949	inf.	0.8996	0.0003253	0.43883	Stop: Futility
B super to A	2.7372	-1.4746	inf.	0.1004	0.0003253	0.43883	Continue until next interim

7.3 Regression Imputed

Table 8 Upper Total Motor Score LS Mean# by Treatment Arm in Regression Imputed Data

Treatment arm	N	UMTS LS Mean (95% C.I.)
A	78	14.10 (11.49, 16.70)
B	78	15.52 (12.92, 18.12)

UTMS – Upper Total Motor Score
Adjusted for baseline UMTS

Table 9 Difference in Upper Total Motor Score by Treatment Arm in Regression Imputed Data

Direction	Difference	Lower C.I.	Upper C.I.	P value (one-sided)	Alpha threshold	Beta threshold	Decision
A super to B	-1.4233	-5.1066	inf.	0.7768	0.00124	0.30251	Stop: Futility
B super to A	1.4233	-2.2599	inf.	0.2232	0.00124	0.30251	Continue until next interim

7.4 Full Imputation (ITT)

Table 10 Upper Total Motor Score LS Mean# by Treatment Arm in Fully Imputed Data (ITT)

Treatment arm	N	UMTS LS Mean (95% C.I.)
A	90	14.03 (11.78, 16.27)
B	93	15.59 (13.38, 17.79)

UTMS – Upper Total Motor Score
Adjusted for baseline UMTS

Table 11 Difference in Upper Total Motor Score by Treatment Arm in Fully Imputed Data (ITT)

Direction	Difference	Lower C.I.	Upper C.I.	P value (one-sided)	Alpha threshold	Beta threshold	Decision
A super to B	-1.5618	-4.7099	inf.	0.8355	0.00286	0.20656	Stop: Futility
B super to A	1.5618	-1.5863	inf.	0.1645	0.00286	0.20656	Continue until next interim

8 Instructions for DSMB Recommendation

DSMB will create a written recommendation for the Sponsor which includes only one of the following possible recommendations:

1. DSMB recommends stopping the trial for efficacy;
2. DSMB recommends stopping the trial for futility;
3. DSMB recommends continuing the trial until the next interim or final analysis.

DSMB Recommendation will be provided

DSMB Statistician will assist DSMB to understand and interpret interim results. Stopping the trial for futility is statistically non-binding. The reason for the non-binding futility option is to allow flexible interpretation of the four statistical sensitivity tests presented in Section 7. Due to the large proportion of missing values for the primary endpoint (N = 59, 33%), and inherent uncertainties concerning the validity of the statistical imputations, none of the four statistical analyses is prevailing. Rather, it is an interpretation of all sensitivity analyses taken together that should inform the DSMB's decision. However, in the case that the statistical testing on any of the four sensitivity analyses indicates futility and the DSMB chooses to recommend the continuation of the trial, the DSMB will document their rationale for doing so. The rationale will not be shared with the Sponsor and will be available to the Sponsor only upon the competition of the trial.

9 Appendix

9.1 DSMB Recommendation Form

A Multi-Center, Randomized, Placebo-Controlled, Double-Blinded, Trial of Efficacy and Safety of Riluzole in Acute Spinal Cord Injury

DSMB Recommendation to Sponsor

After reviewing the results of the Interim Analysis from October 22, 2020 DSMB recommends the following:

_______ DSMB recommends stopping the trial for efficacy.

_______ DSMB recommends stopping the trial for futility.

_______ DSMB recommends continuing the trial until the next interim or final analysis.

Instructions: Place "X" on the line in front of the selected choice and cross out two remaining choices that are not recommended.

____ ______________________

DSMB Chair Date

A Multi-Center, Randomized, Placebo-Controlled, Double-Blinded, Trial of Efficacy and Safety of Riluzole in Acute Spinal Cord Injury

9.2 DSMB Rationale Form

DSMB CONFIDENTIAL RATIONALE

Provide rationale only in the following situations:

- If one of more of the four analyses have indicated that the trial should stop for futility, but the recommendation is to continue the trial.

Do not share the rationale with Sponsor, it will be shared after the final analysis.

Provide Rationale below:

9.3 Statistical Analysis Plan for the Interim Analysis
