# Supplementary material for: Safety and Efficacy of Riluzole in Acute Spinal Cord Injury Study (RISCIS): A Multi-Center, Randomized, Placebo-Controlled, Double-Blinded Trial
Source: J Neurotrauma. 2023 Aug 23;40(17-18):1878–88. doi: 10.1089/neu.2023.0163 (PMC10460693; doi:10.1089/neu.2023.0163)
Supplement: Supplemental data [file 7CONSORTADAbstract.docx]

| **Item** | **Description** | **Reported on line number** |
| --- | --- | --- |
| Title | Identification of study as randomised | 2 |
| Authors | Contact details for the corresponding author | 1 |
| Trial design ^a^ | Description of the trial design (for example, parallel, cluster, non-inferiority); include the word “adaptive” in the content or at least as a keyword | 2 |
| Methods |  |  |
| Participants | Eligibility criteria for participants and the settings where the data were collected | 2 |
| Interventions | Interventions intended for each group | 2 |
| Objective | Specific objective or hypothesis | 2 |
| Outcome ^b^ | Clearly defined primary outcome for this report | 2 |
| Randomisation | How participants were allocated to interventions | 2 |
| Blinding (masking) | Whether or not participants, care givers, and those assessing the outcomes were blinded to group assignment | 2 |
| Results |  |  |
| Numbers randomised | Number of participants randomised to each group | 2 |
| Recruitment | Trial status | 2 |
| Adaptation decisions made ^c^ | Specify what trial adaptation decisions were made in light of the pre-planned decision-making criteria and observed accrued data | n/a |
| Numbers analysed | Number of participants analysed in each group | 2 |
| Outcome | For the primary outcome, a result for each group and the estimated effect size and its precision | 2 |
| Harms | Important adverse events or side effects | n/a |
| Conclusions | General interpretation of the results | 3 |
| Trial registration | Registration number and name of trial register | 3 |
| Funding | Source of funding | 3 |

^a^ Modified item that requires reference to both CONSORT extension for abstracts (Hopewell et al.2008) and ACE;

^b^ Item wording remains unchanged in reference to CONSORT extension for abstracts (Hopewell et al.2008), but we expanded the ACE explanatory text to clarify additional considerations for certain adaptive designs;

^c^ New item that should only be applied in reference to ACE;

All unchanged items require reference to the CONSORT extension for abstracts (Hopewell et al. 2008).

**Citation:**

Dimairo M, Pallmann P, Wason J, Todd S, Jaki T, Julious SA, Mander AP, Weir CJ, Koenig F, Walton MK, Nicholl JP, Coates E, Biggs K, Hamasaki T, Proschan MA, Scott JA, Ando Y, Hind D, Altman DG; ACE Consensus Group. The Adaptive designs CONSORT Extension (ACE) statement: a checklist with explanation and elaboration guideline for reporting randomised trials that use an adaptive design. BMJ. 2020 Jun 17;369:m115. PMID: 32554564; PMCID: PMC7298567.

Dimairo M, Pallmann P, Wason J, Todd S, Jaki T, Julious SA, Mander AP, Weir CJ, Koenig F, Walton MK, Nicholl JP, Coates E, Biggs K, Hamasaki T, Proschan MA, Scott JA, Ando Y, Hind D, Altman DG; ACE Consensus Group. The adaptive designs CONSORT extension (ACE) statement: a checklist with explanation and elaboration guideline for reporting randomised trials that use an adaptive design. Trials. 2020 Jun 17;21(1):528. PMID: 32546273; PMCID: PMC7298968.
