# Supplementary material for: Safety and Efficacy of Riluzole in Acute Spinal Cord Injury Study (RISCIS): A Multi-Center, Randomized, Placebo-Controlled, Double-Blinded Trial
Source: J Neurotrauma. 2023 Aug 23;40(17-18):1878–88. doi: 10.1089/neu.2023.0163 (PMC10460693; doi:10.1089/neu.2023.0163)
Supplement: Supplemental data [file 9CONSORTHarms.pdf]

Table 2. **Checklist of Items To Include When Reporting Harms in Randomized, Controlled Trials***

Standard CONSORT Checklist: Paper Section and Topic	Standard CONSORT Checklist: Item Number	Descriptor	Reported on Page Number
Title and abstract	1	If the study collected data on harms and benefits, the title or abstract should so state.	
Introduction			
Background	2	If the trial addresses both harms and benefits, the introduction should so state.	4
Methods			
Participants	3		
Interventions	4		
Objectives	5		
Outcomes	6	List addressed adverse events with definitions for each (with attention, when relevant, to grading, expected vs. unexpected events, reference to standardized and validated definitions, and description of new definitions).	6
		Clarify how harms-related information was collected (mode of data collection, timing, attribution methods, intensity of ascertainment, and harms-related monitoring and stopping rules, if pertinent).	6
Sample size	7		
Randomization			
Sequence generation	8		
Allocation concealment	9		
Implementation	10		
Blinding (masking)	11		
Statistical methods	12	Describe plans for presenting and analyzing information on harms (including coding, handling of recurrent events, specification of timing issues, handling of continuous measures, and any statistical analyses).	Supplemental
Results			
Participant flow	13	Describe for each arm the participant withdrawals that are due to harms and their experiences with the allocated treatment.	Figure 1
Recruitment	14		
Baseline data	15		
Numbers analyzed	16	Provide the denominators for analyses on harms.	
Outcomes and estimation	17	Present the absolute risk per arm and per adverse event type, grade, and seriousness, and present appropriate metrics for recurrent events, continuous variables, and scale variables, whenever pertinent.†	Table 3
Ancillary analyses	18	Describe any subgroup analyses and exploratory analyses for harms.†	
Adverse events	19		
Discussion			
Interpretation	20	Provide a balanced discussion of benefits and harms with emphasis on study limitations, generalizability, and other sources of information on harms.‡	9-10
Generalizability	21		
Overall evidence	22		

* This proposed extension for harms includes 10 recommendations that correspond to the original CONSORT checklist.
† Descriptors refer to items 17, 18, and 19.
‡ Descriptor refers to items 20, 21, and 22.

Copyright ©2015 American College of Physicians
