# Supplementary material for: Safety and Efficacy of Riluzole in Acute Spinal Cord Injury Study (RISCIS): A Multi-Center, Randomized, Placebo-Controlled, Double-Blinded Trial
Source: J Neurotrauma. 2023 Aug 23;40(17-18):1878–88. doi: 10.1089/neu.2023.0163 (PMC10460693; doi:10.1089/neu.2023.0163)
Supplement: Supplemental data [file 10DSMBCharter.pdf]

A Multi-Center, Randomized, Placebo-Controlled, Double-Blinded, Trial of Efficacy and Safety of Riluzole in Acute Spinal Cord Injury

Data Safety and Monitoring Board (DSMB) Charter

Version 2.2 November 18, 2016

Approval

Data Safety and Monitoring Board (DSMB) Charter

A Multi-Center, Randomized, Placebo-Controlled, Double-Blinded, Trial of Efficacy and Safety of Riluzole in Acute Spinal Cord Injury (SPN-12-001)

Sponsor

AO North America Charitable Foundation d.b.a. AOSpine North America
1700 Russell Road
Paoli, PA 19301

DSMB Chairperson: SIGNATURE ON FILE WITH SPONSOR AS OF NOVEMBER 29, 2016

TABLE OF CONTENTS

1. DATA SAFETY MONITORING BOARD (DSMB) OVERVIEW 6

1.1. Trial Description and Study Design 6

1.2. DSMB Description 6

1.3. DSMB Membership 6

1.4. Reporting 7

2. INTRODUCTION 7

3. STUDY OVERVIEW/SUMMARY 7

3.1. Study Aim 7

3.2. Study Condition and Intervention 7

3.3. Study Design and Phase 7

3.4. Safety Monitoring 8

3.5. Study Endpoints 8

3.6. Follow-up 8

3.7. Safety Evaluation 8

3.8. Regulatory Authority 8

4. ROLES AND RESPONSIBILITIES 9

4.1. DSMB Roles and Responsibilities 9

4.2. Sponsor (or Designees) Roles and Responsibilities 9

5. DSMB MEMBERSHIP 10

5.1. Members and Qualifications 10

5.2. Conflict of Interest 10

6. DSMB MEETINGS 11
6.1. Projected Schedule of Meetings 11
6.2. Scheduling Meetings 12
6.3. Meeting Format 12
6.4. Open and Closed Sessions 12
6.5. Emergency Meeting 13
7. STUDY REVIEW CRITERIA/STOPPING RULES AND GUIDELINES 13
7.1. Safety Analyses 13
7.2. Stopping Guidelines / Stopping Rules: Safety 14
7.3. Effectiveness Analyses 15
7.4. Adaptive Protocol Modification 15
7.5. Consideration of External Data 15
8. DSMB REPORTS 16
8.1. Monitoring for Safety 16
8.2. Monitoring for Effectiveness 16
8.3. Data Flow for Adverse Events 16
8.4. Preparation of Reports to the DSMB 17
8.5. DSMB Communication of Findings and Recommendations 17
8.6. Stopping Procedure 17
8.7. Sponsor’s Response to DSMB Findings and Recommendations 17
9. DSMB CLOSEOUT 18
10. CONFIDENTIALITY 18
11. AMENDMENTS TO THE DSMB CHARTER 19

12. ARCHIVING OF DSMB ACTIVITIES AND RELATED DOCUMENTS 19

13. APPENDICES 20

13.1. Appendix 1: DSMB Membership Roster and Sponsor/CRO contact list 20

13.2. Appendix 2 Conflict of Interest Statement 22

13.3. Appendix 3 Reports to the DSMB- shells of sample tables and listings 23

13.4. Appendix 4 DSMB Recommendation Letter 29

1. Data Safety Monitoring Board (DSMB) Overview

1.1. Trial Description and Study Design

i Trial name: A Multi-Center, Randomized, Placebo Controlled, Double-Blinded, Trial of Efficacy and Safety of Riluzole in Acute Spinal Cord Injury

ii Trial Sponsor: AOSpine North America Charitable Foundation (hereinafter referred to as "Sponsor")

iii Trial funding agencies and other Sponsors: AOSpine International, Rick Hansen Institute, Christopher and Dana Reeve Foundation, Ontario Neurotrauma Foundation, and the United States Department of Defense.

iv Trial management: Nor Consult LLC (hereinafter referred to as "CRO")

v Trial design: International, multi-center, prospective, double-blinded, placebo-controlled randomized in 1:1 ratio between the investigational and control arms, and adaptive statistical design.

vi Phase: Phase II/III

vii Number of patients: 351

viii Number of sites: Up to 40

1.2. DSMB Description

i This DSMB will be coordinated by the CRO.

ii This DSMB will be independent of the Sponsor and any other Trial funding agencies, CRO, regulatory agencies, Institutional Review Boards (IRBs)/Research Ethics Boards (REBs)/Ethics Committees (ECs), and Investigators.

iii This charter will be approved by the DSMB Members as attested to by signature of the Chairperson.

1.3. DSMB Membership

i Members will disclose conflicts of interest and will be cleared of significant conflicts of interest and potential conflicts of interest in accordance with the provisions of this charter.

ii DSMB Members will sign confidentiality agreements covering their DSMB activities.

iii The DSMB will be comprised of five (5) voting Members ("Members").

iv The DSMB will be comprised of three (3) physicians who collectively are specialized in Spinal Trauma, Internal Medicine and Rehabilitation Medicine. The DSMB will also include one toxicologist and one biostatistician. Due to the complexity and novelty of the adaptive design, the biostatistician selected for the DSMB must have a proper understanding of the method for sample size re-adjustment. All Members must have sufficient computer skills to effectively use MS Office Word and Excel

v Remuneration will be provided by the Sponsor.

1.4. Reporting

i Data reviewed by the DSMB will be provided by a statistician/SAS Programmer from the CRO.

ii Issues and recommendations identified by the DSMB will be provided to the RISCIS Steering Committee and the CRO by the DSMB Chairperson in accordance with this charter.

iii Details of closed session deliberations (e.g., minutes) will be considered privileged and not subject to disclosure except as required by law.

2. Introduction

2.1. The purpose of this charter is to define the roles and responsibilities of the Data Safety Monitoring Board (DSMB), delineate Membership qualifications, describe the purpose and timing of meetings, provide the procedures for ensuring confidentiality and proper communication, and outline the content of the reports.

2.2. The DSMB will function in accordance with the principles of the following documents: ICH GCP E6 (*http://www.ich.org/products/guidelines/efficacy/efficacy-single/article/good-clinical-practice.html*) and the FDA document "Guidance for Clinical Trial Sponsors: On the Establishment and Operation of Clinical Trial Data Monitoring Committees" (*http://www.fda.gov/RegulatoryInformation/Guidances/ucm127069.htm*)

3. Study Overview/Summary

3.1. *Study Aim*

i The aim of this study is to evaluate the efficacy and safety of riluzole in the treatment of patients with acute Spinal Cord Injury (SCI). The primary objective is to evaluate the superiority of riluzole, at a dose of 100 mg BID (Twice Daily) the first 24 hours followed by 50 mg BID for the following 13 days after injury, as compared to placebo, measured by change between 180 days post-study enrollment and baseline in motor outcomes (measured using International Standards for Neurological Classification of Spinal Cord Injury Examination (ISNCSCI) Motor Score), in patients with acute traumatic SCI, presenting to the hospital less than 12 hours after injury. Secondary objectives are to evaluate the effects of riluzole on overall neurologic recovery, sensory recovery, functional outcomes, quality of life outcomes, health care utilization, mortality, and adverse events. The working hypothesis is that the riluzole treated subjects will experience superior motor, sensory, functional, and quality of life outcomes as compared to those receiving placebo, with an acceptable safety profile.

3.2. *Study Condition and Intervention*

i Condition: Acute (≤ 12 hours duration) traumatic SCI

ii Drug:

- Experimental: Riluzole 100 mg BID first 24 hours followed by 50 mg BID for 13 days
- Control: Placebo

3.3. *Study Design and Phase*

i Allocation: Randomized, stratified by site

ii Endpoint classification: Efficacy/Safety
iii Model: Parallel assignment
iv Masking: Double-blinded
v Primary purpose: Treatment
vi Phase: II/III
vii Sample Size: 351
viii Statistical Design: Adaptive design

3.4. *Safety Monitoring*

i Safety will be monitored throughout the course of the study by a designated Medical Safety Officer. Trends in serious adverse events (SAE), laboratory events, and treatment-emergent adverse events (TEAE) will be reviewed by an external Data Safety and Monitoring Board (DSMB). The DSMB will evaluate safety information against the pre-specified safety stopping rules.

3.5. *Study Endpoints*

i PRIMARY EFFICACY ENDPOINT

- *Change in ISNCSCI Total Motor Score between 180 days and Baseline*

ii SECONDARY EFFICACY ENDPOINTS

- *Change in American Spinal Injury Association Impairment Scale (AIS) grade between 180 days and Baseline*
- *Spinal Cord Independence Measure (SCIM) at 180 days*

iii OTHER ENDPOINTS

- *Change in ISNCSCI Sensory Scores (Light Touch and Pin Prick) between 180 days and baseline*
- *Change in ISNCSCI Upper Extremity Motor Score between 180 days and baseline*
- *Change in ISNCSCI Lower Extremity Motor Score between 180 days and baseline*
- *Change in Short Form 36 Version 2 (SF-36v2™) Physical Component Summary (PCS), Mental Component Summary (MCS) and 8 dimensions between 180 days and pre-injury (recall)*
- *Change in EQ-5D health utility between 180 days and pre-injury (recall)*
- *Graded Redefined Assessment of Strength Sensibility and Prehension (GRASSP) at 14 days or Acute Discharge (whichever occurs first) and 180 days*
- *Change in Numeric Pain Rating Scale (Pain NRS) at 14 days, 84 days and 180 days*

3.6. *Follow-up*

i Surgery (if applicable); 72 ± 12 hours post-injury; 7 ± 1 day; 14 + 2 days after enrollment; Discharge from acute care; 84 ±14 days; 180 ± 30 days; 365 ± 45 days.

3.7. *Safety Evaluation*

i Comparison of the rate of adverse events by body system between the riluzole and placebo arms.

3.8. *Regulatory Authority*

i USA: Institutional Review Boards (IRB) at participating sites. This study is exempted from the requirements for an Investigational New Drug (IND) application per Federal Drug Application (FDA) regulations [21 CFR 312.2(b)]

ii Canada: Health Canada and Research Ethics Boards (REBs)
iii Australia: Therapeutic Goods Administration (TGA) and Human Research Ethics Committees (HREC)
iv European Countries: Competent authorities and Ethics Committees in respective countries

4. Roles and Responsibilities

4.1. *DSMB Roles and Responsibilities*

i Meet periodically (see DSMB Meetings) to review aggregate and individual subject data related to the safety of the investigational treatment
ii Review the results of the Interim Statistical Analysis for efficacy, which may lead to early trial termination for success, adaptive changes in the sample size or discontinuation of the study for futility (see Study Review Criteria/Stopping Rules and Guidelines).
iii The DSMB will review the Safety Officer's reports regarding severe adverse events (SAEs) and unexpected adverse events (UAEs). The DSMB will pay particular attention to reports of abnormal liver function tests and liver toxicity.
iv The DSMB will advise the RISCIS Steering Committee and the CRO regarding the continuing safety of trial subjects, including future subjects.
v Provide recommendations to the RISCIS Steering Committee /CRO in regards to continuing or terminating the trial depending upon ongoing review and the Interim Statistical Analysis. The DSMB will recommend additional safety measures, including potentially recommending that the study be terminated early, if needed.
vi Communicate other recommendations or concerns as appropriate.
vii Operate according to the procedures described in this charter and all other applicable guidance documents.
viii Follow conflict of interest guidelines as detailed below (see DSMB Membership).
ix Comply with confidentiality procedures as described below (see Confidentiality).
x Maintain documentation and records of all activities as described below (see DSMB Meetings, DSMB Reports*).*

4.2. *Sponsor (or Designees) Roles and Responsibilities*

i The Sponsor will directly provide funding for the study and DSMB.
ii The Sponsor has delegated the following responsibilities to the CRO:

- Assure the proper conduct of the study.
- Assure collection of accurate and timely data (monitoring and data management).
- Compile and report SAEs to the Independent Medical Safety Monitor (IMSM) for SAE Assessment
- Report SAE Assessment from Independent Medical Safety Monitor (IMSM) to DSMB.
- Promptly report potential safety concern(s) to the DSMB.
- Prepare summary reports of relevant data for the DSMB. (This may include analyses not otherwise outlined in this charter based upon findings.)
- Provide an independent facilitator for presentation of results during DSMB meetings if requested by the DSMB.

- Communicate with regulatory authorities, IRB/EC, and Investigators, in a manner that maintains integrity (e.g., blinding) of the data, as necessary. (This communication is not the responsibility of the DSMB.)
- CRO will provide clinical data to the DSMB Members and request additional clinical information from a study site as requested by the DSMB Members. The CRO employees are prohibited from providing any opinions to the DSMB Members which may interfere with the independent judgement of the DSMB Members.

5. DSMB Membership

5.1. *Members and Qualifications*

The DSMB will consist of five (5) Members; of which three have had substantive previous DSMB experience as listed in Appendix 1. The Chairperson has previously served on a DSMB for one study and has three years of cumulative years of service on DSMBs. The DSMB Members have been selected by the Sponsor in consultation with the trial CRO and Investigators.

As characteristic qualifications, Members will:

i Work professionally and meet qualifications for their respective professions as listed on Membership composition in 5.1.

ii Comply with accepted practices of their respective professions.

iii Comply with conflict of interest policies specified by Sponsor in section 5.4 to ensure that Members do not have serious scientific, financial, personal, or other conflicts of interest related to the conduct, outcome, or impact of the study according to the guidelines specified below.

iv Be independent from the Sponsor, IRB/EC, regulatory agencies, principal Investigator, co-principal or sub-principal Investigator, site Investigator, site Sub-Investigator, RISCIS Steering Committee Members, RISCIS advisory board Members, AO North America Committee Membership, clinical care of the study subjects, or any other capacity related to trial operations, as applicable.

v Not be on the list of Notice of Initiation of Disqualification Proceedings and Opportunity to Explain (NIDPOE) (http://www.fda.gov/foi/nidpoe/default.html) and/or debarred list of Investigators.(http://www.fda.gov/ICECI/EnforcementActions/FDADebarmentList/default.htm)

vi Agree to provide proper documentation in the unlikely event that s/he is unable to continue participating on the DSMB. Although each DSMB member will be expected to serve for the duration of the trial, in the event that a member is unable to continue participation, the reason will be documented and a replacement will be selected by the Sponsor.

5.2. *Conflict of Interest*

i DSMB Membership has been restricted to individuals free of apparent major conflicts of interest. The source of these conflicts may be financial, scientific, or regulatory in nature. Thus, neither study Investigators nor individuals employed by the Sponsor or CRO, nor individuals who might have regulatory responsibilities for the trial product, are eligible to be Members of the DSMB. Any questions or concerns that arise regarding conflicts of

interest will be addressed by the DSMB Chairperson with input from other DSMB Members and Sponsor or CRO as necessary.

ii The DSMB will follow conflict of interest guidelines referenced by the Department of Health and Human Services: Financial Relationships and Interests in Research Involving Human Subjects: Guidance for Human Subject Protection (http://www.hhs.gov/ohrp/archive/humansubjects/finreltn/fguid.pdf). DSMB Members will sign a non-conflict of interest statement in regard to this study which will be on file with Nor Consult LLC. See a Conflict of Interest Statement template in Appendix 2.

iii DSMB Members will be responsible for advising the DSMB Chairperson and Sponsor of any changes in their financial interests in pharmaceutical companies, biotechnology companies, medical device companies, and CROs, including consulting for such companies that may represent a potential conflict of interest for this trial. The member must make such disclosure within two months. In such cases, the DSMB meeting minutes must document the disclosure of the potential conflict of interest and the outcome of the discussion, e.g., abstention of member from voting. The DSMB Chairperson, upon consultation with the Sponsor, will be responsible for deciding whether consultancies or the financial interests of the Members materially affect their objectivity.

iv DSMB Members who develop potential or significant perceived conflicts of interest will be asked to resign from the DSMB. If time permits, a replacement member will be selected prior to the next meeting.

6. DSMB Meetings

6.1. *Projected Schedule of Meetings*

i An initial meeting of the DSMB will be held prior to any subject enrollment in the study in order for the Members to review the charter, to form an understanding of the protocol and definitions being used, to establish a meeting schedule, and to review the study modification and/or termination guidelines. Subsequent interim and final review meetings will be held to review and assess safety data throughout the course of the clinical trial. The DSMB will convene as often as necessary, at a minimum twice a year during the enrollment phase as described in the table 1 below:

Table 1 Projected Meeting Schedules

Timeline	Data Review by	Type of Data
The first data review meeting will occur within the first six months of enrollment or no later than the first 100 subjects enrolled	Entire DSMB	— Adverse events listing coded by treatment arm
Every 6 months after the first data review meeting	Entire DSMB	— Adverse events listing coded by treatment

		arm<br>— Protocol deviations listing<br>— Liver function data listing with subject has any AST and ALP ≥ 3 times ULN coded by treatment arm<br>— Enrollment summary
During the Interim Analysis phase	Entire DSMB	Same as above,<br>Overall primary and secondary endpoints
Upon completion or termination of study	Entire DSMB	Same as above, coded by treatment arm

6.2. *Scheduling Meetings*

i CRO will assist with meeting scheduling and coordination.

ii Meeting notifications with information to be discussed will be provided to Members, by the CRO, in writing at least 30 calendar days in advance of the meeting. This will provide Members the opportunity to request additional support information, such as clinical histories and surgical reports. Such information will be provided to the Members prior to the DSMB meeting. The DSMB may request additional information to be provided after the meeting, if necessary.

iii Members will exercise their best efforts to attend all meetings and to review information regarding the issues to be discussed in advance in order to ensure that they are adequately prepared to make informed judgments.

iv In the event that a Member is unable to attend a DSMB meeting, such Member agrees to provide a written response to any questions issued for discussion at the meeting.

6.3. *Meeting Format*

i DSMB data review meetings will generally be conducted via teleconference or through a web meeting coordinated by the CRO. A quorum, defined as having at least 3 Members including the Chairperson and the statistician present, will be required to hold a DSMB meeting. Critical decisions of the DSMB should be made by unanimous vote. However, if this is not possible, majority vote will decide.

ii A Facilitator from the CRO (the person responsible for preparing the data that will be reviewed by the DSMB or a person familiar with the data) will attend the DSMB meetings as a non-voting member in order to facilitate data presentation and follow-up reporting, unless deemed not necessary by the DSMB. The meetings will include both open and closed sessions.

6.4. *Open and Closed Sessions*

i The open session is optional. The Chairperson may choose to have an open session prior to the closed session. The open session may be attended by representatives of the Sponsor, Investigator and employees of the CRO. Data presented in the open session may include

enrollment data, individual adverse event data, baseline characteristics, and other logistical data. Minutes of the open session will be recorded by the Recorder, an employee of the CRO, who will assume responsibility from the Clinical Research Executive (CRE) of the CRO. Minutes will be finalized upon signature of the Chairperson and maintained by the CRO in accordance with the CRO's internal filing guidelines.

ii The closed session will be restricted to the DSMB Members, a facilitator, and a recorder. Data which may compromise the integrity of the study (e.g., comparative data) will be analyzed and discussed only in the closed session. The minutes of the closed session will be recorded by the Recorder. Minutes from the closed session will be recorded separately from the minutes of the open session and stored securely by the CRO. Closed session minutes, finalized by signature of the Chairperson, will be maintained in confidence and retained until discarded in accordance with applicable study document retention regulations.

iii Following each meeting, a report separate from the minutes of the open and closed sessions will be sent to the Sponsor describing the DSMB recommendations and their rationale (see DSMB Communication of Findings and Recommendations).

6.5. *Emergency Meeting*

i The Sponsor/CRO, the RISCIS Steering Committee or the Independent Medical Safety Monitor may request an emergency meeting of the DSMB to address critical medical issues that may arise during the course of the study. The DSMB will schedule a meeting as soon as possible, at most within ten (10) business days of receipt of such a request. The requirement of written advance notice to Members will be waived in the event of a request by the Sponsor/CRO for an emergency meeting.

7. Study Review Criteria/Stopping Rules and Guidelines

Guidance for the conduct of safety analyses, stopping rules and adaptive protocol modification, if applicable, will be established prior to the DSMB's first evaluation of the data.

7.1. *Safety Analyses*

i The safety analyses will be performed using the modified intention to treat (mITT) Safety Population. Through regularly scheduled meetings and ad-hoc meetings or teleconferences, the DSMB will provide ongoing review of cumulative safety data to determine whether*:*

- The subject entry and treatment are appropriate.
- The protocol meets appropriate needs for safety monitoring and conduct.
- Continued study conduct is justified.

ii The DSMB will review safety data inclusive of the safety experience of each subject including data listing of the liver function data of subjects who have at least one ALT or AST test result ≥ 3 times the upper limit of normal (ULN) and cumulative occurrences of AEs, SAEs, and reasons thereof.

iii The DSMB will perform safety assessments, identify Unexpected Adverse Events from SAEs and make a decision on study progress which may include:

- Continuing the study without modification
- Modifying the study
- Suspending or terminating enrollment in the study at any time
- Permitting re-commencement of enrollment following a suspension

iv AE Definitions
The DSMB will use the following definitions, as specified in Section 9.1 of the Investigational Protocol:

- Adverse Event (AE): An *Adverse Event* is any untoward medical occurrence associated with the use of a drug in humans, whether or not it is considered drug related. AEs may occur at any time during study participation and are not limited to the period of exposure to the Investigational Drug. AEs may include abnormal laboratory findings, medical complications and changes in the subject's condition.
- A Suspected Adverse Reaction: is any Adverse Event for which there is a reasonable possibility that the drug caused the adverse event. 'Reasonable possibility' means that there is evidence to suggest a causal relationship between the drug and the adverse event. A suspected adverse reaction implies a lesser degree of certainty about causality than an adverse reaction.
- Unexpected Adverse Event/Reaction: An Adverse Event or Suspected Adverse Reaction is considered "unexpected" if it is not listed in the Investigator brochure or is not listed at the specificity or severity that has been observed; or, if an Investigator brochure is not required or available, is not consistent with the risk information described in the general investigational plan. When new Adverse Event information is received, the Safety Officer and DSMB will be responsible to determine whether the event is "unexpected" for safety reporting purposes

- Serious (or Life-threatening) Adverse Events or Adverse Reaction:
  An Adverse Event or suspected Adverse Reaction is considered "serious" if, in the view of either the Investigator or the Sponsor, it results in any of the following outcomes:
  - Leads to death
  - Is life threatening, or places the participant at immediate risk of death
  - Requires or prolongs hospitalization
  - Results in a significant, persistent or permanent change, impairment, damage or disruption in the patient's body function/structure, physical activities and/or quality of life
  - Results in a congenital anomaly/birth defect
  - Any other serious or important event that may jeopardize the patient and may require medical or surgical intervention (treatment) to prevent one of the other outcomes

7.2. *Stopping Guidelines / Stopping Rules: Safety*

i The study may be discontinued if the Sponsor decides it necessary for medical, safety, regulatory or other reasons consistent with applicable laws, regulations and GCP. The

study may also be discontinued if the interim analysis demonstrates clear efficacy or futility.

ii The DSMB may recommend termination or modification of the study if any of the following predefined conditions are met:

- Withdrawal of riluzole from the US or Canadian markets, by the FDA or Health Canada. Withdrawal from other markets will be reviewed and a recommendation about the continuation of the study will be provided to the Sponsor.
- Occurrence of one unexpected Adverse Event (UAE) leading to death that is definitely or probably due to the Investigational Medication (riluzole).
- Statistically significant increase in neurological SAEs in riluzole compared to placebo group at the time of the interim analysis.
- Statistically significant increase in SAEs that are definitely or probably related to riluzole at the time of the interim analysis.

iii In addition, termination or modification may be recommended for any other perceived safety concern based on clinical judgment, including but not limited to a higher than anticipated rate for any component of the primary endpoint, or Unanticipated Adverse Events.

iv To ensure timely communication of the recommendation to stop the study if any of the stopping criteria are satisfied, the DSMB will follow the stopping procedure described in section 8.7 of this document.

7.3. *Effectiveness Analyses*

The primary effectiveness endpoint is absolute difference in ISNCSCI Motor Score (ISNCSCIMS) between 180-day follow-up and baseline. As defined in the study protocol, the DSMB will review the interim analysis when 60% of the total anticipated data is available from enrollment through the 180 day follow-up point.

7.4. *Adaptive Protocol Modification*

- The adaptive adjustments will be performed after the first interim analysis of the data, currently planned at 60% data available at 180 days. The details of the adaptive changes are described in a separate Statistical Analysis Plan. The DSMB will review the primary safety and effectiveness hypotheses in order to assess the need for a modification to the trial design. The anticipated modification is a sample size re-estimation.

7.5. *Consideration of External Data*

- The DSMB will also consider data from other studies or external sources during its deliberations, if available, as these results may have a profound impact on the safety of the patients and design of the current study.

8. DSMB Reports

8.1. *Monitoring for Safety*

- The primary duty of the DSMB is to monitor the study for patient safety. Formal DSMB safety reviews will occur as specified in this document. The following is a list of anticipated data tables that will be provided to the DSMB Members by an unblinded support statistician or SAS programmer assigned by the CRO who will be independent from the Sponsor:

Item	Type	Topic
1	Table	Adverse events by body system
2	Table	Adverse events by body system and time of occurrence
3	Table	Drug-related adverse events by body system
4	Table	Serious adverse events by body system
5	Table	Serious adverse events by body system and time of occurrence
6	Table	Drug-related serious adverse events by body system
7	Table	Liver toxicity: ALT or AST ≥3 times upper limit of normal
8	Listing	Lab values for subjects with ALT or AST ≥3 times upper limit of normal
9	Table	Deaths
10	Listing	AEs among deaths
11	Table	Hypotensive AEs
12	Listing	Hypotensive AEs
13	Table	Demographics
14	Table	AIS grade at baseline
15	Table	ISNSCI motor score total at baseline
16	Table	ISNSCI neurological level at baseline

8.2. *Monitoring for Effectiveness*

- The DSMB will review effectiveness outcomes during the Interim Statistical Analysis to determine relative risk/benefit, futility, or for early termination due to overwhelming effectiveness.

8.3. *Data Flow for Adverse Events*

- The DSMB will periodically and carefully monitor adverse events throughout the duration of the study. This process will be dynamic to include semi-annual reviews of all reported SAEs by the entire DSMB. The Investigators will be expected to report Serious Adverse Events (SAEs) to the CRO within 24 hours of knowledge of the event. The CRO will then report it to the Medical Safety Officer. The DSMB will monitor the accumulated SAE report from all of the sites and the SAE assessment from the Medical Safety Officer to identify trends in SAEs.

8.4. *Preparation of Reports to the DSMB*

i Formal DSMB reviews will include data in; this may include table shells or tables and figures. Appendix 3 provides a sample data table format. The statistician or the SAS programmer from CRO will prepare and distribute reports to the DSMB. The reports will be delivered electronically approximately 30 days prior to the date of each DSMB meeting.

ii In order to provide the maximum amount of information to the DSMB, the analyses will employ the most recent data available at the time of the analysis. Requests for additional data by the DSMB Members will be made to the DSMB Chairperson or his or her designee, who will be responsible for communicating the request to the statistician or the SAS programmer from the CRO. The CRO will send a reminder to all Members seven (7) days prior to each meeting in order to obtain any additional requests for information.

8.5. *DSMB Communication of Findings and Recommendations*

i Following each meeting and within five (5) working days of the meeting, the Chairperson will send the findings and recommendations of the DSMB via a DSMB Recommendation Letter (Appendix 4) to the DSMB Coordinator from the CRO. The DSMB Coordinator will distribute the recommendation to the Sponsor and the RISCIS Steering Committee.

ii These findings and recommendations can result from both the open and closed sessions of the DSMB meeting. If these findings include serious and potentially consequential recommendations that require immediate action, the Chairperson will also promptly notify the Study Principal Investigator Dr. Michael Fehlings and the Study Director Dr. Branko Kopjar by phone.

8.6. *Stopping Procedure*

i To ensure the timely communication of the recommendation to stop the study if any of the stopping criteria are satisfied, the DSMB will be required to report its recommendation to the Sponsor/CRO via a preliminary written report within 72 hours. A complete written report must follow within five (5) business days.

ii Upon receiving the DSMB's report, the Sponsor may make a final determination to stop the study. If the conclusion was reached to stop the study, Sponsor/CRO will notify all participating Investigators and the ethical boards. FDA, Health Canada and other regulatory agencies will be advised as needed / applicable within 72 hours. Upon its completion, a copy of the final DSMB report will also be provided to the regulatory agencies.

8.7. *Sponsor's Response to DSMB Findings and Recommendations*

i The RISCIS Steering Committee will review and respond to the DSMB recommendations. The recommendations of the DSMB will not be legally binding but they will require professional consideration by the recipients. If the DSMB recommends continuation of the study without modification, no formal response will be required. However, if the recommendations request action, such as a recommendation of termination of the study or modification of the protocol, the DSMB will request that the Sponsor provide a formal

written response stating whether the recommendations will be followed and the plan for addressing the issues.

ii It is recognized that the Sponsor may need to consult with regulatory agencies or other consultants before finalizing the response to the DSMB. Upon receipt, the Sponsor will consider the DSMB's response and will attempt to resolve relevant issues, resulting in a final decision. Appropriate caution will be taken by the Sponsor during this process to avoid compromising the integrity of the study or the ability of the Sponsor to manage the study, should the study continue. The Sponsor will agree to disseminate the final decision to the appropriate regulatory agencies, ethical boards, and Investigators within an appropriate time.

iii In the unlikely event of irreconcilable differences, especially regarding study termination or other substantial study modifications, the DSMB may decide to discontinue monitoring the current study and disband. This decision will be communicated to the RISCIS Steering Committee.

iv Public disclosure of the Sponsor's final decision or DSMB recommendations will be at the discretion of the Sponsor or their designee. The DSMB will not make any public announcements either as a group or individually.

9. DSMB Closeout

This study may be terminated under a variety of circumstances including, but not limited to, termination for overwhelming effectiveness, futility, or safety issues per protocol or DSMB monitoring guidelines. The responsibilities of the DSMB with regard to closeout will be to review the final study report to ensure study integrity. The DSMB may recommend continuing action items to the CRO based upon the final review.

10. Confidentiality

All data provided to the DSMB and all deliberations of the DSMB will be privileged and confidential. The DSMB will agree to use this information to accomplish the responsibilities of the DSMB only and will not use it for other purposes without written consent from the Sponsor, RISCIS Steering Committee and the CRO. No communication of the deliberations or recommendations of the DSMB, either written or oral, will occur except as required for the DSMB to fulfill its responsibilities. Individual DSMB Members must not have direct communication regarding the study outside the DSMB including, but not limited to the Investigators, ethical boards, regulatory agencies, or Sponsor, except as authorized by this DSMB charter.

11. Amendments to the DSMB Charter

This DSMB charter can be amended as needed during the course of the study. Information to be included as amendments are: any modifications or supplements to the reports prepared for the DSMB, as well as amendments to other information addressed in this charter. All amendments will be documented with sequential version numbers and revision dates, and will be recorded in the minutes of the DSMB meetings. Each revision will be reviewed and agreed upon by both the RISCIS Steering Committee, CRO and the DSMB. All versions of the charter will be archived in accordance with this document (see Archiving of DSMB Activities and Related Documents).

12. Archiving of DSMB Activities and Related Documents

All DSMB documentation and records will be retained by the CRO at its business address: 677 Strander Blvd. Suite F, Seattle, WA 98188, USA for at least two years after completion of the study. Access to the archived data will be controlled by the CRO which will release the information only as specified in this charter or as required by law.

13. Appendices

13.1. *Appendix 1: DSMB Membership Roster and Sponsor/CRO contact list*

Name	Contact Info	Specialty	# of years previous DSBM experience	# of Study(ies) previous DSBM experience
**Chairperson:**	Dr. Anthony Burns<br>Anthony.Burns@uhn.ca	Physiatrist, Spinal Cord Injury Rehabilitation	3	1
**Member:**	Dr. Dale Usner<br>21 East 6th Street, Suite 110<br>Statistics Data Corporation<br>Tempe, AZ 85281<br>dusner@sdcclinical.com	Biostatistician	**14**	**19**
**Member:**	Dr. Simon Carette<br>Simon.carette@uhn.ca	Internist, Rheumatologist	0	0
**Member:**	Dr. Thomas Bryce<br>thomas.bryce@mountsinai.org	Physiatrist, Spinal Cord Injury Medicine	3	2
**Member:**	Dr. Carlo Santaguida<br>Carlo.santaguida@mcgill.ca	Spine Neurosurgeon	0	0

Sponsor / CRO Contact List

Study Function	***Contact Info***
Study PI	Michael G. Fehlings MD PhD FRCSC FACS<br>Toronto Western Hospital<br>University Health Network<br>4 West Wing, Room 423<br>399 Bathurst St.<br>Toronto, Ontario Canada M5T2S8<br>Phone: + 1 416. 603. 5627<br>Fax: +1 416. 603. 5298<br>Email: Michael.Fehlings@uhn.ca
Study Director	Branko Kopjar, MD, MS, PhD<br>Nor Consult, LLC<br>677 Strander Blvd. Bldg. F<br>Seattle, WA 98188<br>Phone: + 1 206.607.6861<br>Fax: + 1 206.575.2002<br>Email: branko.kopjar@nor-consult.com
DSMB Coordinator	Veljko Kopjar, M.A.<br>Nor Consult, LLC<br>677 Strander Blvd. Suite F<br>Seattle, WA 98188<br>Ph: +1 206. 607. 6861<br>Fax: +1 206. 575. 2002<br>Email: veljko.kopjar@nor-consult.com
Statistician or SAS programmer	Kevin Beverly<br>677 Strander Blvd. Suite F<br>Seattle, WA 98188<br>Ph: +1 206. 607. 6861<br>Fax: +1 206. 575. 2002<br>Email: kevin.beverly@nor-consult.com
Lead Monitor	Wynne Xie, MD, MS<br>Nor Consult, LLC<br>677 Strander Blvd. Bldg. F<br>Seattle, WA 98188<br>Phone: + 1 206.607.6861<br>Fax: + 1 206.575.2002<br>Email: wynne.xie@nor-consult.com
Study Assistant	Tiffany Ogami<br>Nor Consult, LLC<br>677 Strander Blvd. Bldg. F<br>Seattle, WA 98188<br>Phone: + 1 206.607.6861<br>Fax: + 1 206.575.2002<br>Email: riscis@nor-consult.com

13.2. *Appendix 2 Conflict of Interest Statement*

I am making a declaration that I will disclose any equity or other financial compensation that exceeds the limits outlined for United States clinical investigators in the Financial Disclosure regulations published in 21 CFR Part 54.4 (check one):

☐ I have no interest(s) to declare
☐ I have interest(s) to declare (see below)

Please identify the interest (e.g., any proprietary or financial interest, including any claims under patent or other intellectual property rights; rights to royalties or other compensation due upon sale; equity interest in organizations with rights to the research product; or consulting fees or other compensation for services rendered with respect to the research product):

____

____

____

____

____

____

____

____

____

____

In addition to the preceding, I further declare that I am not an institutional Investigator participating in this study and that I have read and am thoroughly familiar with the objectives of this study.

________________________________ ______________________

DSMB Member Date

13.3. *Appendix 3 Reports to the DSMB- shells of sample tables and listings*

Item 1: Adverse events by body system

	Rizulole(N=.)		Control(N=.)		Total(N=.)	
**System Organ Class[1]**	**Subjects[2]**	**Events**	**Subjects[2]**	**Events**	**Subjects[3]**	**Events**
Any adverse event	. (%)	.	. (%)	.	. (%)	.
Infections and infestations	. (%)	.	. (%)	.	. (%)	.
Respiratory, thoracic and mediastinal disorders	. (%)	.	. (%)	.	. (%)	.
Gastrointestinal disorders	. (%)	.	. (%)	.	. (%)	.
General disorders and administration site conditions	. (%)	.	. (%)	.	. (%)	.
Vascular disorders	. (%)	.	. (%)	.	. (%)	.
Nervous system disorders	. (%)	.	. (%)	.	. (%)	.

…

1 Adverse events are coded using MedDRA version 17.0.
2 Each subject is counted only once in the respective Preferred Term or System Organ Class.

Item 2: Adverse events by body system and time of occurrence

System Organ Class[1]	Treatment	Pre-Randomization	0-14[2]	15-30	>31
Any adverse event	Riluzole	.	.	.	.
	Placebo	.	.	.	.
Infections and infestations	Riluzole	.	.	.	.
	Placebo	.	.	.	.

**System Organ Class[1]**	**Treatment**	**Pre-Randomization**	**0-14[2]**	**15-30**	**>31**
Respiratory, thoracic and mediastinal disorders	Riluzole	.	.	.	.
	Placebo	.	.	.	.
Gastrointestinal disorders	Riluzole	.	.	.	.
	Placebo	.	.	.	.
Nervous system disorders	Riluzole	.	.	.	.
	Placebo	.	.	.	.

…

1 Adverse events are coded using MedDRA version 17.0.
2 Day 0 is day of randomization.

Item 3: Drug-related adverse events by body system

[Same as Item 1 but restricted to drug-related adverse events]

Item 4: Serious adverse events by body system

[Same as Item 1 but restricted to serious adverse events]

Item 5: Serious adverse events by body system and time of occurrence

[Same as Item 2 but restricted to serious adverse events]

Item 6: Drug-related serious adverse events by body system

[Same as Item 1 but restricted to drug-related serious adverse events]

Item 7: Liver toxicity: ALT or AST is ≥3 times of upper limit of normal

	Rilulzole (N=.)	Placebo (N=.)
ALT or AST is ≥3 times of upper limit of normal n(%)		
No	. (%)	. (%)
Yes	. (%)	. (%)

Item 8 (Listing): Lab values for subjects with ALT or AST ≥3 times upper limit of normal

Subject	Visit	Test Date	ALT	AST	ALP	Total Bilirubin	INR
XXX-0000	baseline	XXNOV200X	.	.	.	.	.
…							

Item 9: Deaths

	Rilulzole (N=.)	Placebo (N=.)
Death n(%)		
No	. (%)	. (%)
Yes	. (%)	. (%)

Item 10 (Listing): AEs among deaths

Subject	AE Num.	System organ class<br>Preferred term<br>Reported term	Start date<br>Stop date<br>Continuing?	SAE?	Related to study?	Outcome<br>Action
XXX-0000	1	Gastrointestinal disorders/<br>Dysphagia/<br>mild discomfort swallowing	XXNOV200X/<br>./<br>no	no	Unrelated	Resolved, no residual effects/<br>Concomitant medication
…						

Item 11: Hypotensive AEs

	Rilulzole (N=.)	Placebo (N=.)
Any Hypotensive AE[1] n(%)		
No	. (%)	. (%)
Yes	. (%)	. (%)

[1] Coded by un-blinded monitor

Item 12 (Listing): Hypotensive AEs

[Same format as Item 10 but restricted to Hypotensive AEs]

Item 13: Demographics

	Rilulzole (N=.)	Placebo (N=.)
Age at consent (years)		
N	.	.

	**Rilulzole (N=.)**	**Placebo (N=.)**
Mean	.	.
SD	.	.
Median	.	.
Range	. - .	. - .
Gender, n (%)		
Female	. (%)	. (%)
Male	. (%)	. (%)

Item 14: ASIA grade at baseline

	**Rilulzole (N=.)**	**Placebo (N=.)**
ASIA Grade, n (%)		
A	. (%)	. (%)
B	. (%)	. (%)
C	. (%)	. (%)
D	. (%)	. (%)
E	. (%)	. (%)

Item 15: ASIA motor score total at baseline

	**Rilulzole (N=.)**	**Placebo (N=.)**
ASIA motor score total		
N	.	.

	**Rilulzole (N=.)**	**Placebo (N=.)**
Mean	.	.
SD	.	.
Median	.	.
Range	. - .	. - .

Item 16: ASIA neurological level at baseline

	**Rilulzole (N=.)**	**Placebo (N=.)**
ASIA Level, n (%)		
C1	. (%)	. (%)
C2	. (%)	. (%)
C3	. (%)	. (%)
C4	. (%)	. (%)
C5	. (%)	. (%)
…		

13.4. *Appendix 4 DSMB Recommendation Letter*

Protocol No. SPN-12-001

DATA SAFETY MONITORING BOARD (DSMB)

RECOMMENDATION LETTER

From: *Dr. Anthony Burns,* DSMB Chairperson

To: Nor Consult LLC.

The DSMB charged with the review of accumulating safety data for Protocol SPN-12-001 met on
___/____/____
(DD/MMM/YYYY)

Based on our review, we recommend (please check):

☐ Continuation of study without modification

☐ Continue the study and amend the protocol, as described below

☐ Pause enrollment, pending resolution of a specific issue, as described below

☐ Discontinuation of study, for reasons as described below

Rationale:____

____

____

____

Additional Comments:

____

____

____

____

____

Signature: ____________________________ Date: ____/______/_______
DSMB Chairperson (DD/MMM/YYYY)
