# Supplementary material for: Safety and Efficacy of Riluzole in Acute Spinal Cord Injury Study (RISCIS): A Multi-Center, Randomized, Placebo-Controlled, Double-Blinded Trial
Source: J Neurotrauma. 2023 Aug 23;40(17-18):1878–88. doi: 10.1089/neu.2023.0163 (PMC10460693; doi:10.1089/neu.2023.0163)
Supplement: Supplemental data [file 11DSMBRecommendation.pdf]

13.4 Appendix 4 DSMB Recommendation Letter

Protocol No. SPN-12-001

DATA SAFETY MONITORING BOARD (DSMB)

RECOMMENDATION LETTER FOR SPONSOR

From: *Dr. Anthony Burns,* DSMB Chairperson

To: Nor Consult, LLC.

The DSMB charged with the review of accumulating safety data for Protocol SPN-12-001 met on 30/Apr/2020. The primary investigator (PI), Michael G. Fehlings MD PhD, joined the DSMB for the beginning of the meeting (conference call). Dr. Fehlings provided an update that included (1) the recent decision of the trial sponsor, AO Spine Foundation, to suspend the trial, and (2) the accompanying intent to curtail the follow-up period for recent enrollees from the originally intended 12 months to 6 months. At the conclusion, Dr. Fehlings left the call after which the DSMB discussed the implications for study participants and additional stakeholders including the spinal cord injury (SCI) research community. The most recent safety data (March 2, 2020) was also reviewed and discussed.

Based on the above, the DSMB reached consensus and recommends the following:

- In the absence of the recent funding developments, the DSMB recommends continuation of the study without modification.

- Given that current study participants consented to a study protocol which outlined a 12 month follow-up period, there is an obligation from a safety, scientific, and ethical perspective to complete the originally intended 12 month follow-up for active participants.

- Given the scientific importance of the study and accompanying financial and logistical investments to date, it is important to maximize and disseminate learnings to external stakeholders (i.e., research community). To accomplish this, the DSMB recommends the completion of an independent interim analysis as previously outlined in the study protocol. This should be performed in a manner that would

not compromise resumption of the study, with appropriate modifications (e.g., adjustment of target enrollment), if additional funding were to become available.

Additional Comments:

Dr. Carlo Santaguida was not present for the April 30, 2020 meeting due to a delay in the operating room. A follow-up call between Drs. Burns and Santaguida took place on 01/May/2020. During the call and accompanying discussion, it was confirmed that Dr. Santaguida agrees with the recommendations of his DSMB colleagues as discussed on April 30, 2020, and outlined above.

Signature: ______________________ Date: 06 /MAY/2020
DSMB Chairperson (DD/MMM/YYYY)
